# Synthesis and Nuclear Magnetic Resonance Structural Evaluation of Oxime-Linked Oligosialic Acid-Based Glycodendrimers

**DOI:** 10.1021/acs.biomac.3c00105

**Published:** 2023-03-29

**Authors:** James P. Cerney, Aleksey Raskovalov, Monica Nasseri, Madeline D. Silva, Katherine D. McReynolds

**Affiliations:** Department of Chemistry, California State University, Sacramento 6000 J Street, Sacramento, California 95819-6057, United States

## Abstract

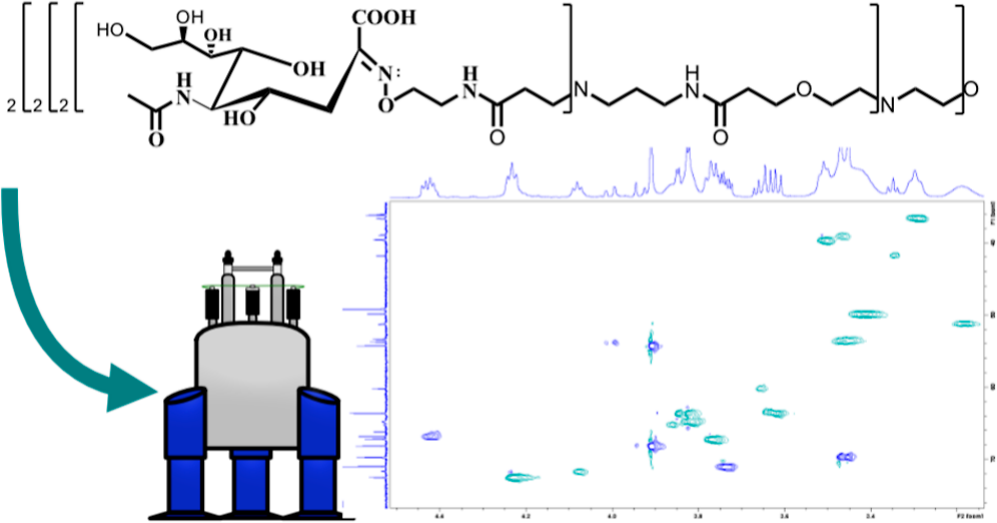

A series of four oxime-linked octavalent sialic acid and oligosialic acid poly(ether amidoamine) glycodendrimers were synthesized. In the attachment of the sialic acids to the dendrimer core, chemoselective oxime bonds were formed between the unprotected sugars (sialic acid or α-2,8-linked di- through tetra-sialic acids) and the aminooxy-terminated dendrimer core in a microwave-mediated reaction, resulting in good to excellent yields (58–100%) of the fully functionalized octavalent glycodendrimers. Next, using a combination of 1D and 2D nuclear magnetic resonance and working from the inside outward, we employed a systematic method to assign the proton and carbon signals starting with the smallest linkers and dendrimer cores and moving gradually up to the completed octavalent glycodendrimers. Through this approach, the assignment of the protons and carbons was possible, including the *E*- and *Z*-isomers related to the oxime dendrimer to sugar connections and relative quantities of each. These glycodendrimers were designed as broad-spectrum inhibitors of viral pathogens.

## Introduction

Sialic acids are a significant class of mono-, oligo-, and polysaccharides with a variety of biological functions. They are present as non-reducing terminal sugars on cell-surface glycoproteins and are implicated in a variety of disease states, including bacterial and viral pathogenesis, autoimmune disorders, and the progression of cancerous states, to name a few.^1^ Given their biological importance, it is critical that scientists continue to study sialic acid-containing molecules, both natural and synthetic, to further our understanding of these important sugars and their roles in biological processes.

To this end, our lab is focused on the development of polymers with carbohydrate surface modifications to serve as broad-spectrum topical anti-viral agents. This is particularly important at present, given the current dual pandemics caused by the zoonotic viruses, HIV-1 (human immunodeficiency virus type 1), and SARS-CoV-2 (severe acute respiratory syndrome coronavirus 2).^2^ Despite having many treatment options as well as the means to prevent new HIV infections, there were still 1.5 million new infections reported in 2021 as well as 650,000 deaths resulting from acquired immunodeficiency syndrome (AIDS). AIDS is responsible for a total of 40.1 million deaths since 1981, with 38.4 million individuals living with HIV/AIDS in 2021.^3^ For SARS-CoV-2, which was first observed in late 2019, there have been nearly 1 billion million people reported infected and over 6.8 million deaths caused by this fast-moving virus.^4^ Interestingly, while there is still no approved vaccine available to prevent HIV after more than 40 years of research, for SARS-CoV-2, a worldwide effort was launched in the spring of 2020 to develop a vaccine, which has resulted in multiple effective vaccines against SARS-CoV-2. Unfortunately, even with multiple vaccines against SARS-CoV-2 available and preventative measures such as condoms and PrEP (pre-exposure prophylaxis) to protect against HIV, there are still new infections and deaths resulting from both viruses.

Due to the continuing threat of the “twindemic” caused by HIV and SARS-CoV-2 as well as new emerging viral pathogens as yet unknown, we are working on the development of glycodendrimers as broad-spectrum topical anti-viral agents that can be employed to prevent the initial binding/infection of susceptible host cells. We currently lack broad-spectrum anti-viral agents and instead have many “one bug-one drug” types of therapeutics. This strategy is not effective when a new pathogen rapidly emerges and spreads, which is exactly what happened when SARS-CoV-2 exploded onto the global scene in late 2019. It is of the utmost importance that new broad-spectrum anti-viral agents are developed so that the next time a zoonotic viral spillover event occurs, such as what occurred with SARS-CoV-2, we are better prepared to not only stem the spread of disease but also mitigate the risk of severe illness and death.^5^

Viruses such as HIV and SARS-CoV-2 as well as many other pathogens utilize the ubiquitous cell surface heparan sulfate proteoglycans (HSPGs) in the early-stage binding of the surface glycoproteins such as gp120 and the S protein of HIV and SARS-CoV-2, respectively.^6^ HSPGs are important in normal cell functioning in many ways, including embryonic development, adult homeostasis, and aging.^7^ They are also implicated in many disease states, which includes serving as a coreceptor for virus concentration at the cell surface and presentation to the primary receptors, as is seen with HIV, SARS-CoV-2, and many other diverse types of viruses. It is this type of interaction that our research seeks to block through the design and synthesis of HSPG-analogues in the form of sulfated glycodendrimers. These molecules have the potential to bind to gp120 and the S protein and block the viruses from being able to recognize/bind to vulnerable host cells bearing the primary viral receptors, such as CD4^+^ helper T cells, as well as ACE-2-bearing epithelial tissues, in HIV- and SARS-CoV-2-mediated infection events, respectively.^6^

Earlier studies conducted in our laboratory provided proof of concept for this approach. Other groups have also explored glycodendrimers for these applications.^8^ For our previous work, a 16-valent sulfated sialic acid-terminated PAMAM (poly(amidoamine)) glycodendrimer was shown to have the ability to bind to gp120 through a simple ELISA (enzyme-linked immunosorbent assay), followed by IC_50_ (inhibitory concentration for 50% effect) values of 1.6–5.1 μM against four pseudovirus strains of HIV-1 in an inhibition of infectivity assay.^9^ A subsequent study found that common oligosaccharides such as lactose, once sulfated and attached to a water-soluble hexavalent dendrimer core, provided a 10-fold improvement in anti-viral activities against the same pseudoviruses (IC_50_ values 0.34–0.42 μM) despite being 60% smaller in size and bearing only 6 disaccharides rather than 16 monosaccharides.^10^ These findings collectively led us to design the current glycodendrimers as potential broad-spectrum anti-viral agents against both HIV and SARS-CoV-2 despite them being from different virus families. Here, we were presented with a significant challenge in being able to confirm the structures of these glycodendrimers despite having a synthetic strategy that was greatly improved in efficiency and yields over our previous sialic acid-bearing glycodendrimers.^9^ Given the large size and structural complexity of the glycodendrimers synthesized here, we set out to use both 1D and 2D NMR experiments to methodically characterize the molecules beginning with the smallest linker components, and working outward toward first, the dendrimer aminooxy termini, and then expanding into the glycodendrimers, starting with the sialic acid-terminated glycopolymer, and completing the process with the tetra-sialic acid-modified glycodendrimer. From here, these compounds will be assessed for their anti-viral activities against both HIV and SARS-CoV-2 to determine their efficacy as broad-spectrum anti-viral agents.

## Experimental Procedures

### General Information

All chemicals, including dry reaction solvents, unless noted, were purchased from commercial sources and used without further purification. A Bruker Avance III 500 MHz NMR spectrometer was used for all NMR experiments [^1^H, ^13^C, ^1^H–^1^H COSY (correlated spectroscopy) and ^1^H–^13^C heteronuclear single quantum coherence (HSQC)] using the standard experimental parameters provided by Bruker. NMR data were analyzed using a Bruker Topspin 4.0.7. Copies of all ^1^H–^1^H COSY and ^1^H–^13^C HSQC NMR spectra are in the Supporting Information. All reactions conducted under microwave irradiation were achieved using a CEM Discover SP microwave synthesizer. A freeze dry/shell freeze system (LabConCo, 7522800) was implemented for all lyophilization performed during the experimentation process. Dialysis was conducted using nano-pure water with Spectra/por Biotech cellulose ester dialysis membranes for Spectrum Laboratories Inc. Fast pace liquid chromatography (FPLC) was executed on a Bio-Gel P-10 column (2.5 × 120 cm) with a Bio-Rad BioLogic DuoFlow F10 Pumphead with a Dell computer controller. The elution chromatograms for the glycodendrimer products are provided in the Supporting Information. Reverse-phase high-pressure liquid chromatography (RP-HPLC) was performed on a Hewlett Packard TI-series 1050 with a Grace Prevail preparative C-18 5 μM column (10 × 250 mm). Electrospray ionization mass spectrometry (ESI–MS) was carried out at The Ohio State University, Mass Spectrometry and Proteomics Facility. The ESI–MS spectra are available in the Supporting Information.

### Synthesis

#### 3-(2-Hydroxyethoxy)propanenitrile (Compound **1**)

This was prepared according to our previously published work.^11^ Briefly, to a 100 mL round-bottomed flask, ethylene glycol (26.0 g, 419 mmol) was added followed by 0.3 mL of a freshly prepared 10% (w/v) potassium hydroxide solution. The reaction mixture was placed in an oil bath set to 45 °C and was allowed to stir for 60 min. The reaction was started through the addition of acrylonitrile (4.80 g, 90.4 mmol) over a 20 min time period to promote a slower reaction, thereby leading to a higher amount of monosubstituted product. The reaction was halted after 5 h through evaporation under reduced pressure, removing any water and leftover acrylonitrile. The crude extract was then purified via flash chromatography on silica gel and a mobile phase of 9:1 ethyl acetate/hexanes, resulting in a pure, clear colorless oil (9.25 g, 80.3 mmol, 89%). ^1^H NMR (500 MHz, D_2_O): δ 3.80 (t, *J* = 6.00 Hz, 2H), 3.75 (t, *J* = 5.00 Hz, 2H), 3.68 (t, *J* = 5.00 Hz, 2H), 2.80 (t, *J* = 6.00 Hz, 2H). ^13^C NMR (125 MHz, D_2_O, internal MeOH std): δ 120.00, 71.83, 65.39, 60.42, 18.19.

#### 2-(2-Cyanoethoxy)ethyl *p*-Toluenesulfonate (Compound **2**)

A 50 mL round-bottomed flask containing compound **1** (1.29 g, 11.2 mmol) was placed under nitrogen gas. The reaction was then initiated by the addition of 20 mL of dichloromethane and *p*-toluenesulfonyl chloride (TsCl, 3.49 g, 18.3 mmol). Pyridine (1.77 g, 22.4 mmol) was added to mark the start of the reaction. The reaction mixture was allowed to stir overnight at room temperature and was halted through extraction using chloroform with two washes of 1 M HCl and two washes of nano-pure water. The slightly impure oil was purified through flash chromatography on silica gel using 2:1 hexanes/ethyl acetate as the mobile phase, resulting in a clear hay-yellow oil, compound **2** (2.45 g, 9.10 mmol, 88%). ^1^H NMR (500 MHz, CDCl_3_): δ 7.78 (d, *J* = 8.50 Hz, 2H), 7.34 (d, *J* = 7.85 Hz, 2H), 4.15 (t, *J* = 4.70 Hz, 2H), 3.69 (t, *J* = 5.05 Hz, 2H), 3.62 (t, *J* = 6.30 Hz, 2H), 2.52 (t, *J* = 6.30 Hz, 2H), 2.43 (s, 3H). ^13^C NMR (125 MHz, CDCl_3_): δ 145.04, 132.62, 129.90, 127.84, 117.74, 69.04, 68.52, 65.75, 21.54, 18.66.

#### Boc-Ethanolamine (Compound **3**)

To a 100 mL round-bottomed flask, ethanolamine (1.01 g, 16.6 mmol) was added and subsequently placed under nitrogen gas. This was followed by the addition of 45 mL of methanol and 5 mL of triethylamine, turning the reaction mix from colorless to a yellow solution. The reaction was started through the addition of di-*tert*-butyl-dicarbonate [(Boc)_2_O, 4.12 g, 18.8 mmol], and the reaction mix then was allowed to stir overnight at room temperature. The reaction was halted through evaporation under reduced pressure, resulting in the quantitative yield of a pure mahogany-colored oil, compound **3**. ^1^H NMR (500 MHz, D_2_O): δ 3.63 (t, *J* = 5.65 Hz, 2H), 3.23 (t, *J* = 5.35 Hz, 2H), 1.46 (s, 9H). ^13^C NMR (125 MHz, D_2_O, internal MeOH std): δ 158.72, 81.36, 60.79, 42.43, 27.99.

#### 2-(Boc-amino)-1-*N*-hydroxysuccinimidyl Ethanol (Compound **4**)

A round-bottomed flask containing compound **3** (1.57 g, 9.70 mmol) was placed under nitrogen gas, followed by the addition of *N*-hydroxysuccinimide (HOSu, 1.23 mg, 10.70 mmol) and triphenylphosphine (PPh_3_, 2.81 g, 10.70 mmol). Next, 40 mL of tetrahydrofuran was added, and the reaction was started through the slow addition of diisopropyl azodicarboxylate (2.18 g, 10.70 mmol). The reaction was halted via evaporation under reduced pressure, and the residue was extracted using chloroform with three washes of nano-pure water. The organic layer was kept and evaporated under reduced pressure. The crude oil then underwent further purification via flash chromatography in silica gel using 2:1 ethyl acetate/hexanes, resulting in an oily white solid. The reaction byproduct, triphenylphosphine oxide, co-eluted with the desired product; therefore, all of the fractions containing compound **4** were collected and carried forward without any further purification.

#### 2-Amino-1-*N*-hydroxysuccinimidyl Ethanol (Compound **5**)

A round-bottomed flask containing the crude mix of compound **4** (3.56 g) was placed under nitrogen gas, and 2 mL of dichloromethane was added. Once all the solid had fully dissolved, the reaction was started through the addition of 2 mL of trifluoroacetic acid (TFA). The mix was allowed to stir for 2 h and was halted via evaporation under reduced pressure. The crude oil was then extracted using nano-pure water with three rinses of chloroform. The aqueous layer was kept and underwent lyophilization, revealing the pure white solid, compound **5** (1.32 g, 4.84 mmol) in a two-step yield of 50%. ^1^H NMR (500 MHz, D_2_O): δ 4.39 (t, *J* = 4.76 Hz, 2H), 3.37 (t, *J* = 4.73 Hz, 2H), 2.83 (s, 4H). ^13^C NMR (125 MHz, D_2_O, internal MeOH std): δ 175.72, 73.43, 38.20, 25.59.

#### Tetra-Nitrile Core (Compound **6**)

To a two-neck, 50 mL round-bottomed flask, 20 mL of acetonitrile was added, followed by placing the flask under nitrogen gas. Next, potassium carbonate (K_2_CO_3_, 3.21 g, 23.2 mmol) was added, and the solution was placed in an oil bath heated to 45 °C. The solution was stirred for 20 min to allow the mixture to become basic. 2,2′-Oxybis(ethylamine) (43.2 mg, 0.415 mmol) was then added, followed by compound **2** (677.2 mg, 2.5 mmol), which marked the start of the reaction. This was allowed to stir for 7 days with daily monitoring of the pH to ensure that it stayed between 9 and 10. The reaction was then halted via evaporation under reduced pressure, removing all acetonitrile, leaving a crude oily solid mix. This was then purified through extraction using chloroform and one wash of saturated sodium bicarbonate, removing any left-over potassium carbonate, *p*-toluenesulfonic acid, and any partial substitution products. Next, the organic layer was washed with water followed by a wash with 6 M HCl. Any excess of compound **2** remained in the organic layer, which was removed. The aqueous layer was then neutralized through the careful addition of solid sodium bicarbonate until no bubbling was witnessed. This layer was washed with two washes of chloroform, and the organic layer was then evaporated under reduced pressure. This resulted in an 88.1% yield of compound **6** (365 mg, 0.263 mmol), a hay-yellow colored oil. ^1^H NMR (500 MHz, D_2_O): δ 3.77 (t, *J* = 5.99 Hz, 8H), 3.73 (t, *J* = 5.67 Hz, 8H), 3.66 (t, *J* = 5.67 Hz, 4H), 2.87 (m, 12H), 2.81 (t, *J* = 5.99 Hz, 8H). ^13^C NMR (125 MHz, D_2_O, internal MeOH std): δ 120.23, 68.65, 68.44, 65.52, 53.53, 53.22, 18.44. HR–ESI [M + H]^+^ (C_24_H_40_N_6_O_5_) calcd *m*/*z*: 493.313295; found *m*/*z*: 493.31284 [M + H]^+^.

#### Tetra-Methyl Ester Core (Compound **7**)

A 25 mL round-bottomed flask containing compound **6** (129.7 mg, 0.263 mmol) was placed under nitrogen gas to which 5 mL of methanol was added. The reaction was next placed in an ice bath chilled to 0 °C, and the reaction was then started through the slow addition of 1.6 mL of acetyl chloride. This was allowed to stir for 3 days at 0 °C. The reaction was halted with the addition of 1.6 mL of nano-pure water, thereby allowing for the full conversion to the methyl ester-functionalized dendrimer. The crude mixture was then evaporated under reduced pressure, followed by lyophilization to remove any and all remaining acetyl chloride and was carried forth without any further purification. Compound **7** was confirmed using infrared (IR) spectroscopy through the loss of the C≡N stretch at 2248 cm^–1^ and the presence of the C=O stretch at 1723 cm^–1^, representing the newly formed methyl ester.

#### Tetra-Amino Core (Compound **8**)

A 100 mL round-bottomed flask containing crude compound **7** (161 mg) was placed under nitrogen gas. The reaction was started with the addition of 1,3-diaminopropane (3.70 g, 49.9 mmol). The mixture was stirred for 5 days, at which time the reaction was evaporated under reduced pressure using a methanol and toluene azeotrope to remove as much of the excess 1,3-diaminopropane as possible. This resulting oil was purified via RP-HPLC on a C-18 5 μM column, leading to compound **8** (135.3 mg, 0.109 mmol), a pale yellow oil, in a two-step yield of 42%. ^1^H NMR (500 MHz, D_2_O): δ 3.89 (t, *J* = 4.40 Hz, 4H), 3.84 (t, *J* = 4.40 Hz, 8H), 3.78 (t, *J* = 6.25 Hz, 8H), 3.52 (m, 20H), 3.28 (t, *J* = 6.60 Hz, 8H), 3.00 (t, *J* = 7.55 Hz, 8H), 2.55 (t, *J* = 5.70 Hz, 8H), 1.87 (p, *J* = 8.20, 6.30 Hz, 8H). ^13^C NMR (125 MHz, D_2_O, internal MeOH std): δ 174.05, 67.16, 64.90, 64.35, 53.64, 53.39, 37.29, 36.42, 36.04, 26.93. HR–ESI [M + 2H]^2+^ (C_36_H_76_N_10_O_9_) calcd *m*/*z*: 397.29711; found *m*/*z*: 397.29678 [M + 2H]^2+^.

#### Octa-Carboxyl Core (Compound **9**)

A 10 mL round-bottomed flask containing compound **8** (135.6 mg, 0.109 mmol) was attached to a condenser set to 10 °C and placed under nitrogen gas. Due to the large differences in polarity between the starting materials, 1 mL of methanol was added to ensure a homogeneous mix. Once all the compound was dissolved, *tert*-butyl acrylate (2.80 g, 21.8 mmol) was added followed by 1 mL of *N*,*N*-diisopropylethylamine (700 mg, 5.40 mmol) to start the reaction. The pH was monitored over the course of 5 days, ensuring that the pH remained at 10. The reaction was halted via evaporation under reduced pressure followed by purification through extraction using chloroform with two washes of water and one wash with saturated sodium chloride. The organic layer was kept, and the extract was taken forward without any further purification. The crude extract was then placed under nitrogen, and 2 mL of methylene chloride was added followed by 2 mL of TFA to start the reaction. This was allowed to react for 2 h and was halted by evaporation under reduced pressure followed by lyophilization. The mix was purified via extraction using water and three washes of chloroform. The aqueous layer was kept and lyophilized, leading to compound **9** (186 mg, 0.136 mmol), a light, sun-yellow oil in a two-step quantitative yield. ^1^H NMR (500 MHz, D_2_O): δ 3.87 (t, *J* = 4.70 Hz, 4H), 3.82 (t, *J* = 4.45 Hz, 8H), 3.76 (t, *J* = 6.00 Hz, 8H), 3.51 (m, 12H), 3.32 (t, *J* = 6.90 Hz, 8H), 3.26 (t, *J* = 7.90 Hz, 8H), 2.92 (t, *J* = 6.30 Hz, 16H), 2.57 (t, *J* = 6.15 Hz, 8H), 2.02 (p, *J* = 6.60, 7.55 Hz, 8H). ^13^C NMR (125 MHz, D_2_O, internal MeOH std): δ 174.54, 174.25, 67.12, 64.92, 64.36, 53.64, 53.40, 51.55, 49.34, 36.32, 36.02, 28.49, 23.61. HR–ESI [M + H]^+^ (C_60_H_108_N_10_O_25_) calcd *m*/*z*: 1369.755985; found *m*/*z*: 1369.75500 [M + H]^+^.

#### Octa-Aminooxy Core (Compound **10**)

A round-bottomed flask containing compound **9** (35.4 mg, 0.026 mmol) was placed under nitrogen gas followed by the addition of 1 mL of dimethyl sulfoxide (DMSO). Next, compound **5** (70.3 mg, 0.260 mmol) and *N*,*N*,*N*′,*N*′-tetramethyl-*O*-(benzotriazole-1-yl)uranium tetrafluoroborate (TBTU, 74.7 mg, 0.230 mmol) were added and allowed to thoroughly dissolve. The reaction was started with the addition of 1 mL of DIPEA to ensure a basic pH of 9. The reaction mixture was allowed to stir for 2 days at room temperature and was halted through the addition of water followed by continual lyophilization to remove any remaining DMSO. The crude mix was taken forward without any further purification. A 25 mL round-bottomed flask containing the crude mix was placed under nitrogen gas, and 2 mL of methanol was added. To start the reaction, hydrazine monohydrate (855 mg, 170.8 mmol) was added, and the reaction was allowed to stir for 12 h. The reaction was halted via evaporation under reduced pressure followed by lyophilization to remove any leftover hydrazine. The mixture was purified through FPLC, resulting in 24.0 mg of compound **10** for a two-step yield of 51%. ^1^H NMR (500 MHz, D_2_O): δ 4.15 (t, *J* = 5.10 Hz, 16H), 3.87 (t, *J* = 4.35 Hz, 4H), 3.82 (t, *J* = 4.45 Hz, 4H), 3.76 (t, *J* = 6.25 Hz, 4H), 3.54–3.46 (m, 4H), 3.29 (t, *J* = 6.85 Hz, 4H), 3.24 (t, *J* = 8.15 Hz, 4H), 2.81 (t, *J* = 6.60 Hz, 4H), 2.55 (t, *J* = 6.00 Hz, 4H), 1.99 (p, *J* = 7.25 Hz, 4H). ^13^C NMR (125 MHz, D_2_O, internal MeOH std): δ 174.40, 172.41, 73.80, 67.16, 64.93, 64.39, 51.42, 49.71, 37.53, 36.49, 36.06, 28.89, 23.58. HR–ESI [M + 2H]^2+^ (C_76_H_156_N_26_O_25_) calcd *m*/*z*: 917.594024; found *m*/*z*: 917.59229 [M + 2H]^2+^.

### General Procedure for the Microwave-Mediated Synthesis of Glycodendrimers

A pear-shaped flask containing compound **10** (1 equiv) was first suspended in 1.5 mL of a 0.15 M ammonium acetate buffered solution held at a pH of 4.5. Next, sialic acid or the appropriate sialic acid oligomer (monomer–tetramer, 9–10 equiv) was added. Finally, 14 μL of aniline (0.15 M) was added to the reaction mix. The reaction vessel was then connected to a Vigreux column and placed in the microwave unit. The reaction mixture was irradiated at 7 W and held for 6 h at a temperature of 54 °C. The reaction was halted through lyophilization followed by purification via filtration through a 0.45 μm filter and FPLC, resulting in pure fluffy white solids. (Sialic acid)_8_ (compound **11**): this reaction resulted in 20.5 mg (0.005 mmol, 58%) of the product. ^1^H NMR calculated for 1/4 of the molecule (500 MHz, D_2_O): δ 4.42–4.44 (m, 2H), 4.23 (t, *J* = 5.19 Hz, 3H), 4.08 (t, *J* = 4.95 Hz, 1H), 4.01 (d, *J* = 10.40 Hz, 1H), 3.95–3.91 (m, 3H), 3.80–3.89 (m, 7H), 3.72–3.78 (m, 5H), 3.61–3.65 (m, 2H), 3.39–3.52 (m, 16H), 3.30 (broad s, 3H), 3.19 (broad s, 3H), 2.69–2.79 (m, 8H), 2.56 (t, *J* = 5.65 Hz, 3H), 2.48 (d, *J* = 8.00 Hz, 1H), 2.08 (s, 6H), 1.98 (broad s, 2H). ^13^C NMR (125 MHz, D_2_O, internal MeOH std): δ 174.78(*E*), 174.75(*Z*),174.39, 172.20, 170.65(*Z*), 170.36(*E*), 158.15(*Z*), 156.50(*E*), 72.46(*E*), 71.61(*Z*), 70.96, 69.72(*E*), 69.67(*Z*), 68.11, 67.16, 66.78(*E*), 66.13(*Z*), 65.21, 64.72, 63.57, 54.2(*E*), 53.70(*Z*), 53.34, 51.27, 49.80, 39.54(*E*), 38.89(*Z*), 36.57, 36.15, 36.05(*Z*), 31.23(*E*), 29.45, 23.98, 22.27(*E*), 22.23(*Z*). HR–ESI [M + 3H]^3+^ (C_164_H_292_N_34_O_89_) calcd *m*/*z*: 1388.319552; found *m*/*z*: 1388.31916 [M + 3H]^3+^. (α-2 → 8 Linked di-sialic acid)_8_ (compound **12**): this reaction resulted in 18.3 mg (0.003 mmol, 92%) of the product. ^1^H NMR calculated for 1/4 of the molecule (500 MHz, D_2_O): δ 4.47–4.42 (m, 2H), 4.25 (bs, 3H), 4.10 (bs, 1H), 4.00 (d, *J* = 12.00 Hz, 1H) 3.96–3.79 (m, 22H), 3.72–3.64 (m, 8H), 3.58 (d, *J* = 9.10 Hz, 2H), 3.53 (bs, 3H), 3.48 (bs, 2H), 3.39–3.30 (m, 11H), 3.11 (bs, 2H), 2.82–2.69 (m, 9H), 2.58 (bs, 3H), 2.48 (d, *J* = 6.60 Hz, 1H), 2.10 (s, 6H), 2.05 (s, 6H), 1.79 (t, *J* = 12.40 Hz, 2H). ^13^C NMR (125 MHz, D_2_O, internal MeOH std): δ 175.39, 175.26, 174.71, 174.30, 173.86, 172.69, 170.66, 170.30, 158.17, 156.68, 102.21, 102.17, 74.71, 74.63, 73.09, 72.47, 72.14, 71.67, 68.55, 68.41, 68.14, 67.89, 67.15, 66.76, 66.13, 65.62, 65.25, 63.03, 61.52, 54.06, 53.69, 53.33, 52.05, 51.13, 49.69, 40.46, 39.39, 38.91, 36.82, 36.08, 31.26, 29.99, 24.16, 22.38, 22.31. (α-2 → 8 Linked tri-sialic acid)_8_ (compound **13**): this resulted in 32.4 mg (0.0037 mmol, 97%) of the desired product. ^1^H NMR calculated for 1/4 of the molecule (500 MHz, D_2_O): δ 4.47–4.41 (m, 2H), 4.24 (bs, 3H), 4.11 (bs, 5H), 4.00–3.78 (m, 25H), 3.72–3.56 (m, 16H), 3.52–3.47 (m, 14H), 3.32 (bs, 3H), 3.25 (bs, 3H), 2.81–2.75 (m, 8H), 2.72–2.65 (m, 3H), 2.59 (bs, 3H), 2.48–2.46 (d, *J* = 2.47 Hz, 1H), 2.09–2.01 (m, 19H), 1.77–1.71 (m, 3H). ^13^C NMR (125 MHz, D_2_O, internal MeOH std): δ 175.19, 175.15, 174.92, 174.64, 174.34, 173.58, 179.95, 171.87, 170.60, 170.15, 158.13, 156.56, 102.41, 100.92, 78.11, 74.84, 73.86, 72.91, 72.42, 72.06, 71.61, 69.24, 68.70, 68.40, 68.31, 68.15, 68.00, 67.92, 67.13, 66.63, 64.92, 64.40, 62.88, 61.94, 61.55, 61.28, 54.00, 53.68, 53.39, 52.75, 52.03, 51.26, 49.83, 40.58, 40.48, 39.38, 38.87, 36.46, 36.13, 36.00, 31.18, 29.15, 23.71, 22.62, 22.35, 22.29. (α-2 → 8 Linked tetra-sialic acid)_8_ (compound **14**): this reaction resulted in 30.4 mg (0.003 mmol, quantitative) of the product. ^1^H NMR calculated for 1/4 of the molecule (500 MHz, D_2_O): δ 4.47 (bs, 2H), 4.26 (bs, 3H), 4.20–4.12 (m, 8H), 4.01 (d, *J* = 2.47 Hz, 1H) 3.97–3.81 (m, 33H), 3.72–3.51 (m, 41H), 3.34 (bs, 4H), 3.29 (bs, 4H), 2.84–2.67 (m, 15H), 2.61 (bs, 4H), 2.49 (d, *J* = 2.47 Hz, 1H), 2.11–2.06 (m, 26H), 1.82–1.71 (m, 5H). ^13^C NMR (125 MHz, D_2_O, internal MeOH std): δ 175.19, 174.93, 174.64, 174.35, 173.82, 171.87, 170.63, 102.18, 78.51, 77.69, 74.68, 73.99, 73.04, 72.89, 72.44, 72.03, 71.55, 71.13, 69.65, 68.49, 68.44, 68,33, 68.27, 67.12, 66.72, 66.04, 65.71, 64.93, 64.40, 62.99, 62.89, 61.95, 61.57, 54.00, 53.69, 53.41, 52.80, 52.62, 51.35, 49.86, 40.62, 40.41, 40.25, 39.38, 38.88, 36.36, 36.13, 36.02, 31.19, 29.17, 23.72, 22.72, 22.61, 22.35.

## Results and Discussion

### Octavalent Aminooxy-Functionalized Dendrimer Core Synthesis

**Scheme 1:**
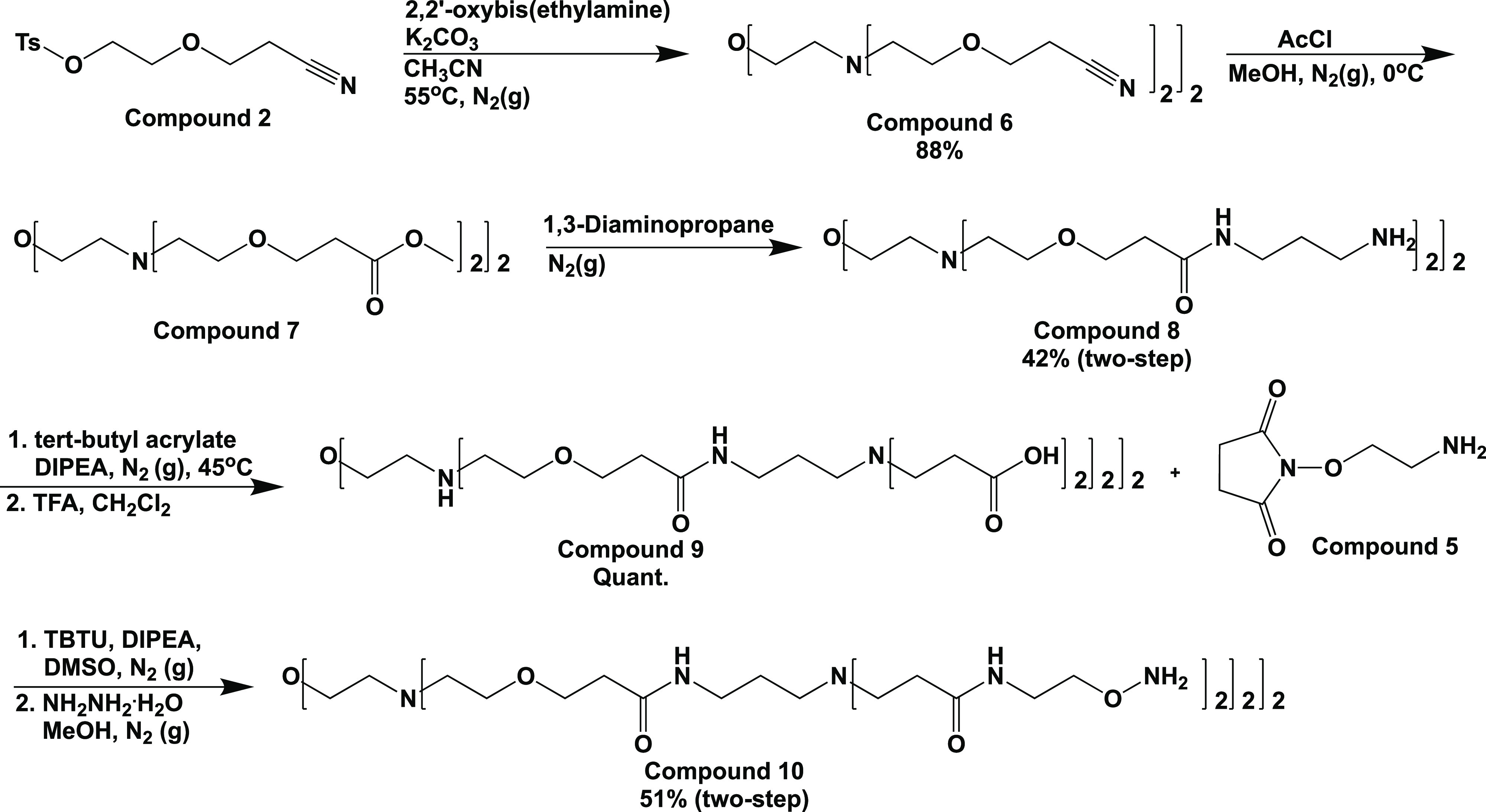
Synthesis of the Octavalent Aminooxy-Terminated Dendrimer Core, Compound **10**

In order to create sialic acid-/oligosialic acid-terminated glycodendrimers using little/no protecting group chemistry on the sugars and to employ a sugar conjugation reaction that could be chemoselective and conducted in aqueous media, it was essential that the dendrimer core possessed reactive ends compatible with the carbohydrates as well as a structure amenable to water-based reactions. Here, we settled on a poly(ether amidoamine) dendrimer core terminating in aminooxy groups, compound **10**. This core would allow us to attach the unprotected mono- and oligosaccharides in aqueous solution using a chemoselective oxime-forming reaction between the dendrimer aminooxy groups and the anomeric ketone groups present on the reducing ends of the sugars. The use of such a strategy to attach unprotected sugars to form other glycoconjugates such as glycopeptides has been previously reported.^12^ To begin the divergent synthesis of the dendrimer core, we started with a hydroxynitrile linker previously synthesized in our lab (compound **1**),^11^ which was first activated at the hydroxyl group using a tosylation reaction, giving compound **2** (Scheme 1). Compound **2** was then added to the commercially available bivalent core molecule, 2,2′-oxybis(ethylamine), to yield compound **6**, a tetra-nitrile core. The nitrile was then hydrolyzed to the methyl ester through treatment with acetyl chloride in methanol. Here, it was critical to carefully quench the reaction with a molar equivalent of water per end, such that terminal methyl esters (compound **7**) rather than carboxyl groups resulted. Once this was accomplished, amidation of the methyl esters was carried out through the neat addition of 1,3-diaminopropane to compound **7**, giving compound **8**, the tetra-amine core. Next, the terminal amines were branched using a Michael addition reaction with *t*-butyl acrylate in DIPEA, followed by deprotection with TFA in dichloromethane, providing the octa-carboxyl core molecule, compound **9**. Compound **9** was next functionalized with a short *N*-hydroxysuccinimide-functionalized linker, compound **5**, through a TBTU-mediated amidation reaction, followed by a hydrazinolysis reaction to reveal the completed octa-aminooxy core, compound **10**.

### 2D NMR Characterization of the Poly(ether amidoamine) Dendrimer Core

Given the structural complexity of the sialic acid-/oligosialic acid-terminated octavalent glycodendrimers, it was critical for us to take a methodical approach to the NMR characterization of the compounds at each stage of synthesis to allow for the complete and unambiguous assignments of both the protons and carbons contained within. To accomplish this, we used a combination of 1D and 2D NMR techniques, starting from our smallest linkers and tetravalent branched compounds and working our way outward with each step in the synthesis. This streamlined our characterization by allowing us to easily identify the signals from the earlier compounds and only necessitating the identification and assignment of the new signals after each synthetic step. This was particularly helpful once we started adding sugars to the aminooxy termini of the octavalent core, compound **10**, because of the addition of further overlapping signals and peak broadening for the interior dendrimer peaks.

**Figure 1:**
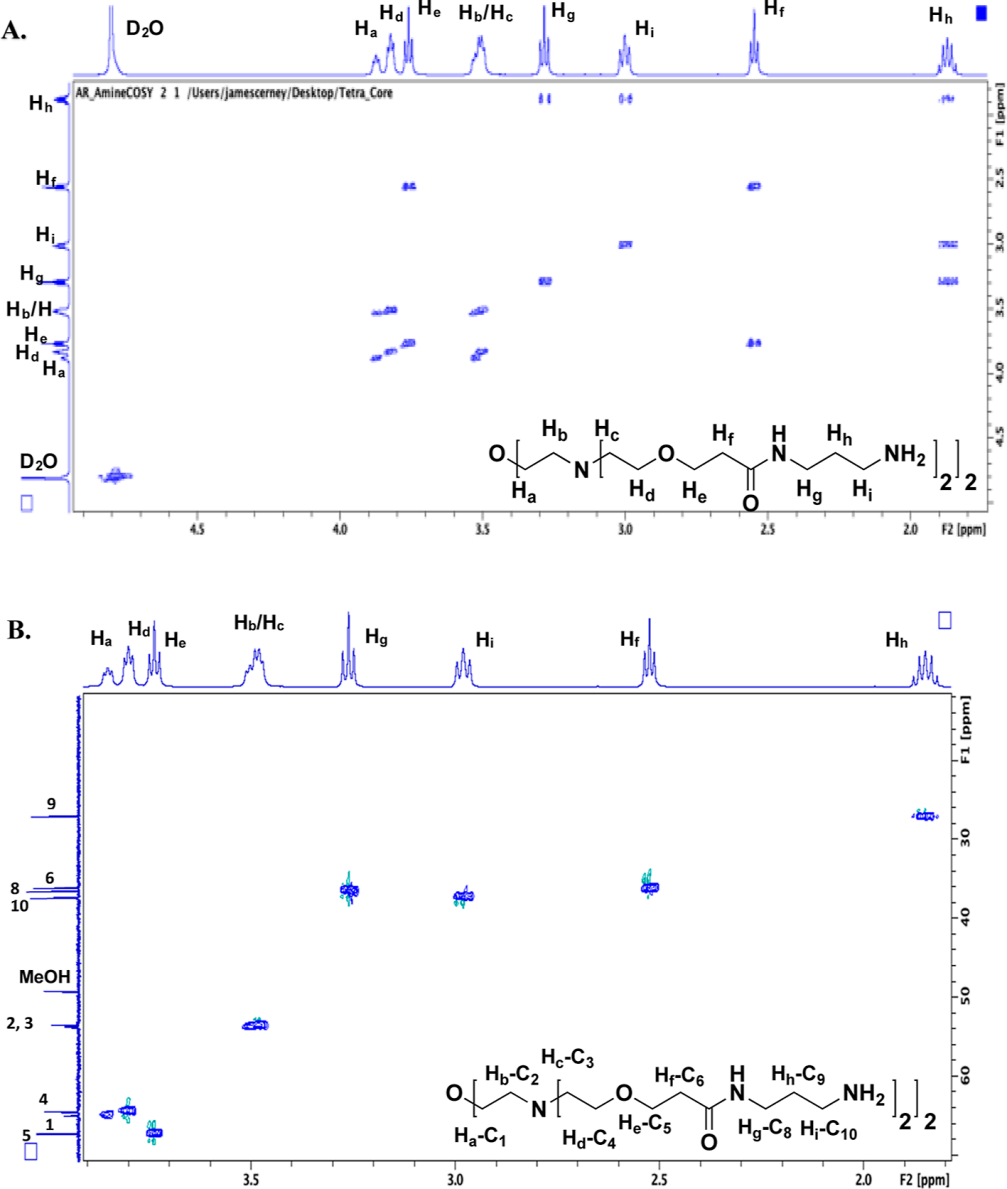
(A) COSY and (B) HSQC spectra for the tetra-amino dendrimer core, compound **8**, with the resultant ^1^H–^1^H and ^1^H–^13^C assignments shown, respectively. All full-sized spectra (^1^H, ^13^C, COSY, and HSQC), including integrations for the 1D ^1^H, are provided in the Supporting Information.

Here, we highlight our process on two key branched structures, the tetravalent amino core, compound **8**, and the octavalent aminooxy-terminated core, compound **10**. All other 1D and 2D NMRs are provided in the Supporting Information. For the ^1^H–^1^H COSY spectrum of compound **8** (Figure 1A), beginning with H_g_–H_i_, we can easily view the coupling between the two triplet signals, H_g_ and H_i_, with the intervening methylene signal labeled as H_h_. Moving further toward the interior of the molecule, clear coupling is noted between H_e_ and H_f_. Peak broadening increases as we move further inward to the center of the core due primarily to the lower level of solvation of these carbons. This contributes to increased relaxation times for internal groups. H_c_ and H_b_ are overlapping signals but are clearly coupled to H_d_ and H_a_, respectively. These assignments are further corroborated by the ^1^H–^13^C-coupling visualized through the HSQC experiment, shown in Figure 1B. Through the HSQC experiment, we have confirmation of not only the COSY assignments but also the connectivity of the protons to their respective carbons (C_1_–C_10_).

**Figure 2:**
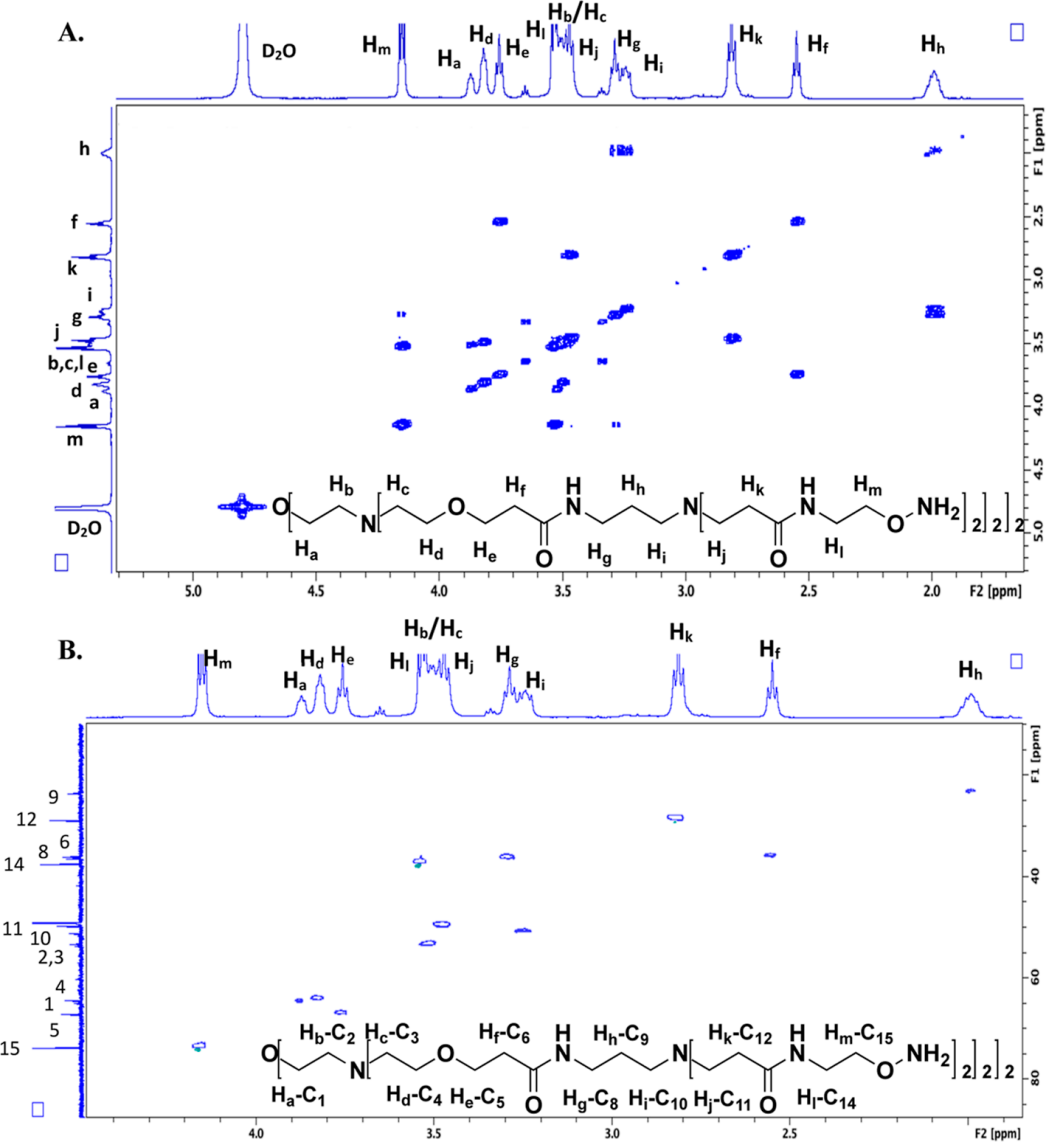
(A) COSY and (B) HSQC spectra for the octa-aminooxy dendrimer core, compound **10**, with the resultant ^1^H–^1^H and ^1^H–^13^C assignments shown, respectively. All full-sized spectra (^1^H, ^13^C, COSY, and HSQC), including integrations for the 1D ^1^H, are provided in the Supporting Information.

Upon completion of the synthesis of compound **10**, COSY and HSQC NMR analyses were also completed (Figure 2). Here, we have the addition of a new branch point, taking the molecule from tetravalent to octavalent, and new signals corresponding to H_j_–H_m_ and C_11_–C_15_. Beginning with the COSY spectrum (Figure 2A), the H_m_ signal is apparent at the most downfield position due to proximity to the aminooxy group. H_m_ shows strong coupling with H_l_, and moving toward the branch point, H_k_ couples to H_j_, which is part of a large overlapping region with signals coming from H_b_, H_c_, H_j_, and H_l_. All of the signals assigned from compound **8** are roughly where they were found previously. The HSQC (Figure 2B) was next examined to provide the ^1^H–^13^C connectivity. Starting at the outermost position of the dendrimer, the carbon attached to the aminooxy group, the most downfield ^1^H signal, H_m_, was not surprisingly attached to C_15_, the most downfield ^13^C signal. Moving to H_l_, adjacent to the nitrogen of the amide group, these methylene protons were found to be attached to C_14_. Moving to the carbonyl side of the amide, H_k_ was attached to C_12_, followed by H_j_ being linked to C_11_. C_13_, the amide carbonyl, was assigned from the 1D ^13^C spectrum.

### Synthesis of Octavalent Sialic/Oligosialic Acid-Terminated Glycodendrimers

**Figure 3:**
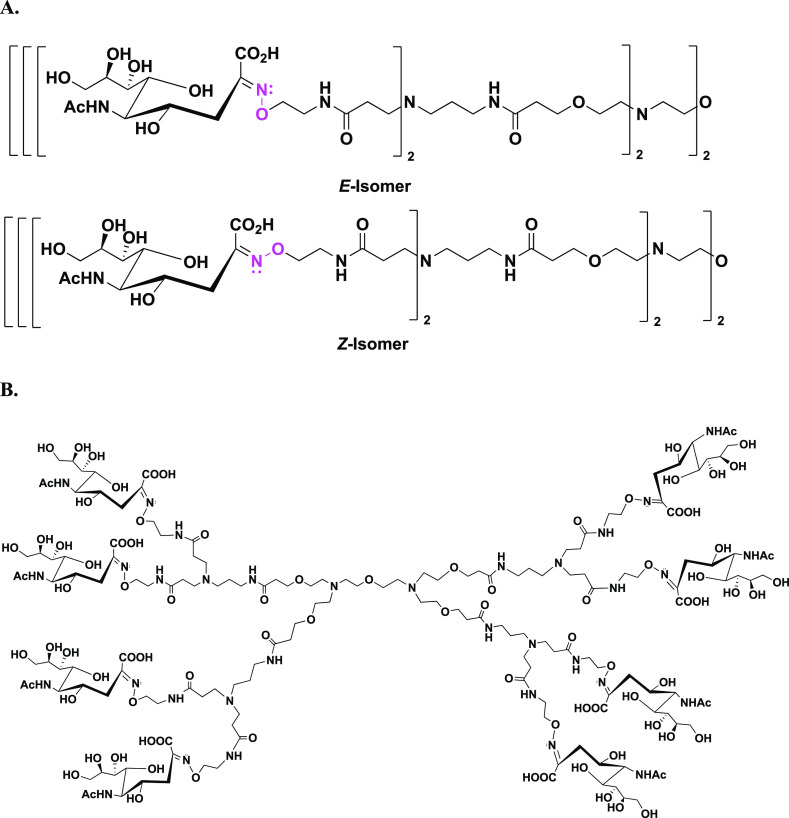
(A) Illustration of the *E*- (major) and *Z*-forms (minor) of the oximes formed between the octavalent aminooxy-terminated dendrimer core (compound **10**) and the reducing ketose, sialic acid, giving the sialic acid-terminated glycodendrimer, compound **11**. Not shown: closed ring α- and β-anomers (trace). (B) Full structure of compound **11**.

**Scheme 2:**
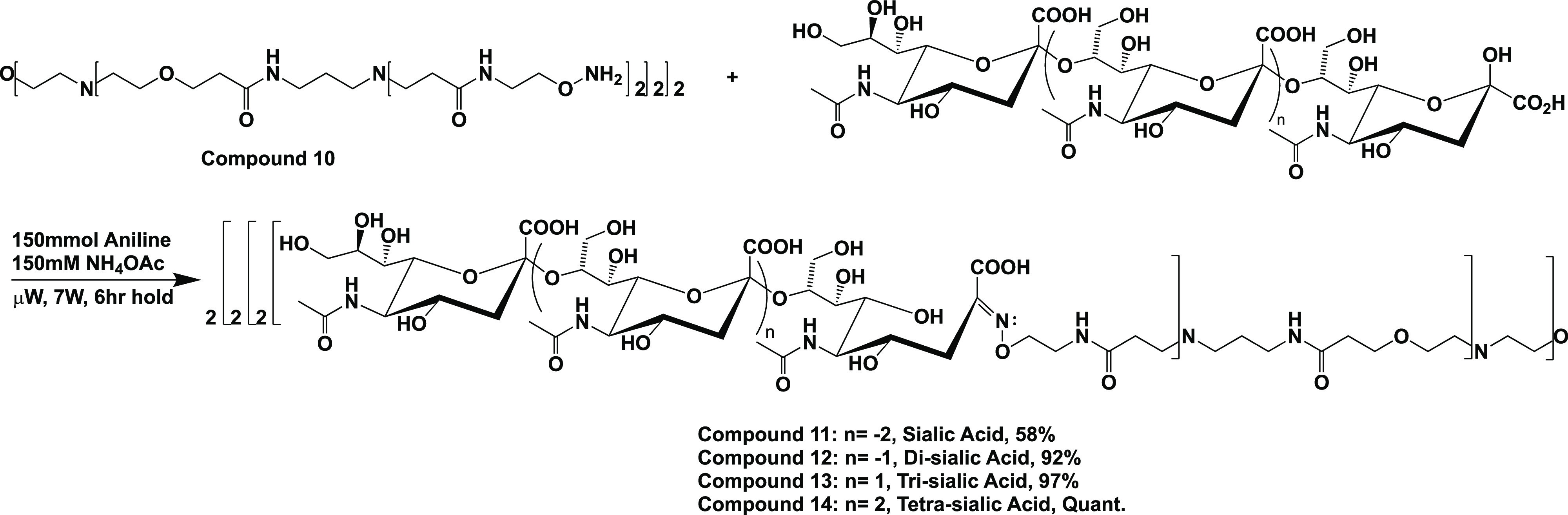
Microwave-Mediated Synthesis of the Four Sialic Acid-Terminated Glycodendrimers, Compounds **11–14**

Once the aminooxy-terminated dendrimer core, compound **10**, was synthesized and characterized, monomeric sialic acid through α-2,8-linked tetra-sialic acid oligomers was attached.^13^ This was accomplished through a microwave-mediated oxime coupling reaction in pH 4.5 ammonium acetate buffer and aniline as a catalyst (Scheme 2). After a 6 h microwave reaction, held at 54 °C, the four glycodendrimers were purified by FPLC to provide compounds **11–14** in % yields of 58, 92, 97, and quantitative, respectively. For the glycodendrimer products, it is important to recognize that there are multiple isomeric products possible, resulting from the oxime formation at the reducing end of the ketose sugar. These possible products range from the open ring *E*- (major) and *Z*-isomers (minor) to the closed ring α- and β-anomers (trace). The open chain *E*- and *Z*-isomers are shown in Figure 3A, where the conformation of the oxime is shown in pink as well as the complete structure of compound **11** (Figure 3B). The presence of multiple isomeric products significantly complicates the assignment of both the ^1^H and ^13^C signals. This is further discussed below.

### 2D NMR Characterization of Octavalent Sialic Acid-/Oligosialic Acid-Terminated Glycodendrimers

**Figure 4:**
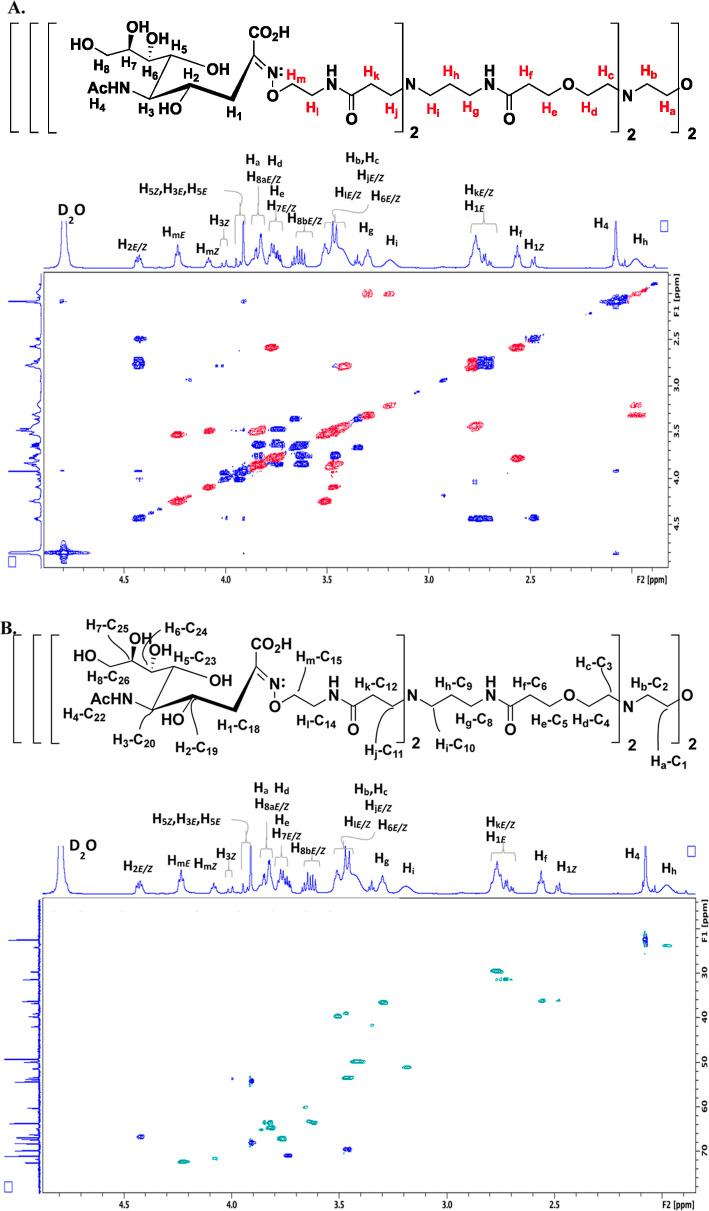
(A) COSY and (B) HSQC spectra for the (sialic acid)_8_-terminated glycodendrimer, compound **11**, with the resultant ^1^H–^1^H and ^1^H–^13^C assignments shown, respectively. (A) Red contours represent the dendrimer core signals, while the dark blue contours represent signals resulting from the sialic acid. (B) Light green contours represent CH_2_ groups, while the dark blue contours indicate CH or CH_3_ signals. All full-size spectra (^1^H, ^13^C, COSY, and HSQC), including integrations for the 1D ^1^H, are provided in the Supporting Information.

The addition of either a monomeric or an oligomeric sialic acid molecule to the aminooxy termini of compound **10** significantly complicated the analysis/peak assignments using 1D and 2D NMR. This was further muddled by the presence of multiple isomers, as discussed above, as well as peak broadening and overlap, common to glycodendrimer macromolecules. To accomplish complete peak assignment, we used a systematic approach of building the peak assignments from the inside to outside surface of the dendrimer/glycodendrimer. For the glycodendrimer NMR assignments, the dendrimer core peaks were assigned letters (H_a_–H_m_) and numbers (C_1_–C_15_) as before, and the sugar peaks were assigned numbers (H_1_–H_8_ and C_16_–C_25_ for compound **11**). We were also assisted in our assignments through a previous report by Szabo et al., where a short aminooxy linker was attached to the reducing end of both sialic acid and α-2,8-tetra-sialic acid.^14^ Beginning with the COSY for compound **11** (Figure 4A), the signals resulting from the dendrimer core peaks are shown in red, as previously assigned, while the new sialic acid signals are represented in dark blue. Interestingly, many of the dendrimer core peaks have become further broadened, to the point where splitting is no longer observed. The signals from the sugar and the linker in close proximity to the oxime linkage (H_1_–H_8_ and H_l_–H_m_) are affected by the *E*- and *Z*-configurations of the oxime and are labeled as such. Additionally, even though there is no proton attached to the anomeric carbon, which results in signature *E*- and *Z*-signals further downfield, we were able to estimate the *E*- and *Z*-isomer percentages using the linker H_m_ signal immediately adjacent to the oxime on the dendrimer core side, which provided clean triplets between 4.0 and 4.3 ppm. From the integrations, we ascertained that the *E*-isomer was present in ∼69% abundance, while the *Z*-isomer comprised ∼30% of the mixture. Besides H_m_, the only other signals that split into discrete peaks that could be identified as the *E*- vs *Z*-isomeric forms were the H_1_ peaks arising from the CH_2_ group at C_18_ on the sialic acid, found at ∼2.7 and 2.5 ppm, respectively, and H_3_, from the CH signal on C_20_, found at ∼3.9 and 4.0 ppm, respectively. Moving to the HSQC spectrum (Figure 4B), the dark blue signals represent an odd number of protons attached to carbon (CH or CH_3_) and the light green contours illustrate signals with an even number of protons attached to carbon (CH_2_). As all of the signals from the dendrimer core and only two signals from the sialic acid arise from CH_2_ groups (H_1_–C_18_ and H_8_–C_26_), the blue contours represent the sialic acid, H_2_–H_7_ signals, making assignment of the sialic acid portion of the glycodendrimer more facile, as the expected number of signals from the different types of protons (odd vs even) are clearly visible.

**Figure 5:**
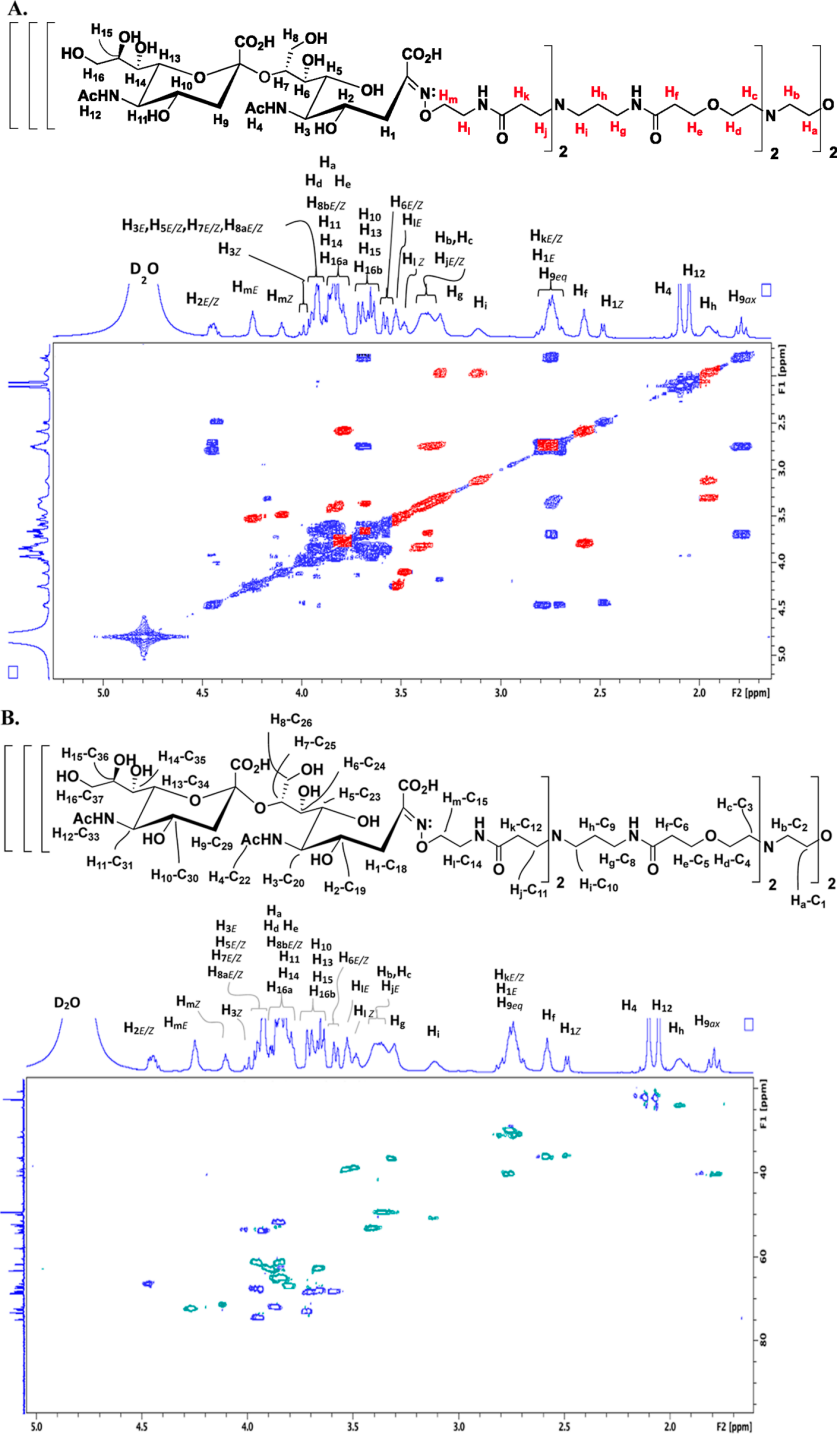
(A) COSY and (B) HSQC spectra for the (α-2 → 8 linked di-sialic acid)_8_-terminated glycodendrimer, compound **12**, with the resultant ^1^H–^1^H and ^1^H–^13^C assignments shown, respectively. (A) Red contours represent the dendrimer core signals, while the dark blue contours represent signals resulting from the sialic acid. (B) Light green contours represent CH_2_ groups, while the dark blue contours indicate CH or CH_3_ signals. All full-size spectra (^1^H, ^13^C, COSY, and HSQC), including integrations for the 1D ^1^H, are provided in the Supporting Information.

Similar to the assignment of the protons and carbons for compound **11**, compound **12** was assigned. Figure 5 shows the COSY (Figure 5A) and HSQC (Figure 5B) for the α-2,8-linked dimer-substituted glycodendrimer. Beginning with the COSY (Figure 5A), we have the addition of the H_9_–H_16_ protons arising from the non-reducing sialic acid. The signals resulting from the dendrimer core are indicated in red and all of the sugar signals are in dark blue, as before. Due to the further removal of the non-reducing sialic acid from the oxime linkage, the non-equivalent proton signals are noted as H_9ax_ and H_9eq_, and H_16a_ and H_16b_, rather than with the *E*- and *Z*- notations, as these signals are always non-equivalent for sialic acid/oligosialic acid, and they are deemed too far removed from the oxime for the different isomeric forms of the linkage to have any significant effect. Additionally, there are two clear singlet signals for the *N*-acetyl groups at ∼2.1–2.0 ppm, arising from H_4_ and H_12_. Finally, the COSY reveals the approximate composition of the *E*- vs the *Z*-isomeric forms through the two H_m_ signals located at ∼4.2 and 4.1 ppm of 65 and 35%, respectively. The signals for H_m_ have become more broadened, not surprisingly, compared to that of compound **11**, due to them being moved further toward the interior of the molecule with the addition of another sialic acid on the periphery of the glycodendrimer. The connectivity of H_9_–H_16_ to C_27_–C_37_ can now be surmised using the HSQC for compound **12**. Here, we were again assisted by the phase sensitivity of the contours of the HSQC, where the dark blue signals represent carbons bearing an odd number of hydrogens (CH, CH_3_) and the light green signals originating from carbons bearing an even number of hydrogens (CH_2_). Similar to compound **11**, for compound **12**, the sugars consist mostly of signals arising from carbons bearing an odd number of hydrogens, whereas the core peaks all arise from CH_2_ signals, meaning that all of the dark blue signals come from the sugars alone. The only sugar signals resulting from CH_2_ groups arise from H_1_–C_18_ and H_8_–C_26_ for the reducing sugar, and the new signals from the non-reducing sugar, H_9_–C_29_ and H_16_–C_37_. Looking first at the CH and CH_3_ signals arising from the sialic acids for compound **12**, a total of 12 signals were anticipated, but 13 were visualized in the HSQC. The additional signal arises from the splitting of H_3_ into H_3E_ and H_3Z_, as was discussed for compound **11**. For the sugar CH_2_ signals, we were able to clearly visualize the H_9ax_–C_29_ signal at 1.8 ppm (F2), but the H_9eq_–C_29_ signal at ∼2.75 ppm (F2) was found to overlap with H_k_ and H_1E_. H_9_ was found to be attached to C_29_, which resulted in a single signal at ∼62 ppm (F1). For the second CH_2_ signal, the H_16_–C_37_, H_16_ was found to split into two separate signals, H_16a_ (∼3.8–3.9 ppm, F2) and H_16b_ (∼3.6–3.7 ppm, F2) due to the proximity to the H_15_ stereocenter, while a single signal for C_37_ was apparent in the F1 dimension at ∼66 ppm.

For compounds **13–14**, the same strategy was utilized for the assignments of both the protons and carbons present. The challenge with the assignments for these molecules mostly centered on the severe overlapping of the sugar signals, coupled with the further peak broadening as the molecule increased in size. These spectra (^1^H, ^13^C, COSY, and HSQC) can be viewed in the Supporting Information and were assigned to the best of our capabilities.

## Conclusions

We have demonstrated the efficient synthesis of large octavalent poly(ether amidoamine) glycodendrimers terminating in oxime-linked monomer-tetramers of sialic acid. Good to excellent yields (58–100%) resulted in the full functionalization of the aminooxy-terminated dendrimer core with the sialic acid chains through the use of a microwave-mediated oxime coupling, performed in aqueous solution and completed in just a few hours. Once purified, an extensive and systematic approach was taken with 1D and 2D NMR experiments to attempt to fully assign the proton and carbon signals for these macromolecules. Starting with the smaller and working our way up to the larger molecules, while also working from the inside to the outside of the molecules, allowed us to use the information from the smaller pieces and apply it to the larger, more complex molecules to enable the proton and carbon signal assignments. The ultimate goal of this work is to explore the potential of sialic acid-terminated glycodendrimers as broad-spectrum topical anti-viral agents.

## Supporting Information Available

All NMR spectra (1D: ^1^H and ^13^C and 2D: COSY and HSQC) for all purified synthetic compounds; summarized carbon peak assignments; IR and HR–ESI–MS spectra for compounds analyzed by these methods; and all final glycodendrimer FPLC elution profiles as well as SEC–MALS chromatograms (PDF)
