# Supplementary material for: Synthesis and Nuclear Magnetic Resonance Structural Evaluation of Oxime-Linked Oligosialic Acid-Based Glycodendrimers
Source: Biomacromolecules. 2023 Mar 29;24(4):1901–11. doi: 10.1021/acs.biomac.3c00105 (PMC10091409; doi:10.1021/acs.biomac.3c00105)

# Supporting Information

# Synthesis and Nuclear Magnetic Resonance Structural Evaluation of Oxime-Linked Oligosialic Acid-Based Glycodendrimers

James P. Cerney, Aleksey Raskovalov, Monica Nasseri, Madeline D. Silva, Katherine D. McReynolds*

Department of Chemistry, California State University, Sacramento
6000 J Street
Sacramento, CA 95819-6057
kdmcr@csus.edu
1-916-278-6551 (phone)
1-916-278-4986 (fax)

**Table of Contents:**

| | |
|---|---|
| $^{1}H$ NMR spectra: | pages 2-13 |
| $^{13}C$ NMR spectra: | pages 14-26 |
| COSY spectra: | pages 27-38 |
| HSQC spectra: | pages 39-48 |
| Tables of $^{13}C$ peak assignments | pages 49-50 |
| IR spectra: | page 51-52 |
| ESI-MS spectra: | pages 53-57 |
| FPLC chromatograms: | pages 58-61 |
| SEC-MALS chromatograms: | pages 62-63 |

**Figure S1: $^{1}$H of Compound 1 in $D_2O$.**

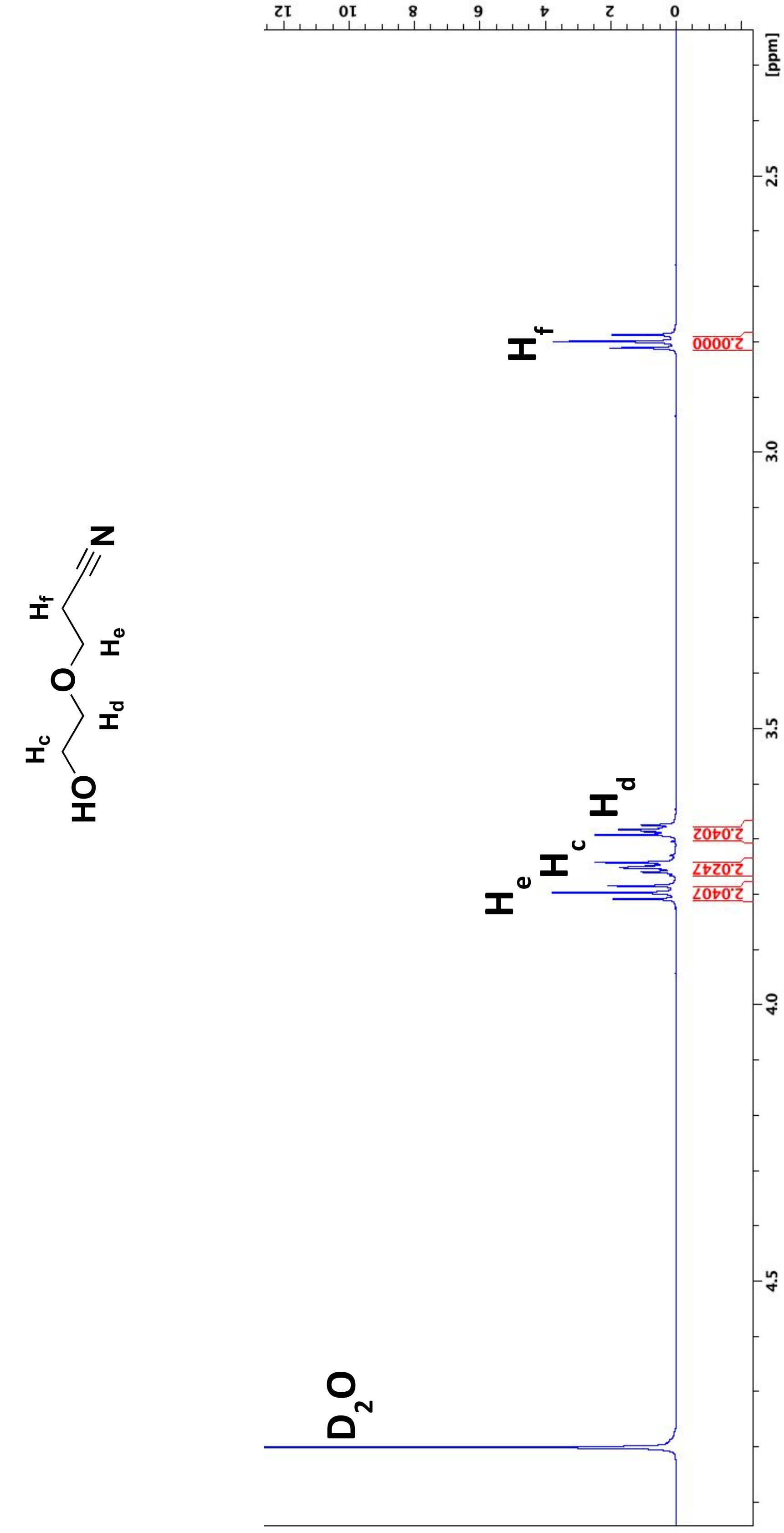

**Figure S2: $^{1}H$ of Compound 2 in $CDCl_3$.**

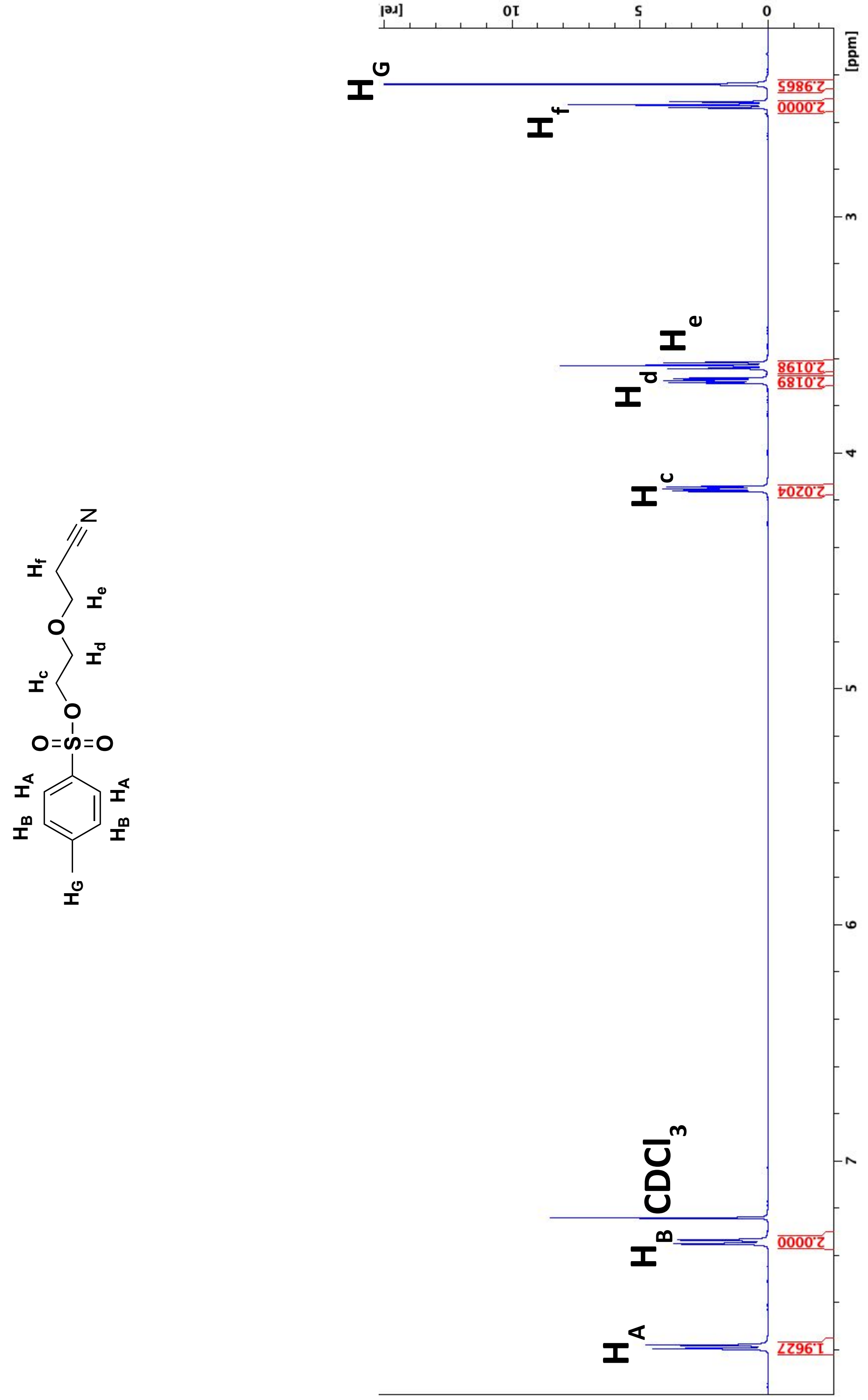

**Figure S3: $^{1}$H of Compound 3 in $D_2O$.**

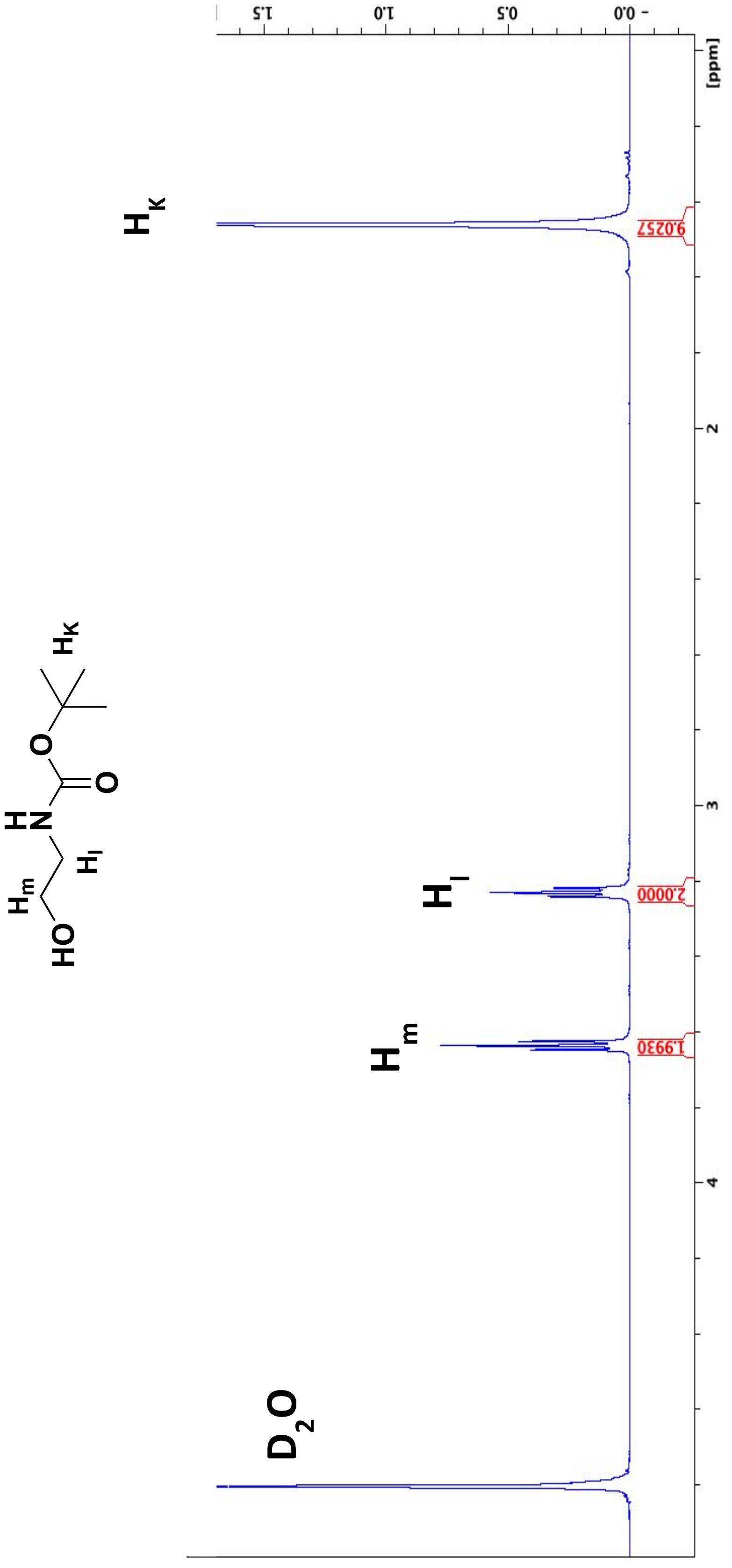

**Figure S4: $^{1}H$ of Compound 5 in $D_2O$.**

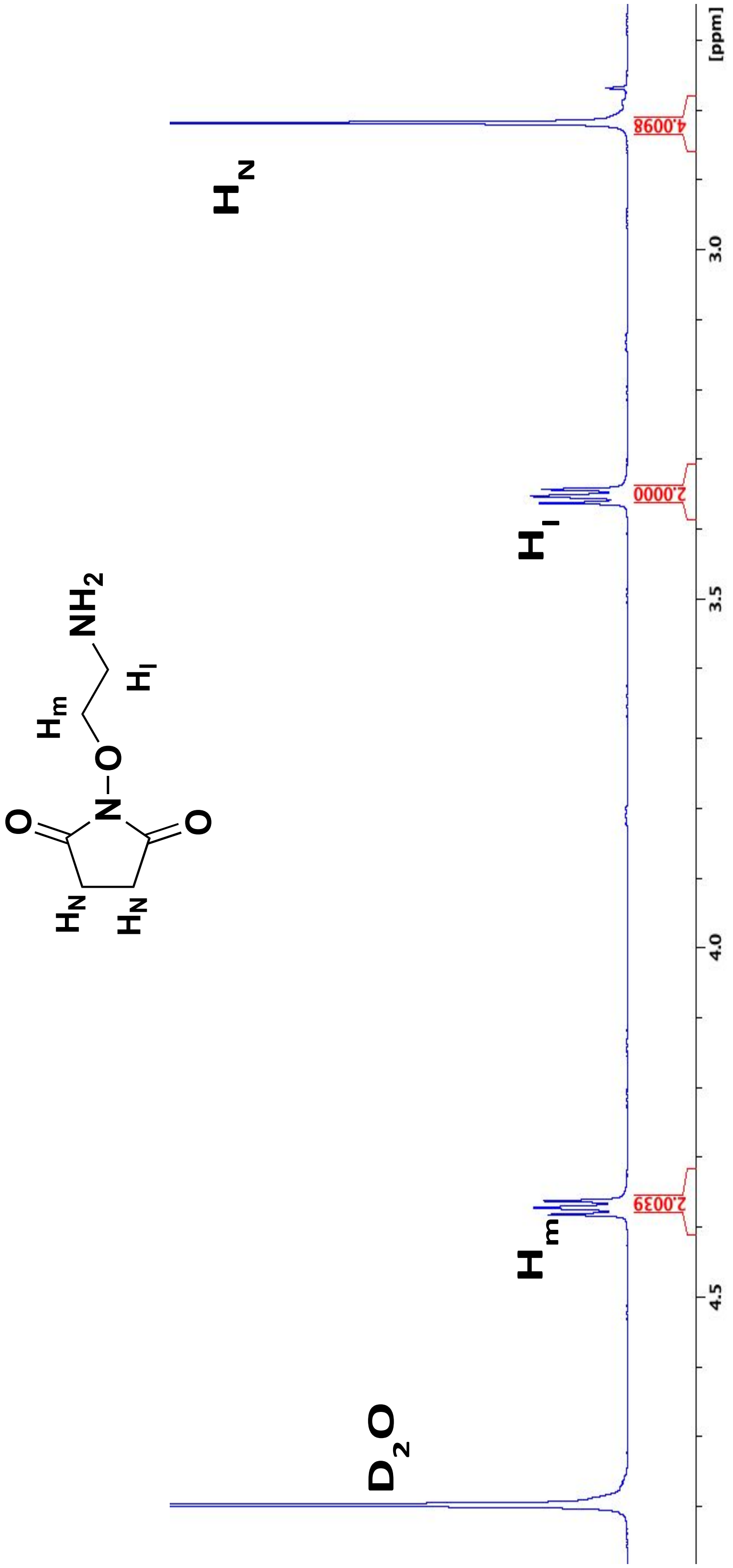

Figure S5: $^{1}$H of Compound 6 in $D_2O$.

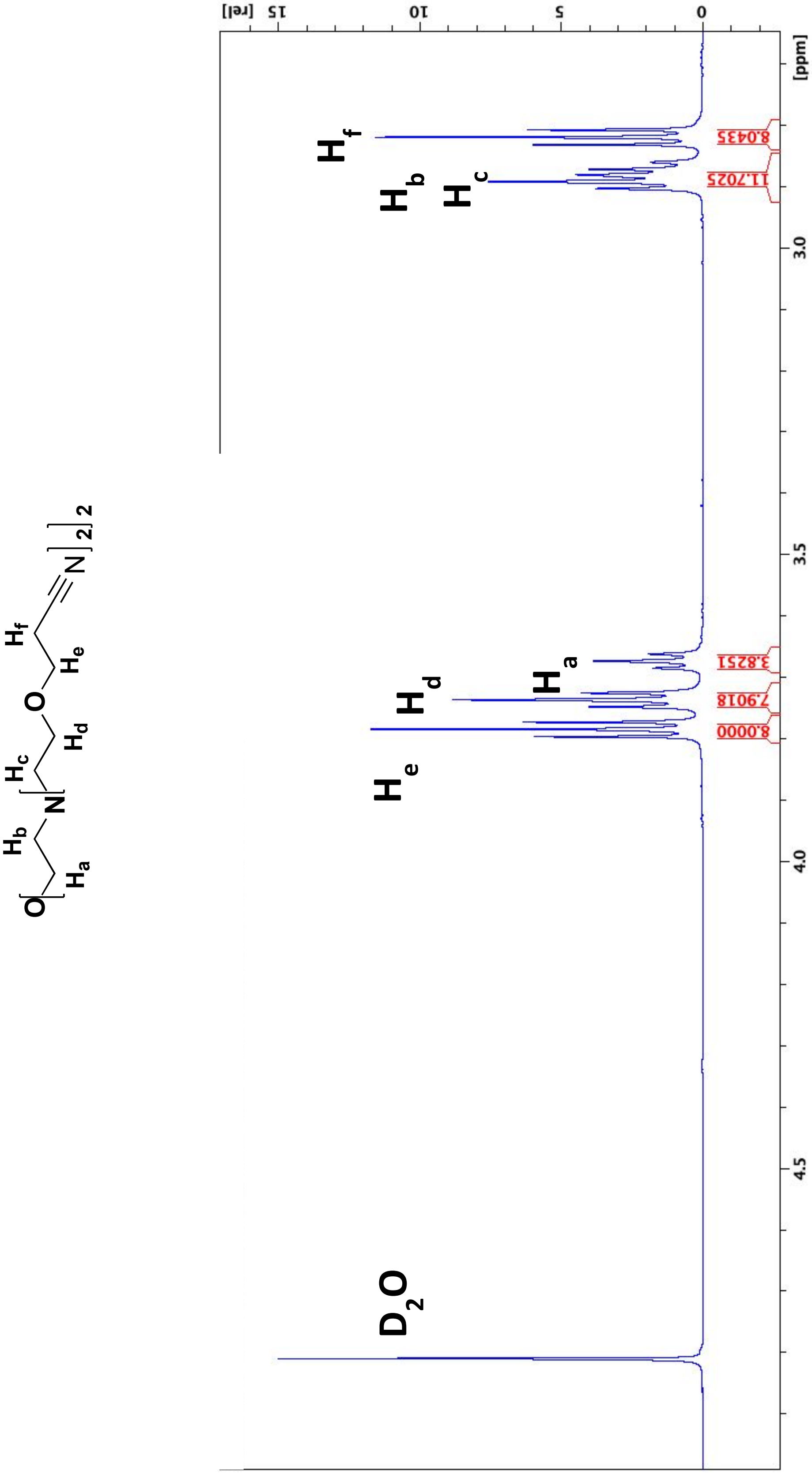

**Figure S6: $^{1}H$ of Compound 8 in $D_2O$.**

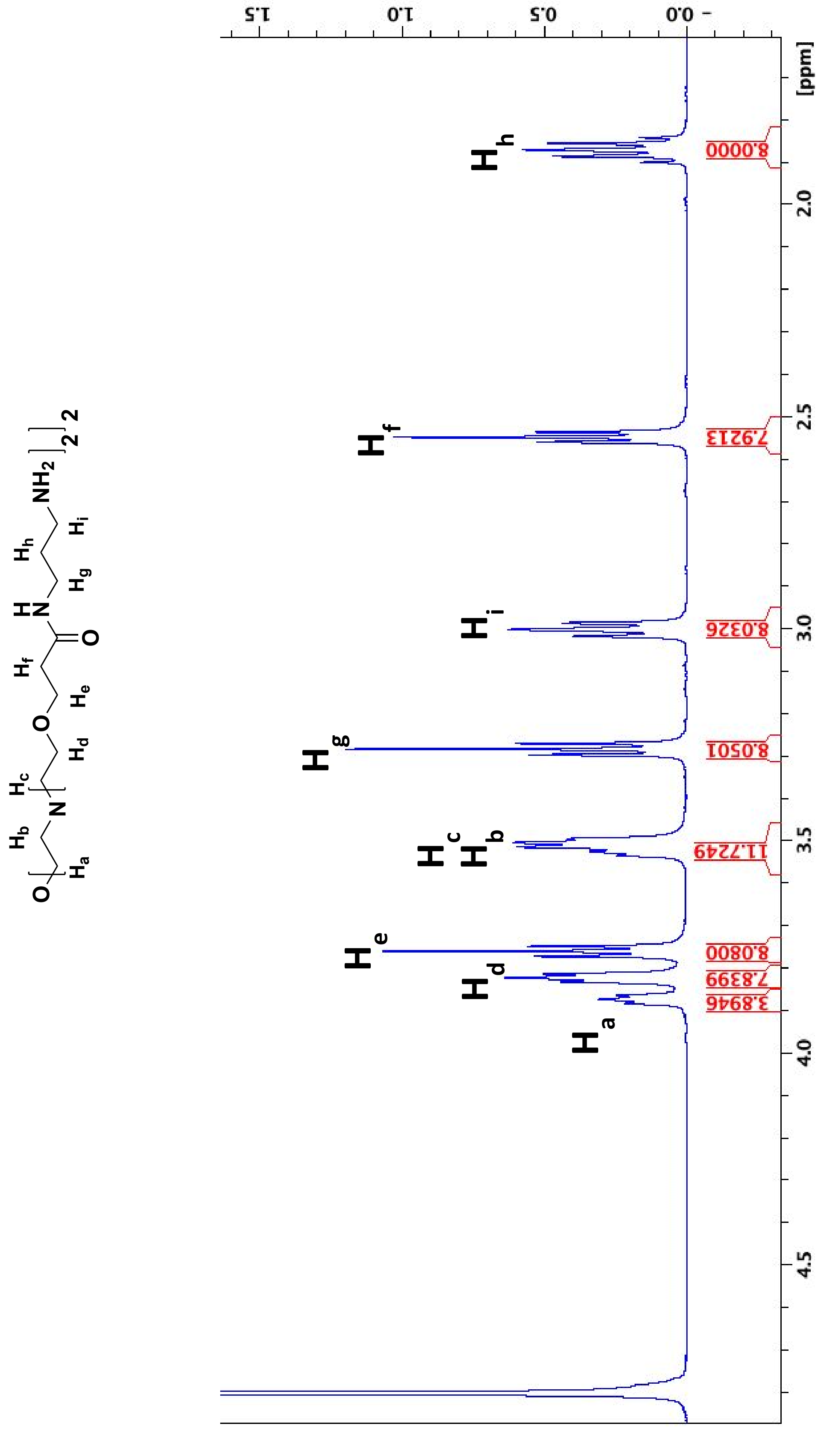

**Figure S7: $^{1}H$ of Compound 9 in $D_2O$.**

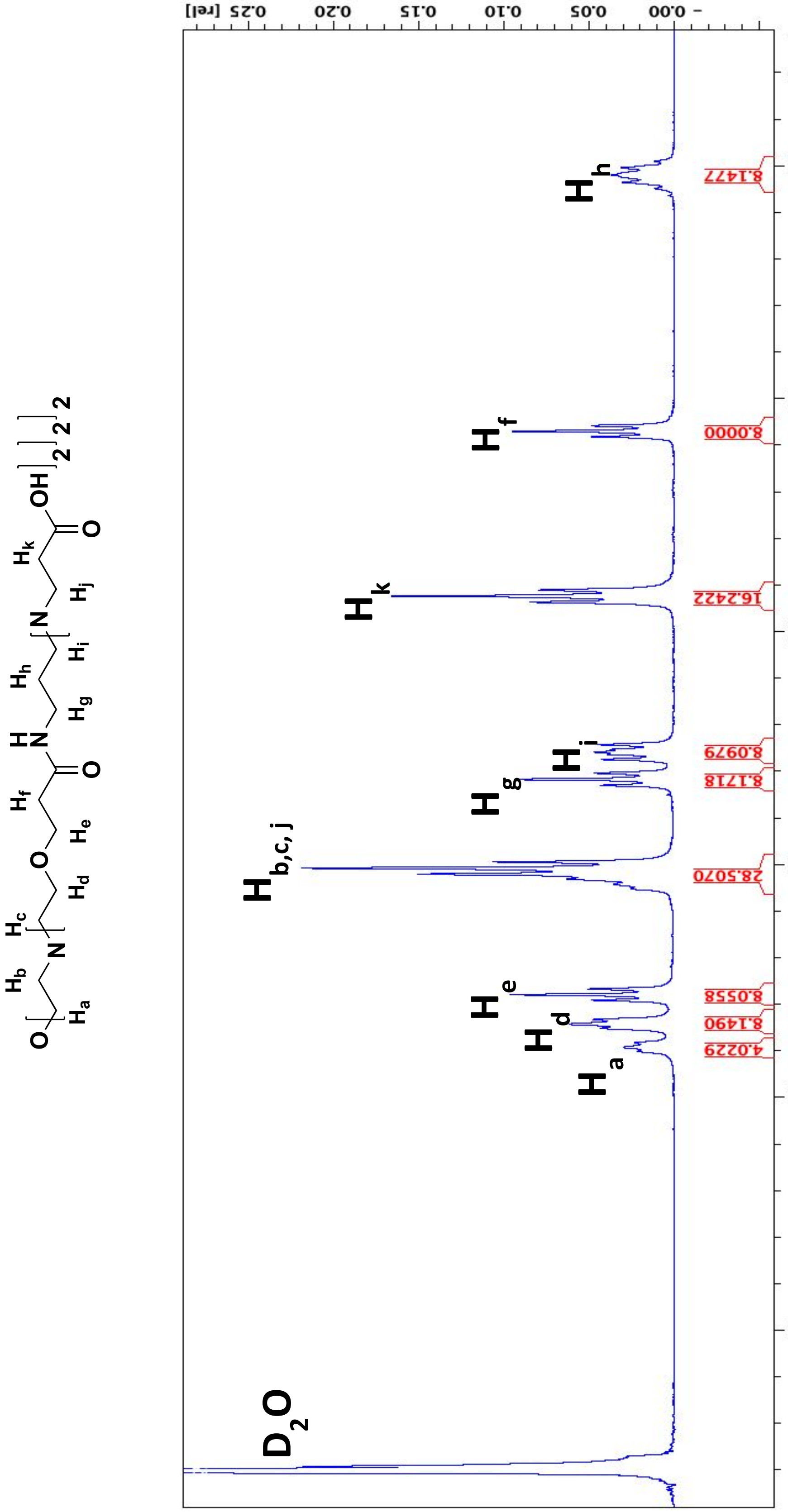

**Figure S8: $^{1}$H of Compound 10 in $D_2O$.**

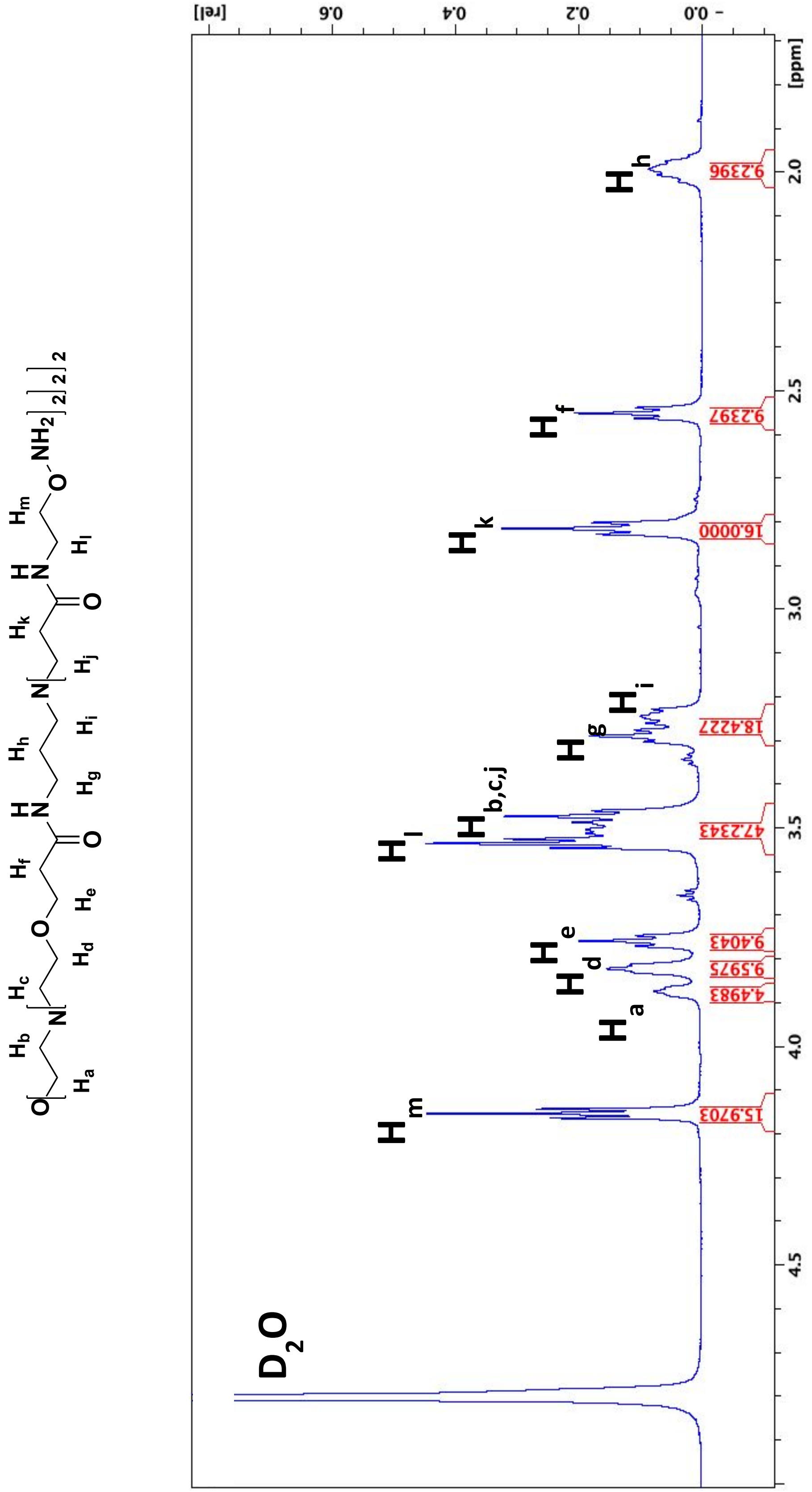

**Figure S9: $^{1}$H of Compound 11 in $D_2O$.**

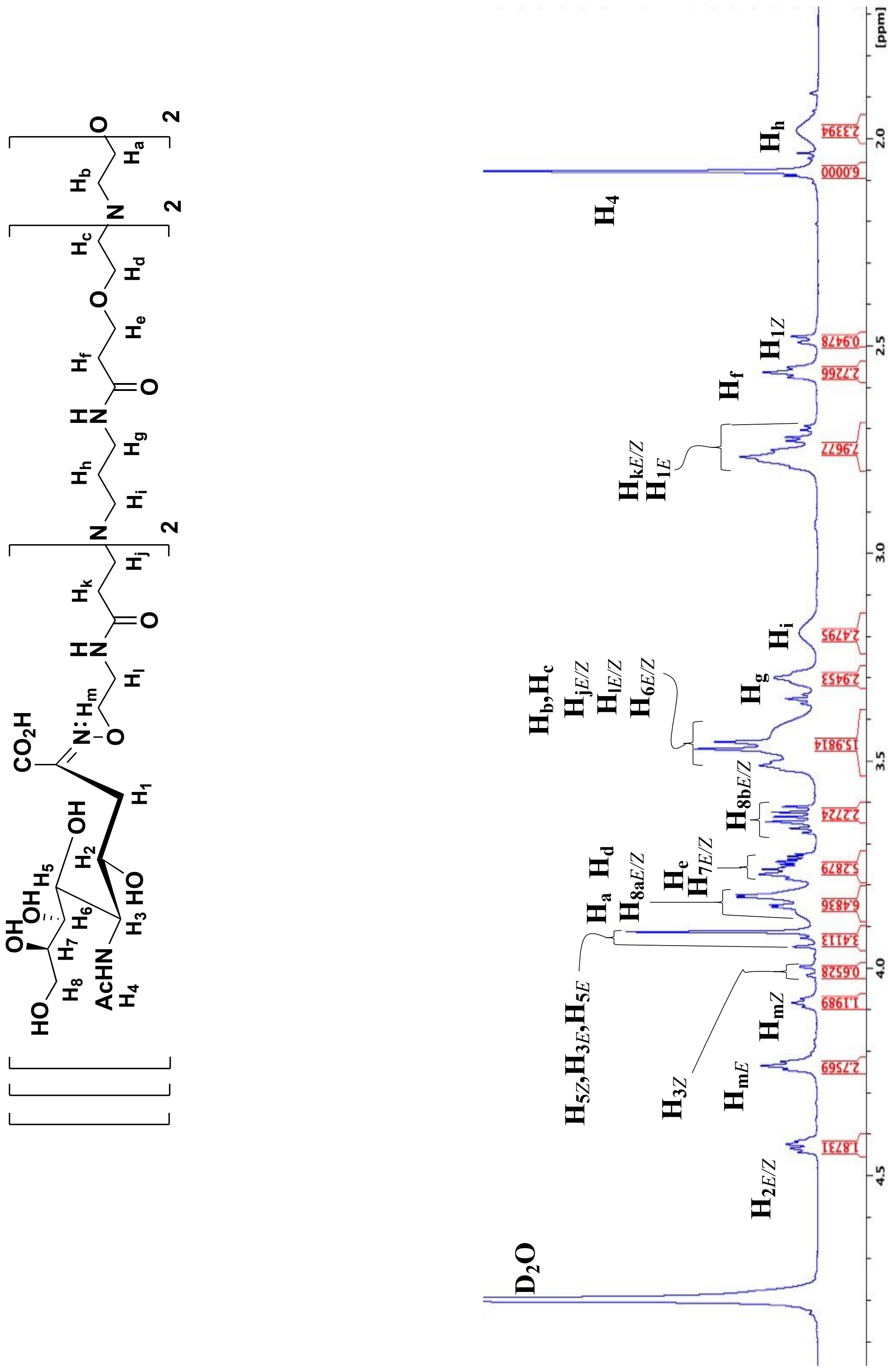

**Figure S10: $^{1}H$ of Compound 12 in $D_2O$.**

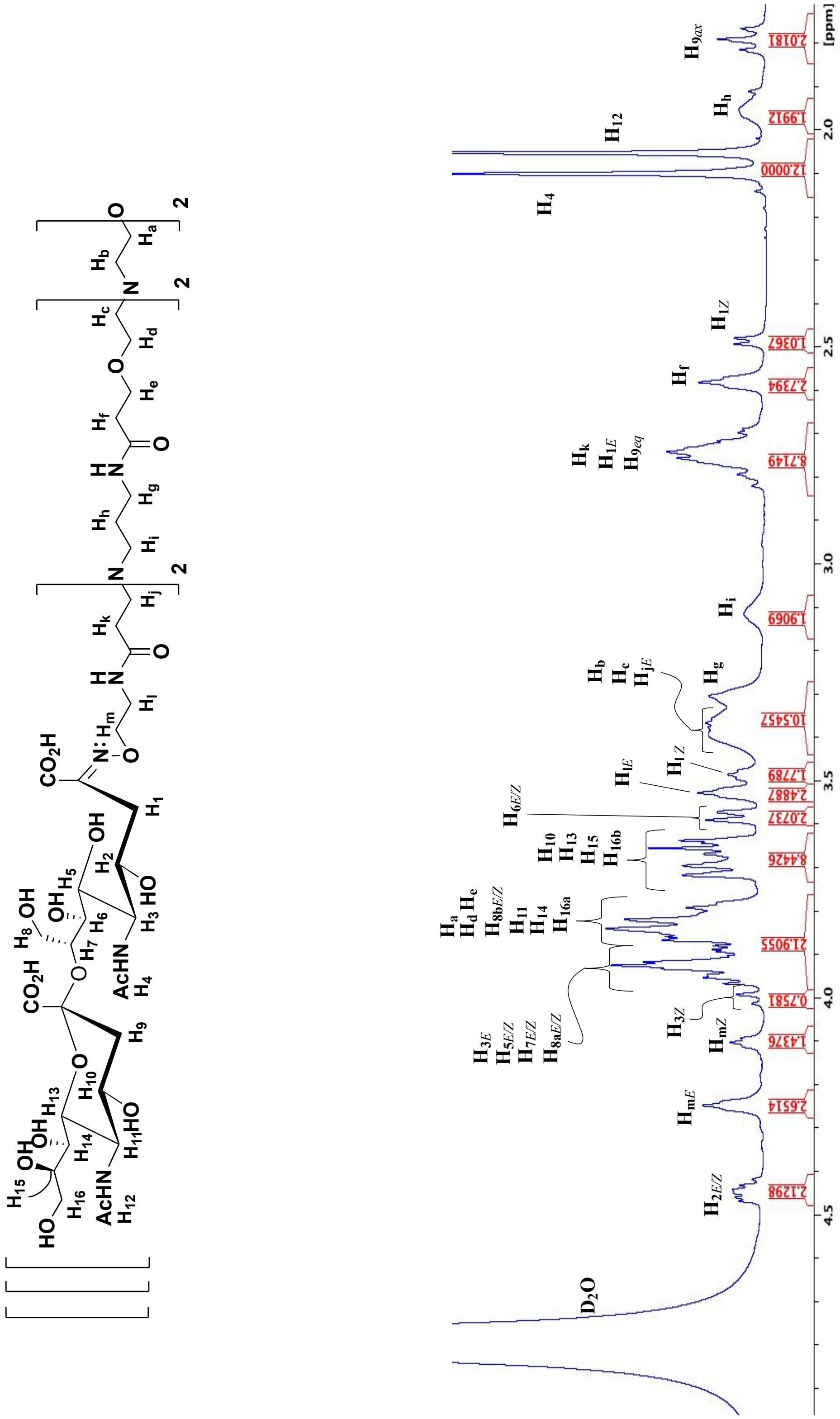

**Figure S11: $^{1}$H of Compound 13 in $D_2O$.**

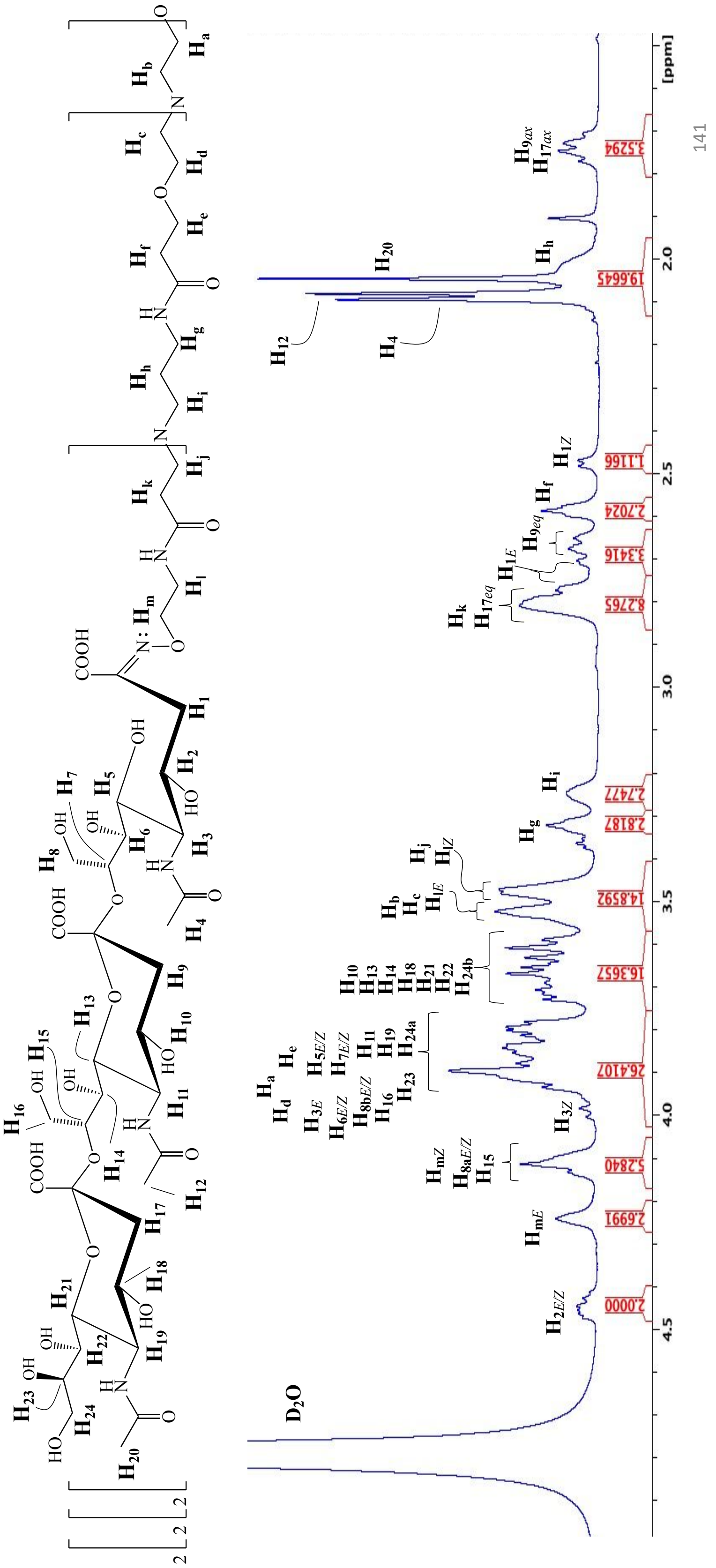

**Figure S12: $^{1}$H of Compound 14 in $D_2O$.**

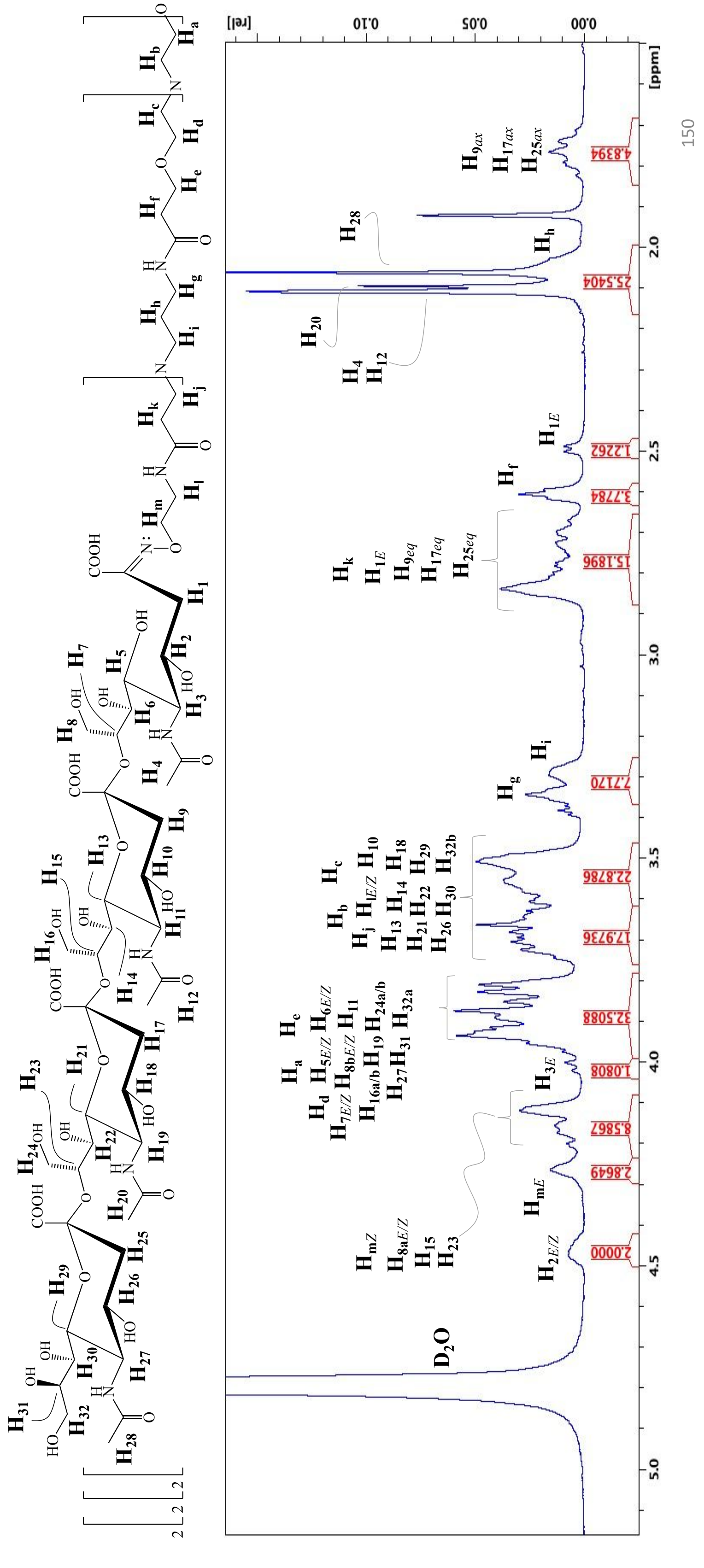

**Figure S13: $^{13}C$ of Compound 1 in $D_2O$ with an internal MeOH standard.**

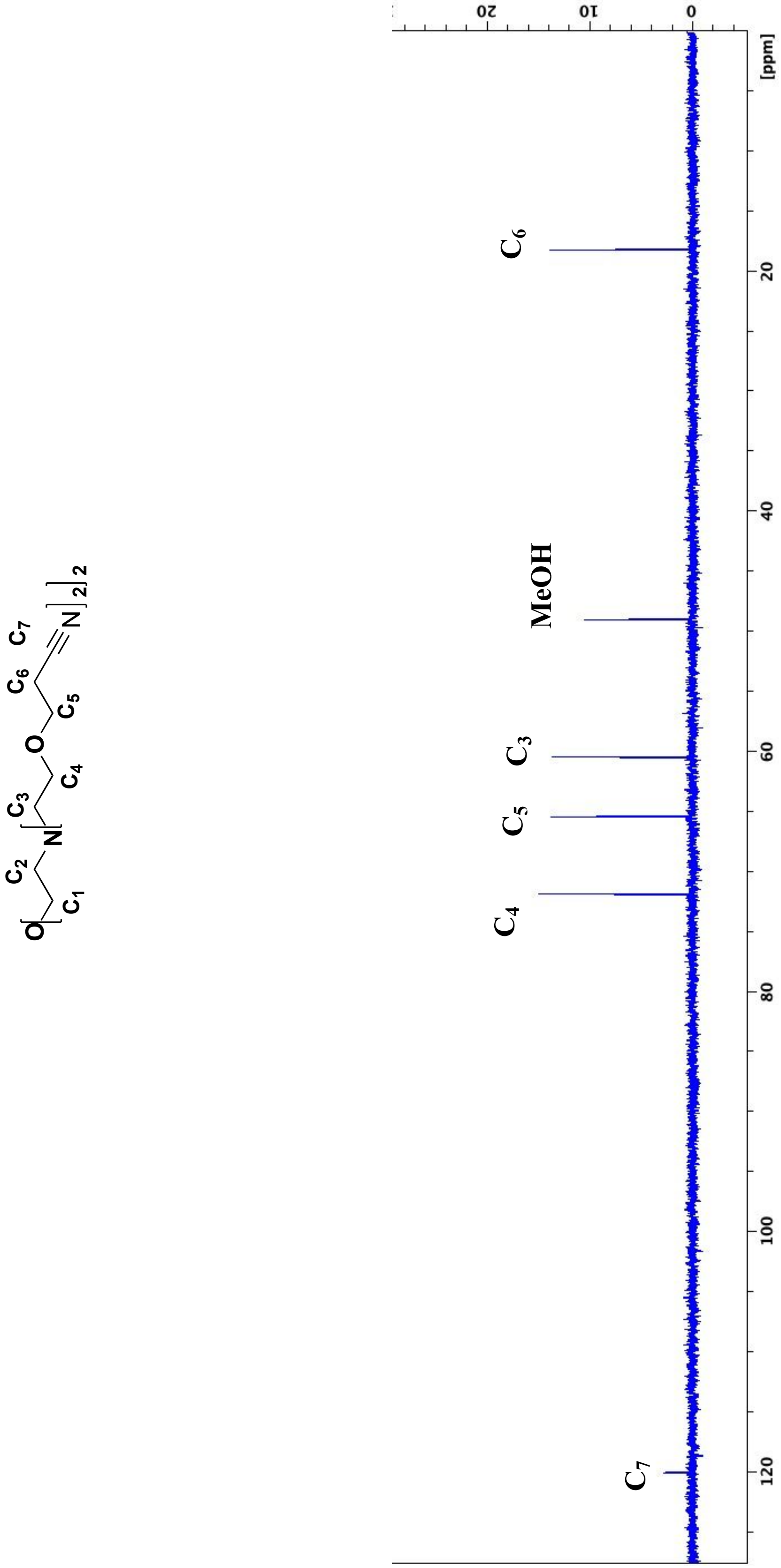

**Figure S14: $^{13}C$ of Compound 2 in $CDCl_3$.**

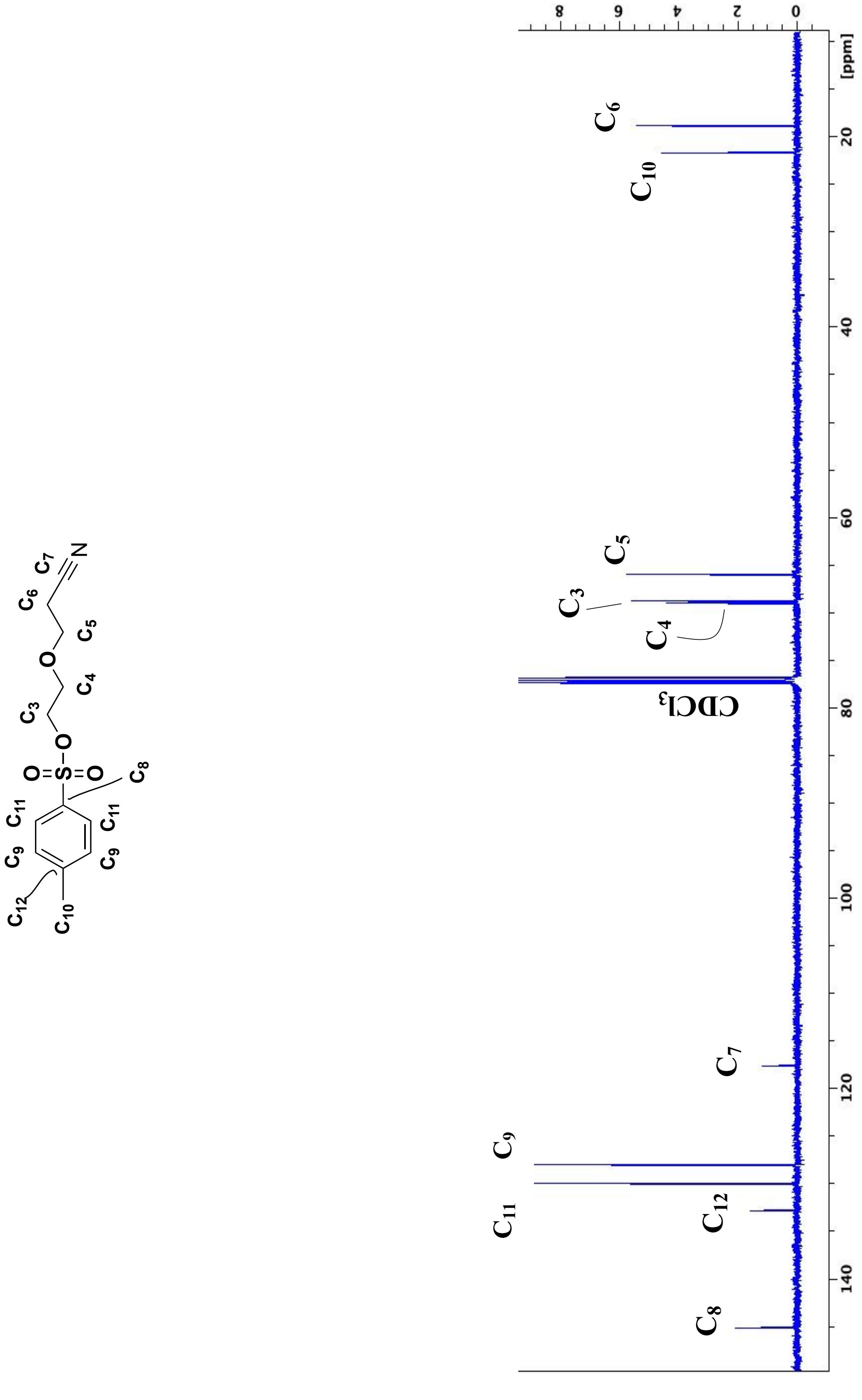

Figure S15: $^{13}$C of Compound 3 in $D_2O$ with an internal MeOH standard.

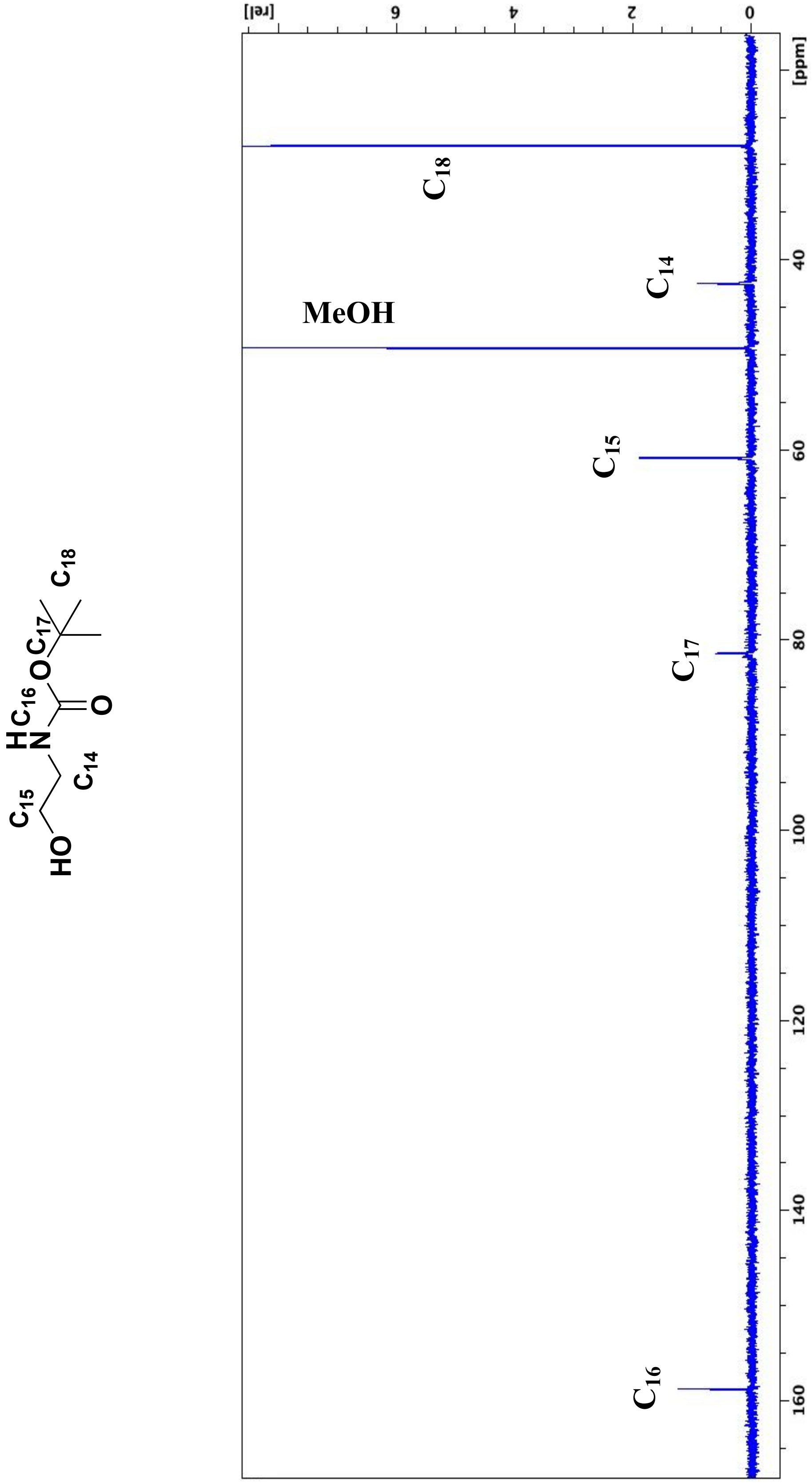

**Figure S16: $^{13}C$ of Compound 5 in $D_2O$ with an internal MeOH standard.**

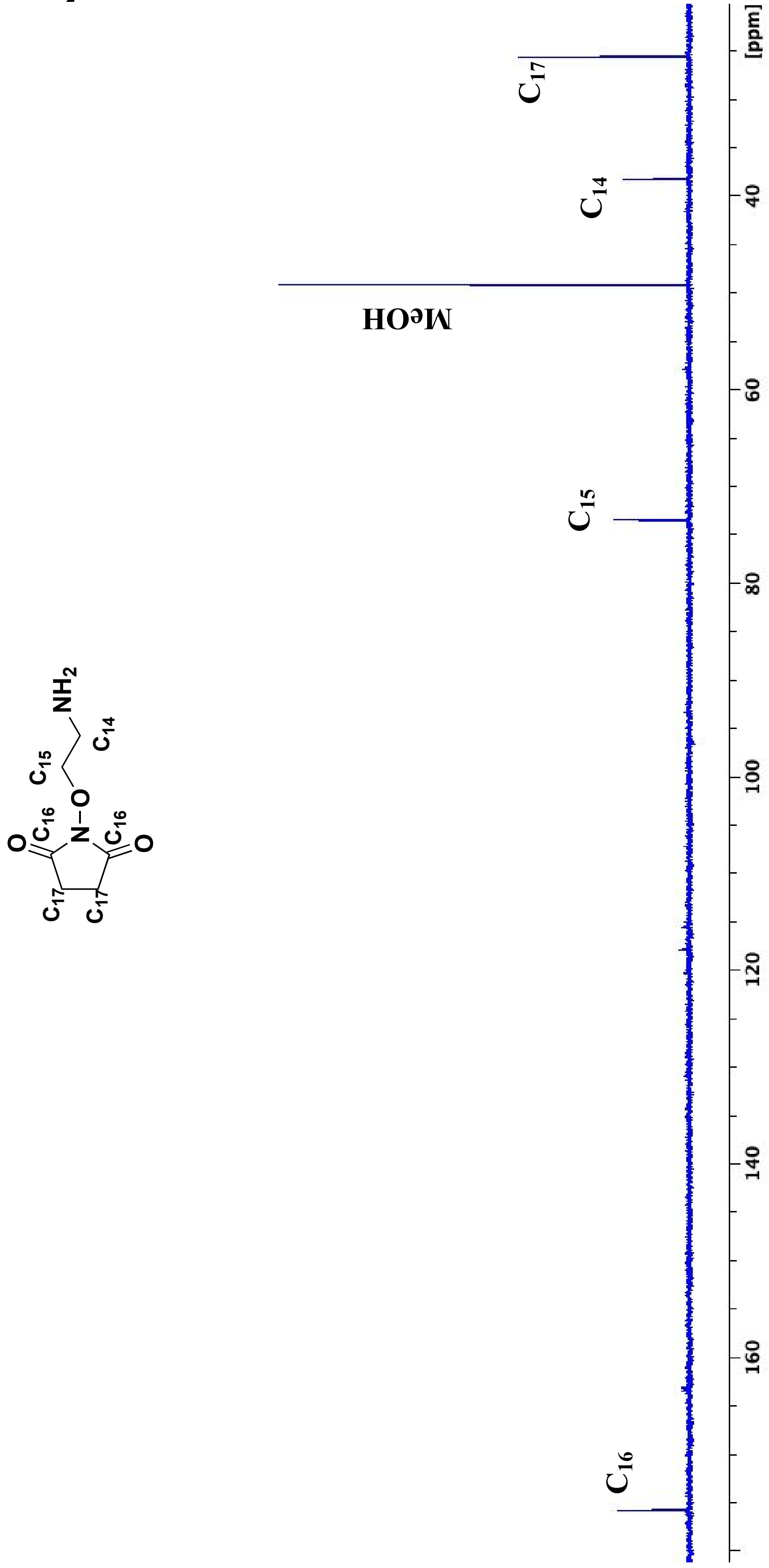

**Figure S17: $^{13}C$ of Compound 6 in $D_2O$ with an internal MeOH standard.**

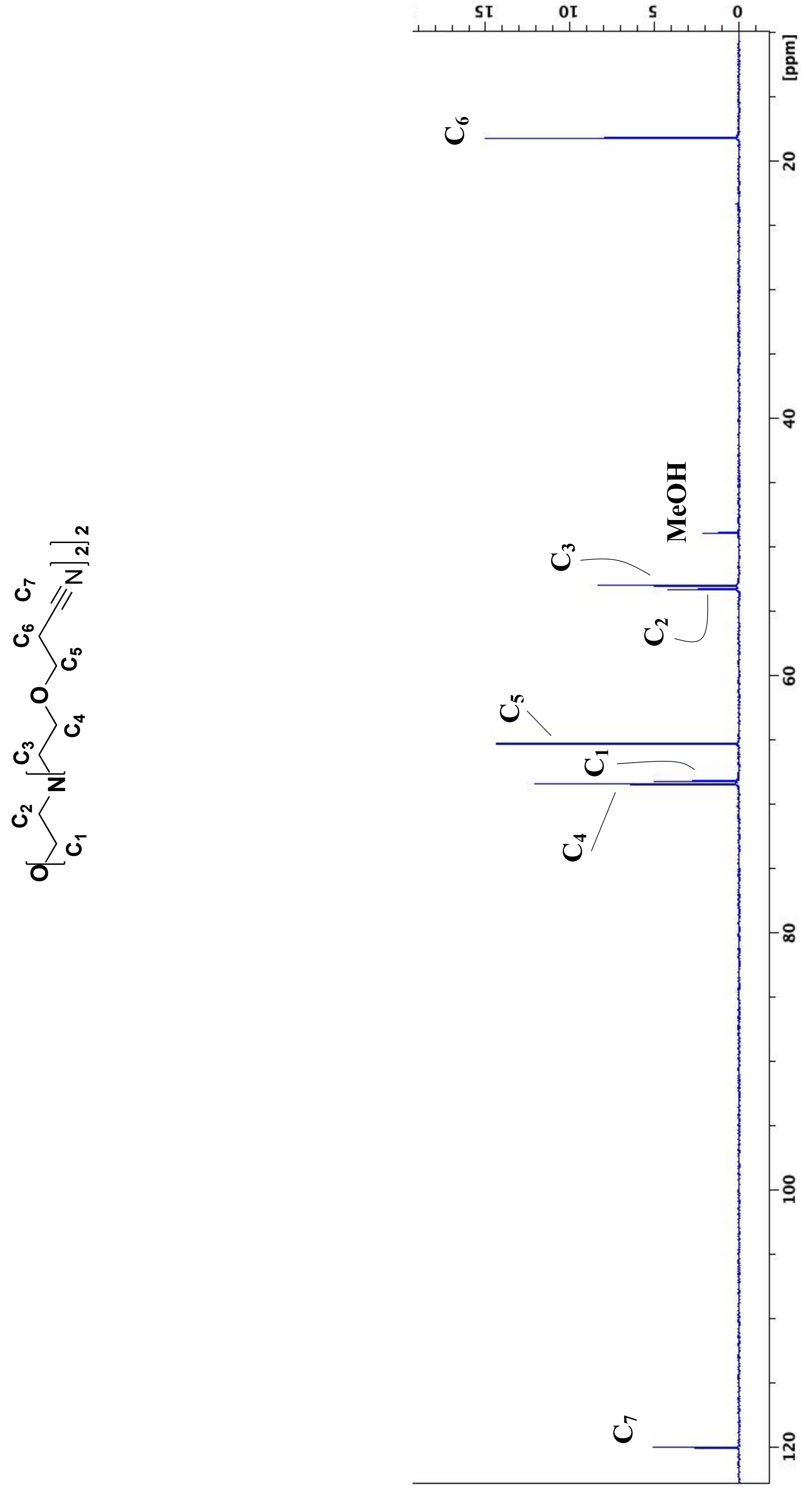

**Figure S18: $^{13}$C of Compound 8 in $D_2O$ with an internal MeOH standard.**

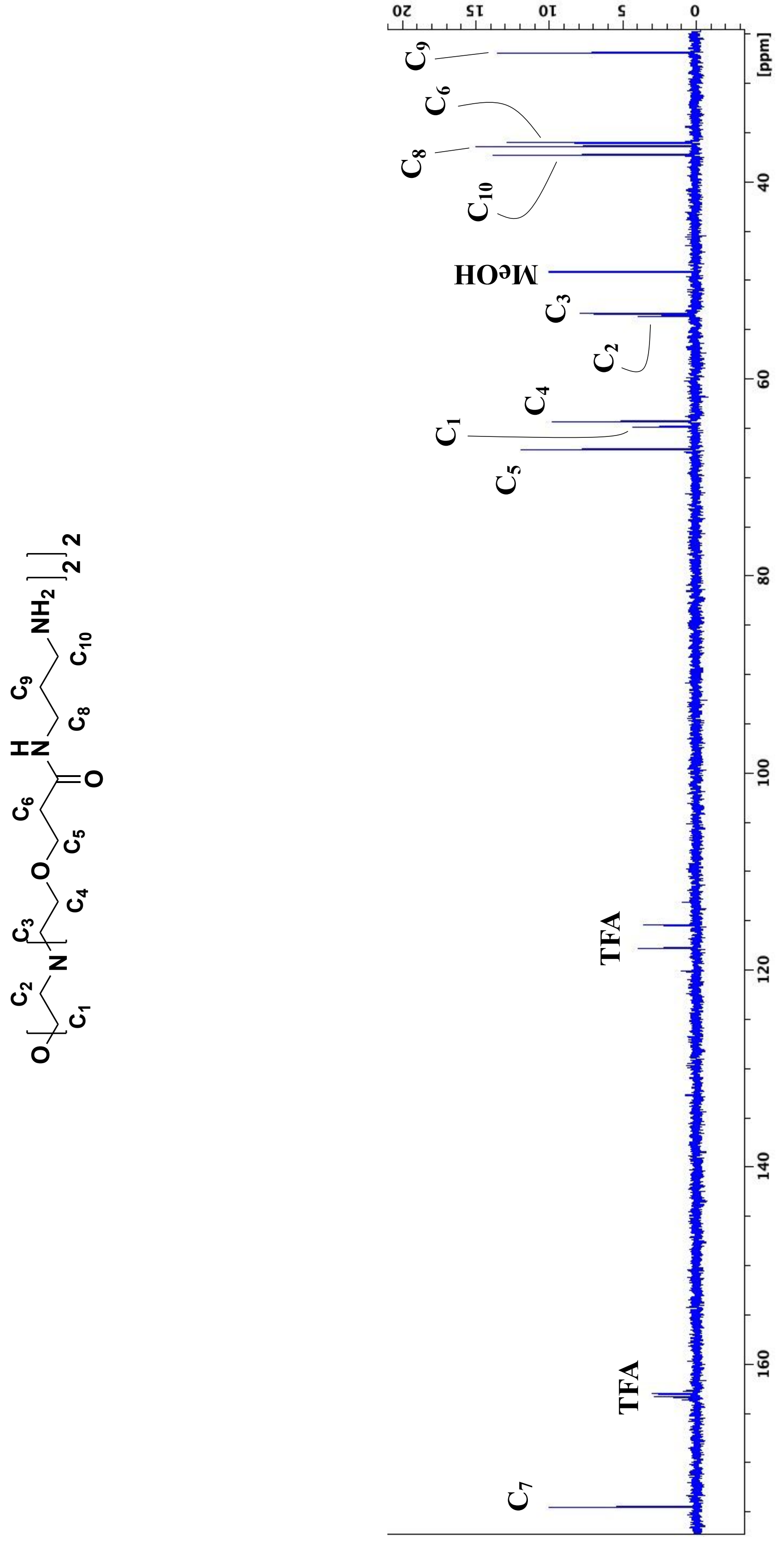

**Figure S19: $^{13}C$ of Compound 9 in $D_2O$ with an internal MeOH standard.**

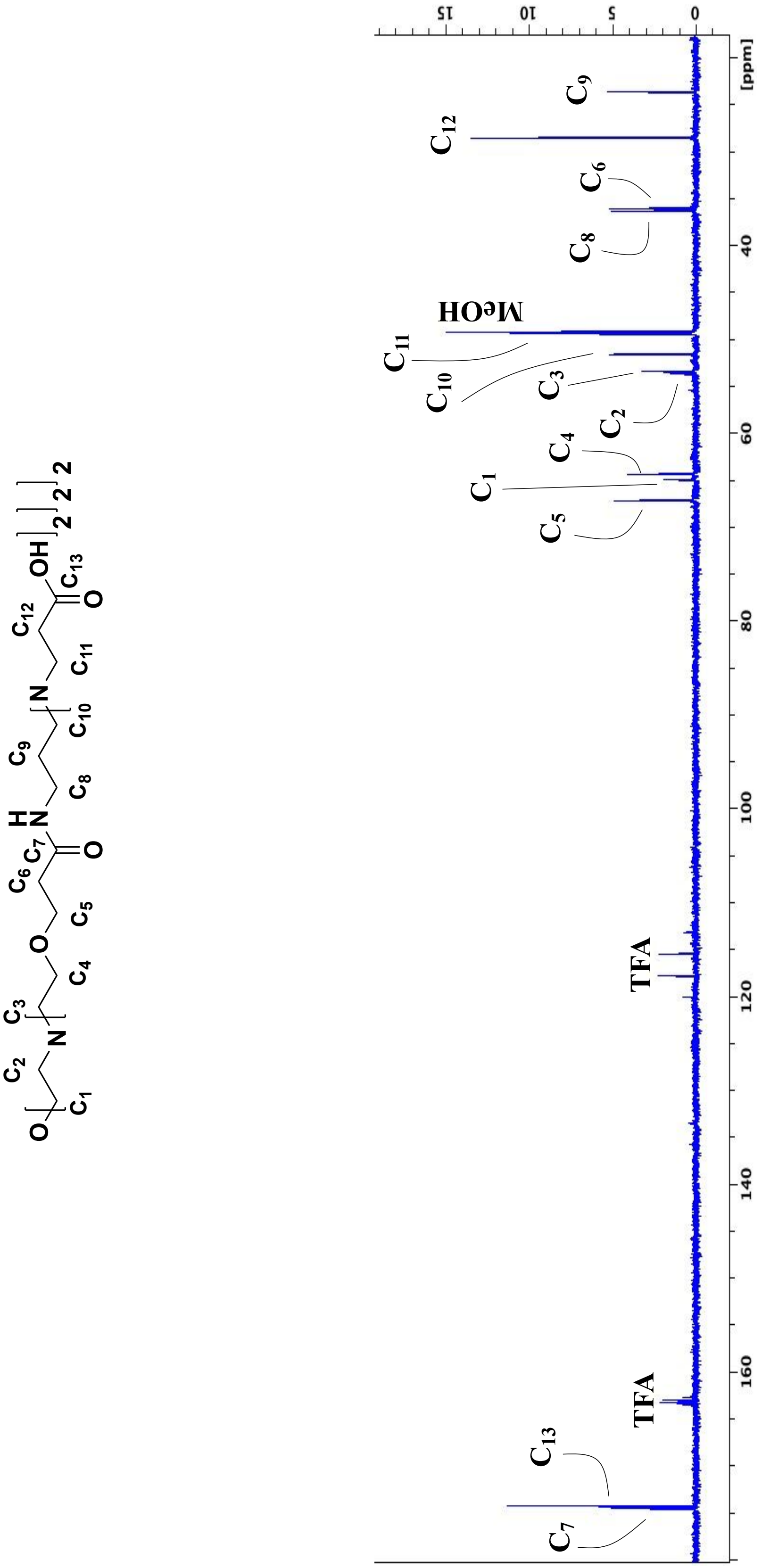

**Figure S20: $^{13}C$ of Compound 10 in $D_2O$ with an internal MeOH standard.**

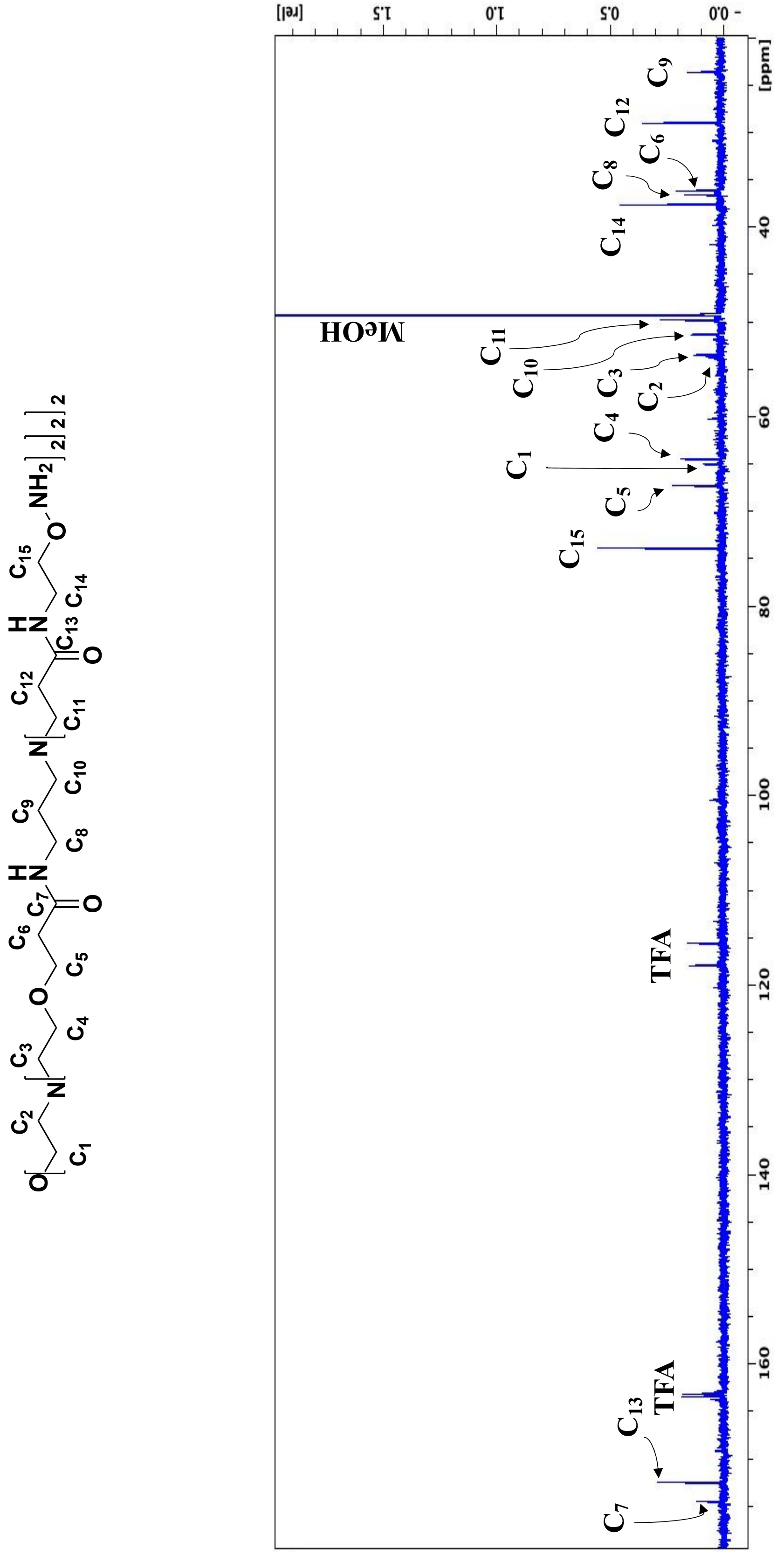

**Figure S21: $^{13}C$ of Compound 11 *E*-isomer peaks in $D_2O$ with an internal MeOH standard.**

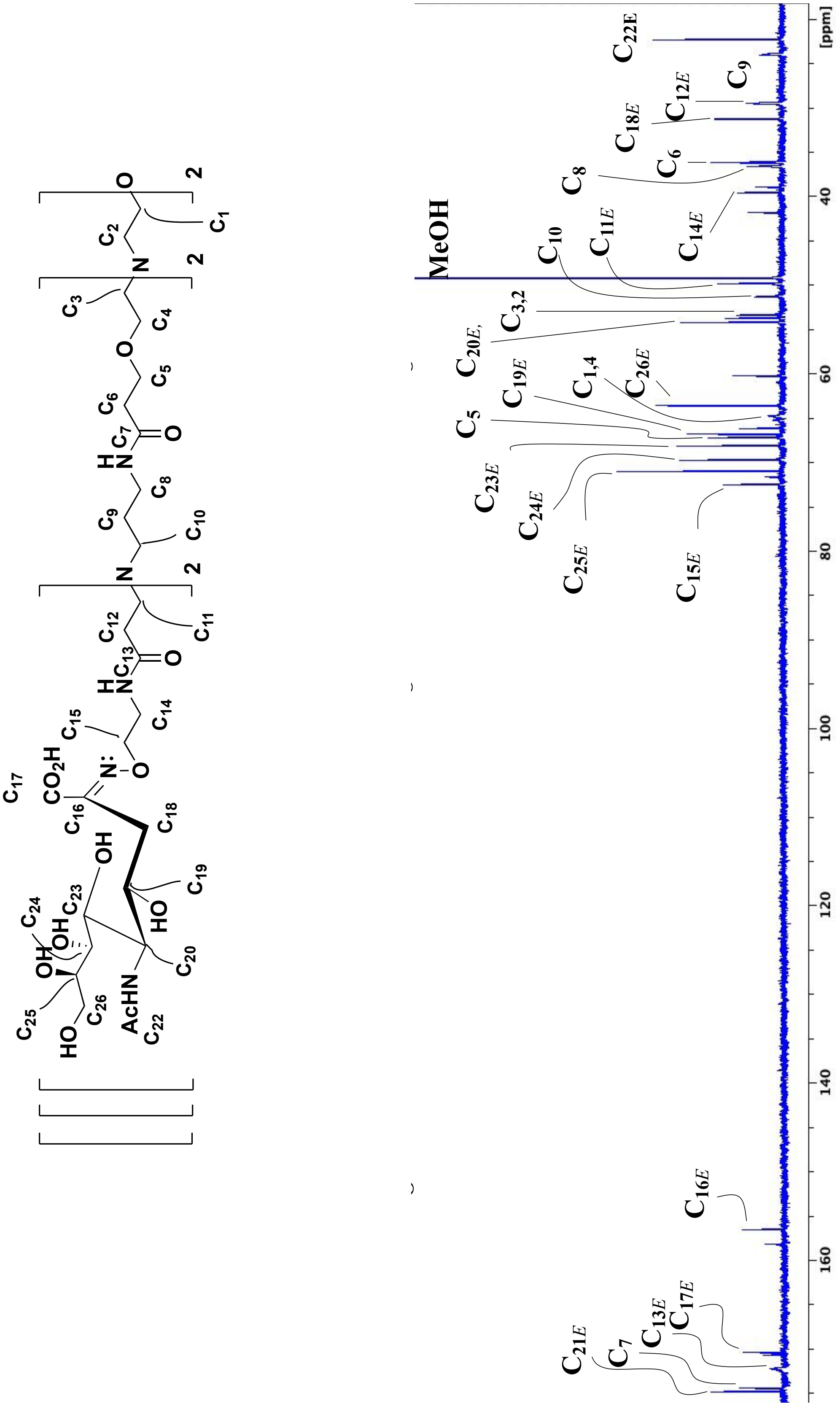

**Figure S22: $^{13}$C of Compound 11 *Z*-isomer peaks in $D_2O$ with an internal MeOH standard.**

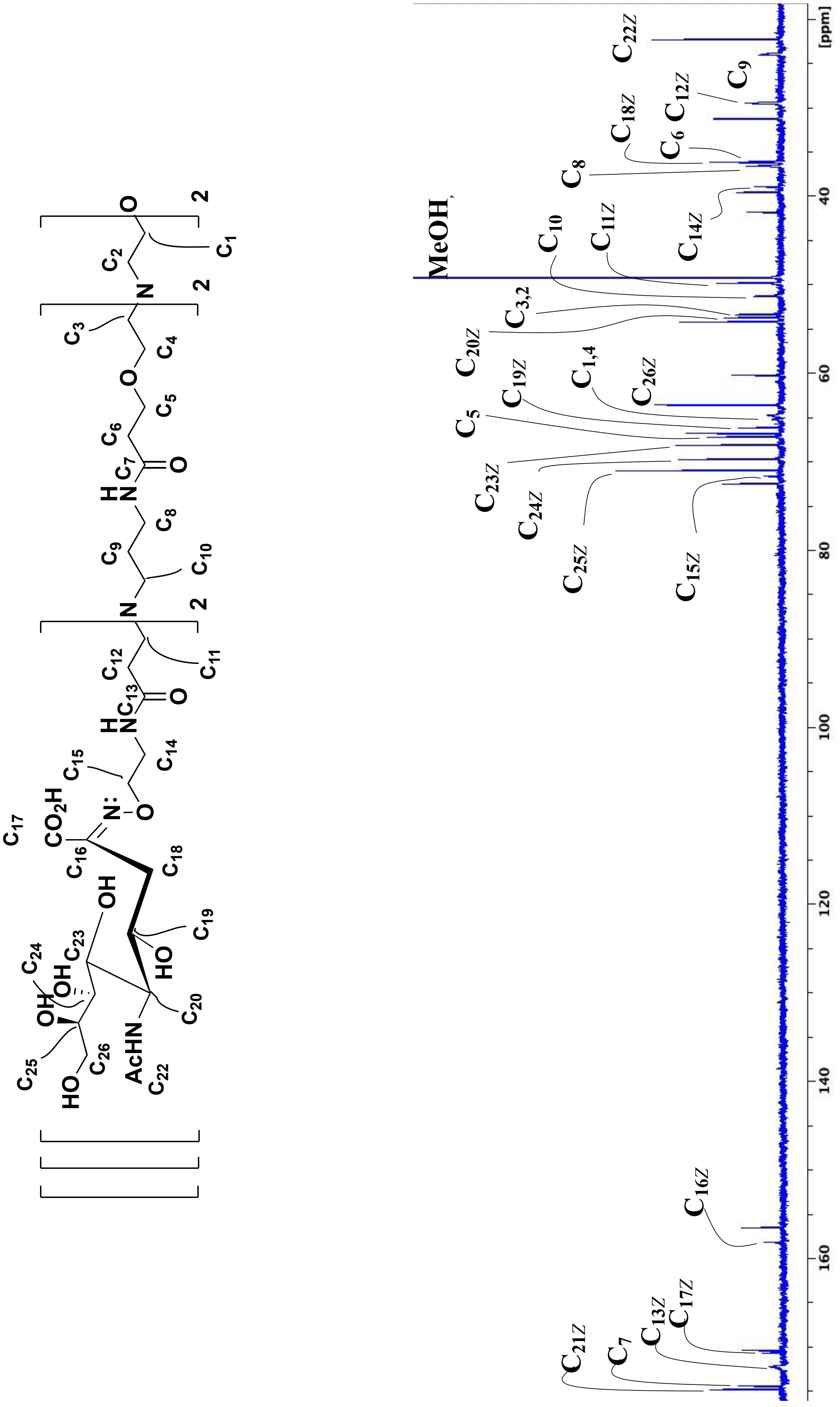

**Figure S23: $^{13}$C of Compound 12 in $D_2O$ with an internal MeOH standard.**

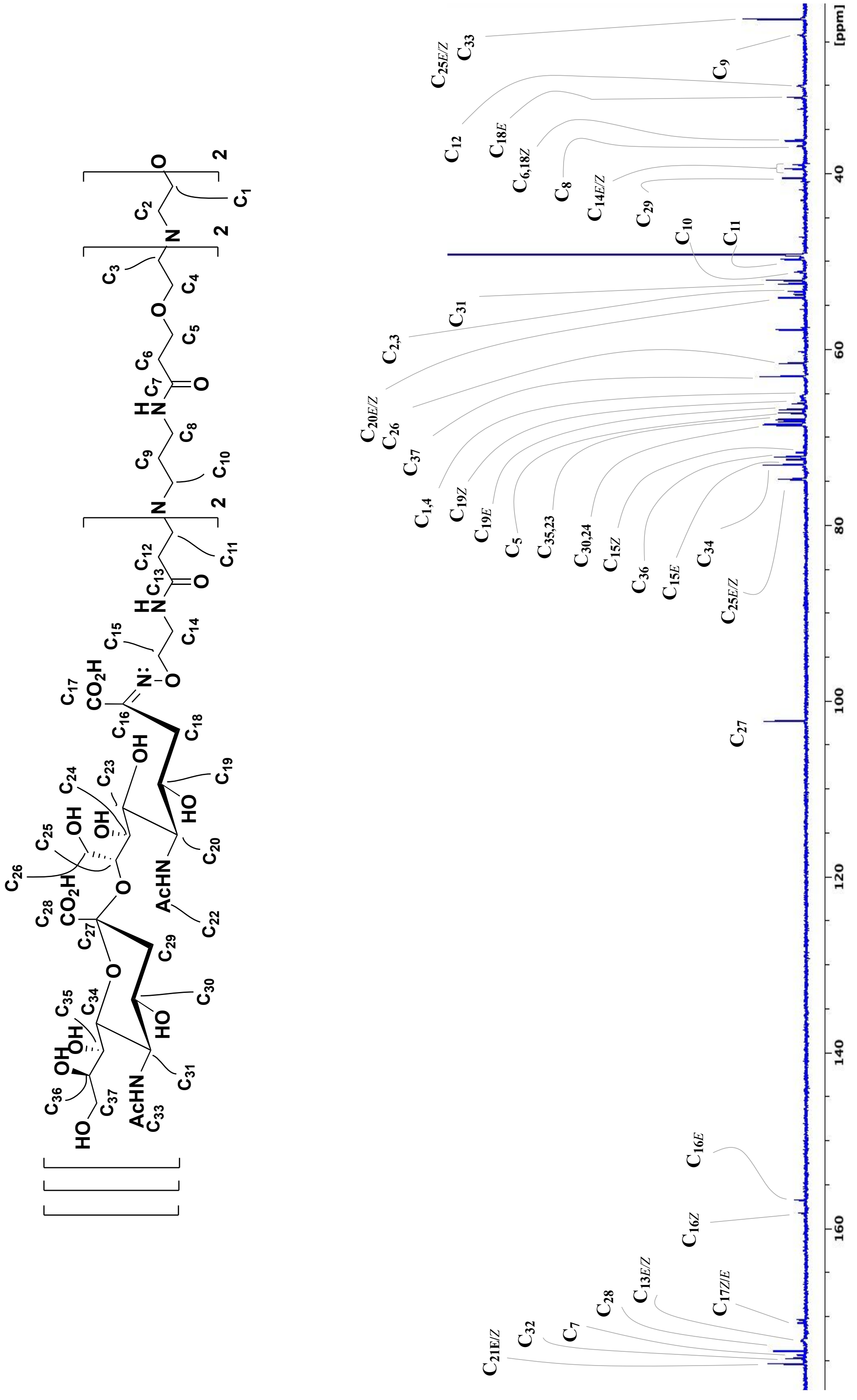

**Figure S24: $^{13}$C of Compound 13 in $D_2O$ with an internal MeOH standard.**

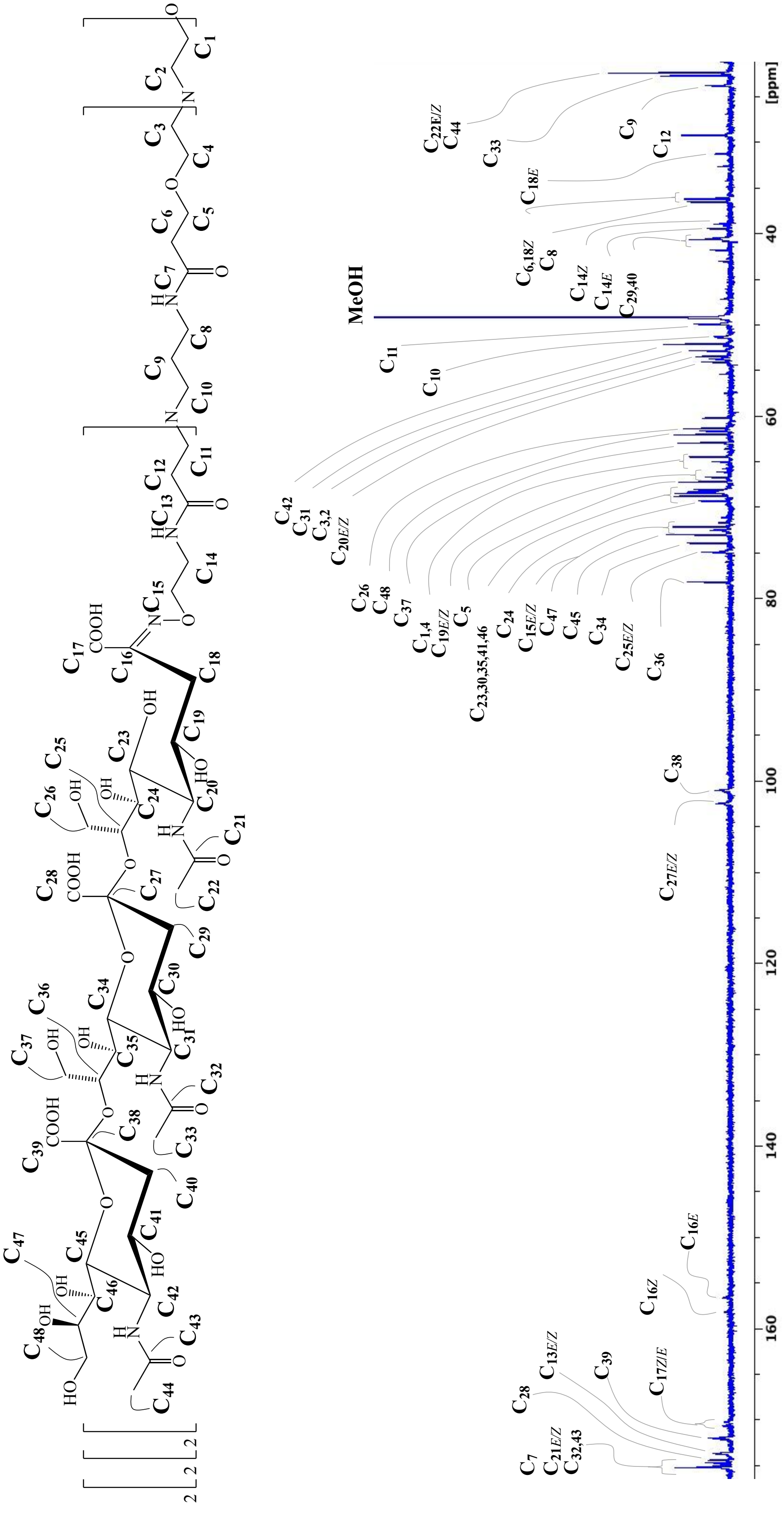

**Figure S25: $^{13}$C of Compound 14 in $D_2O$ with an internal MeOH standard.**

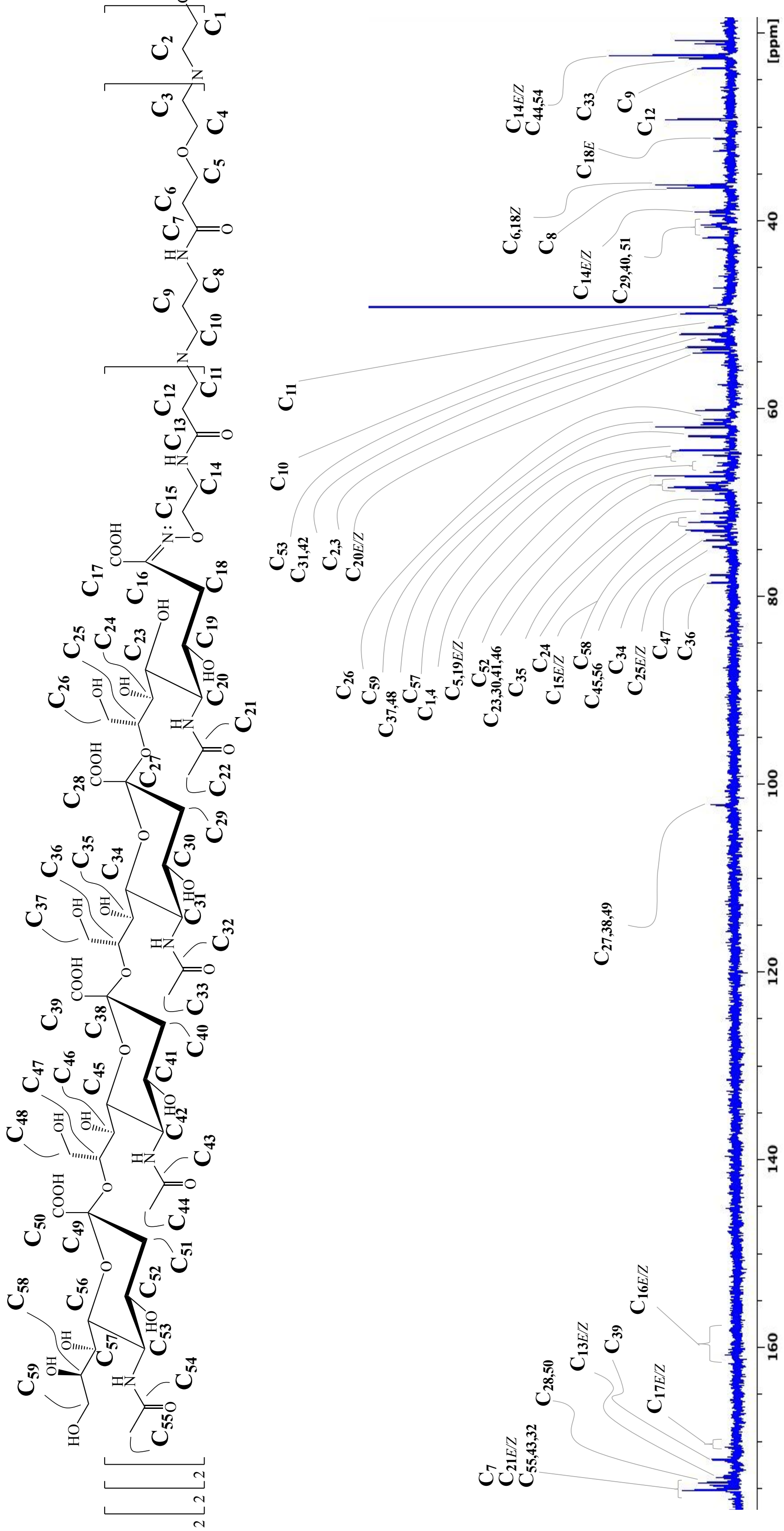

**Figure S26: COSY spectrum of Compound 1 in $D_2O$.**

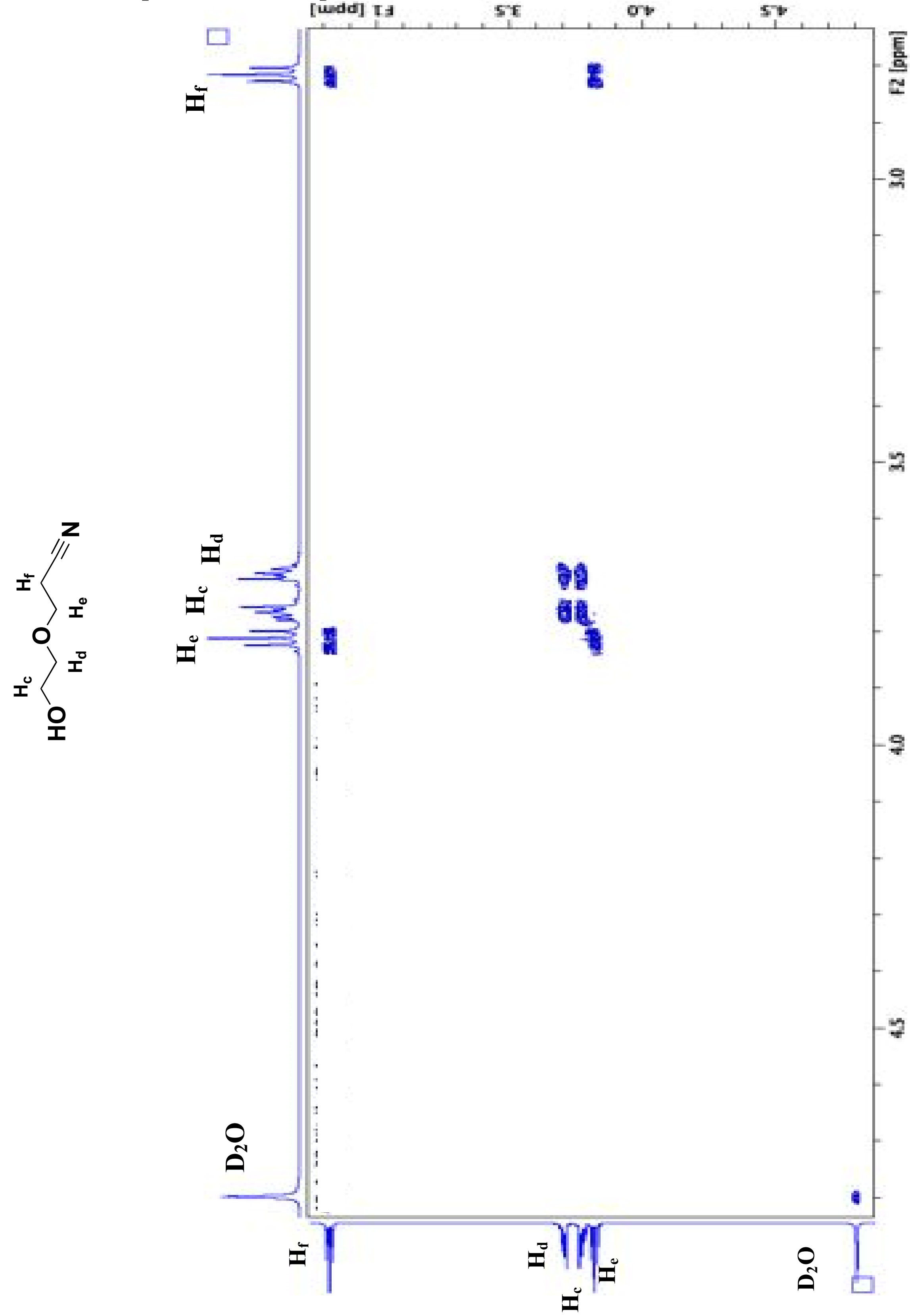

**Figure S27: COSY spectrum of Compound 2 in $CDCl_3$.**

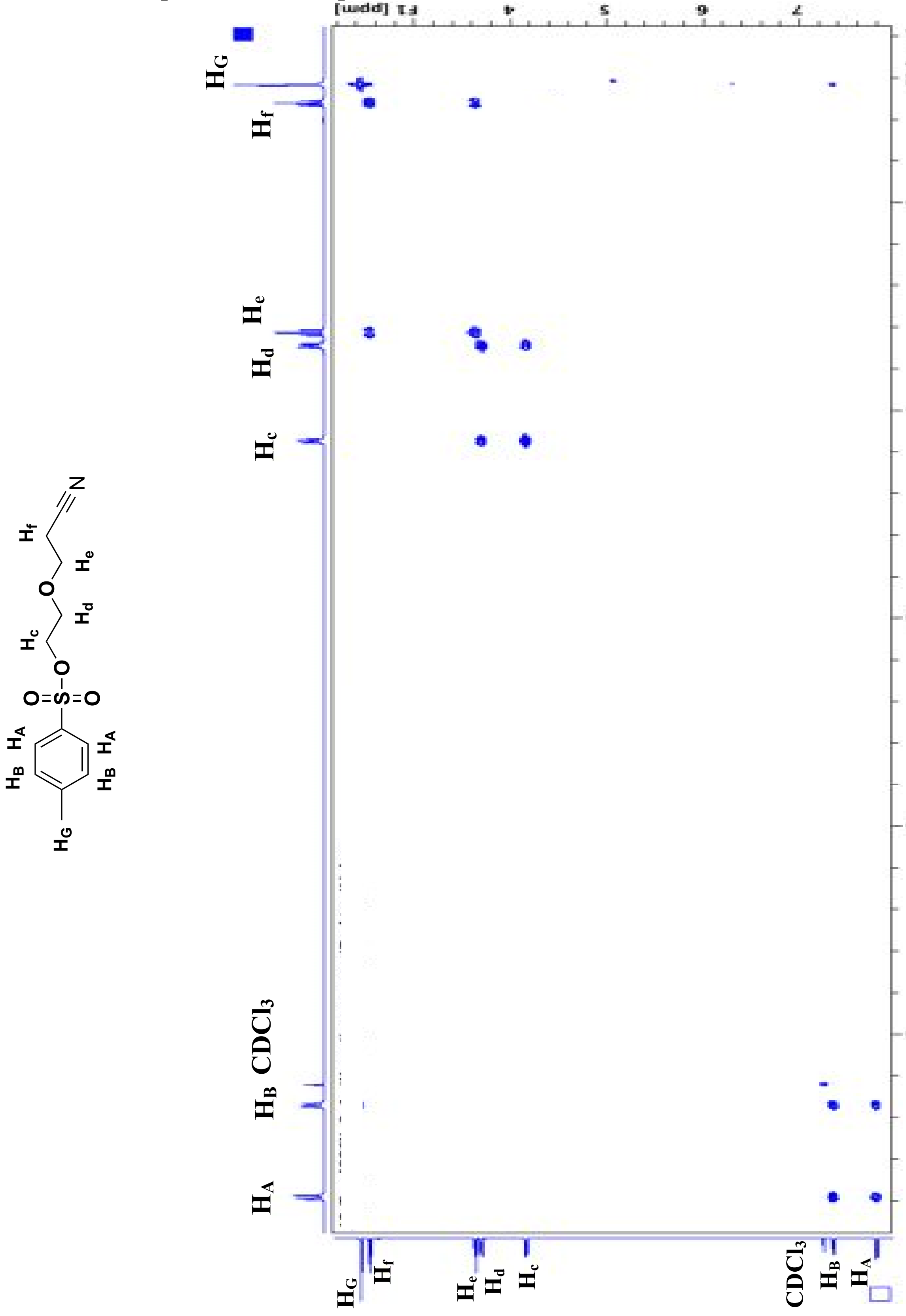

**Figure S28: COSY spectrum of Compound 3 in $D_2O$.**

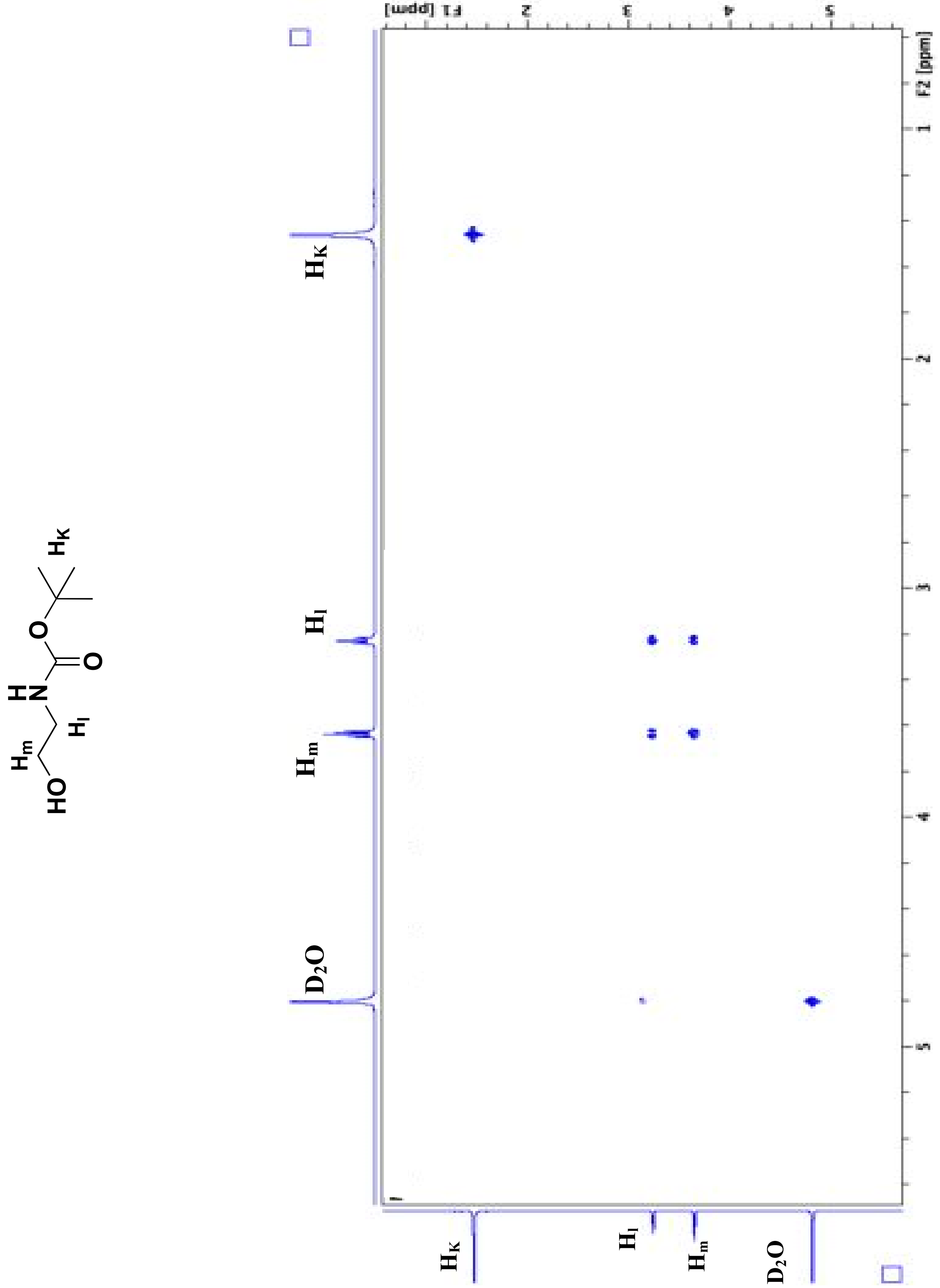

**Figure S29: COSY spectrum of Compound 5 in $D_2O$.**

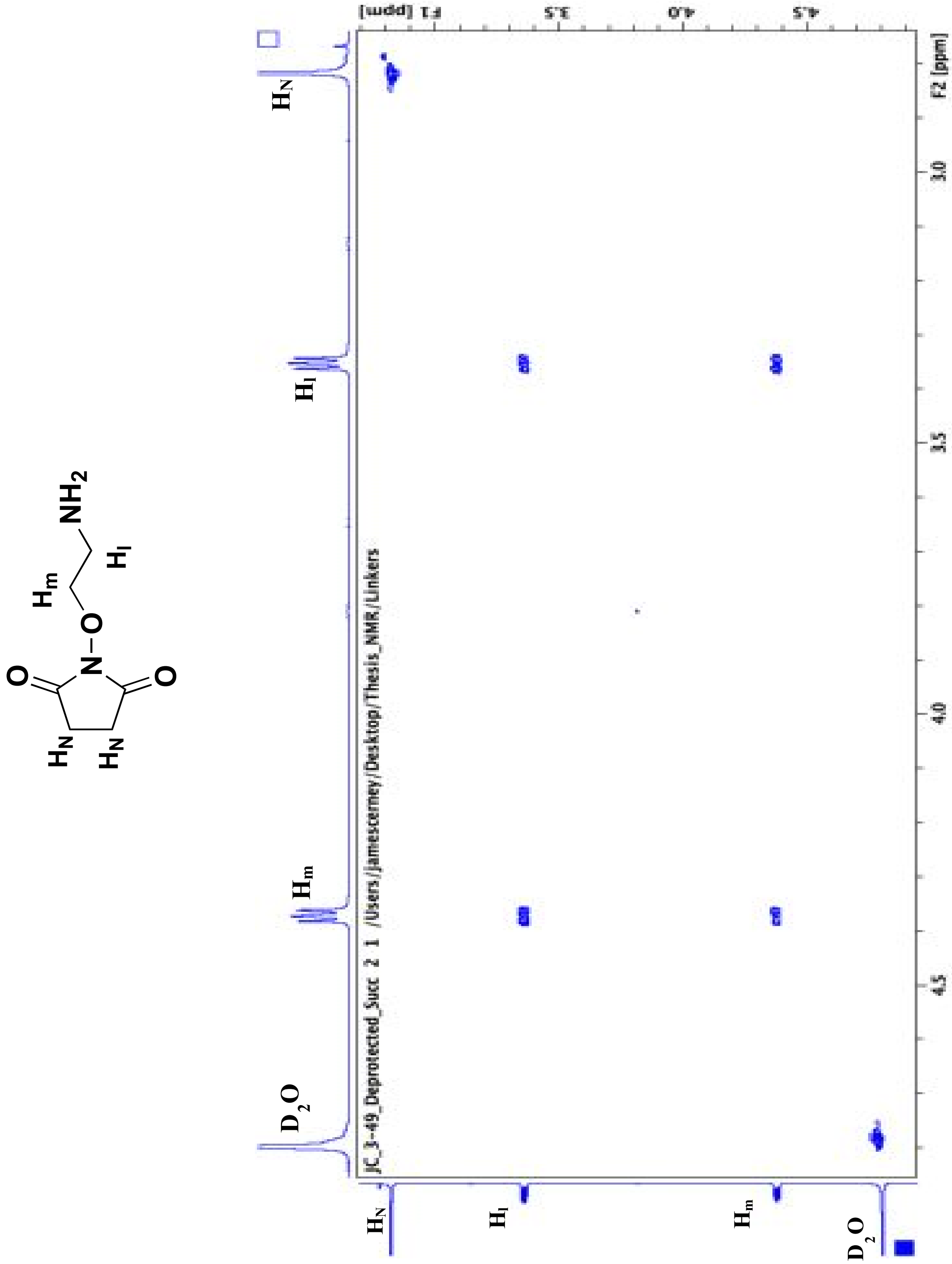

**Figure S30: COSY spectrum of Compound 6 in $D_2O$.**

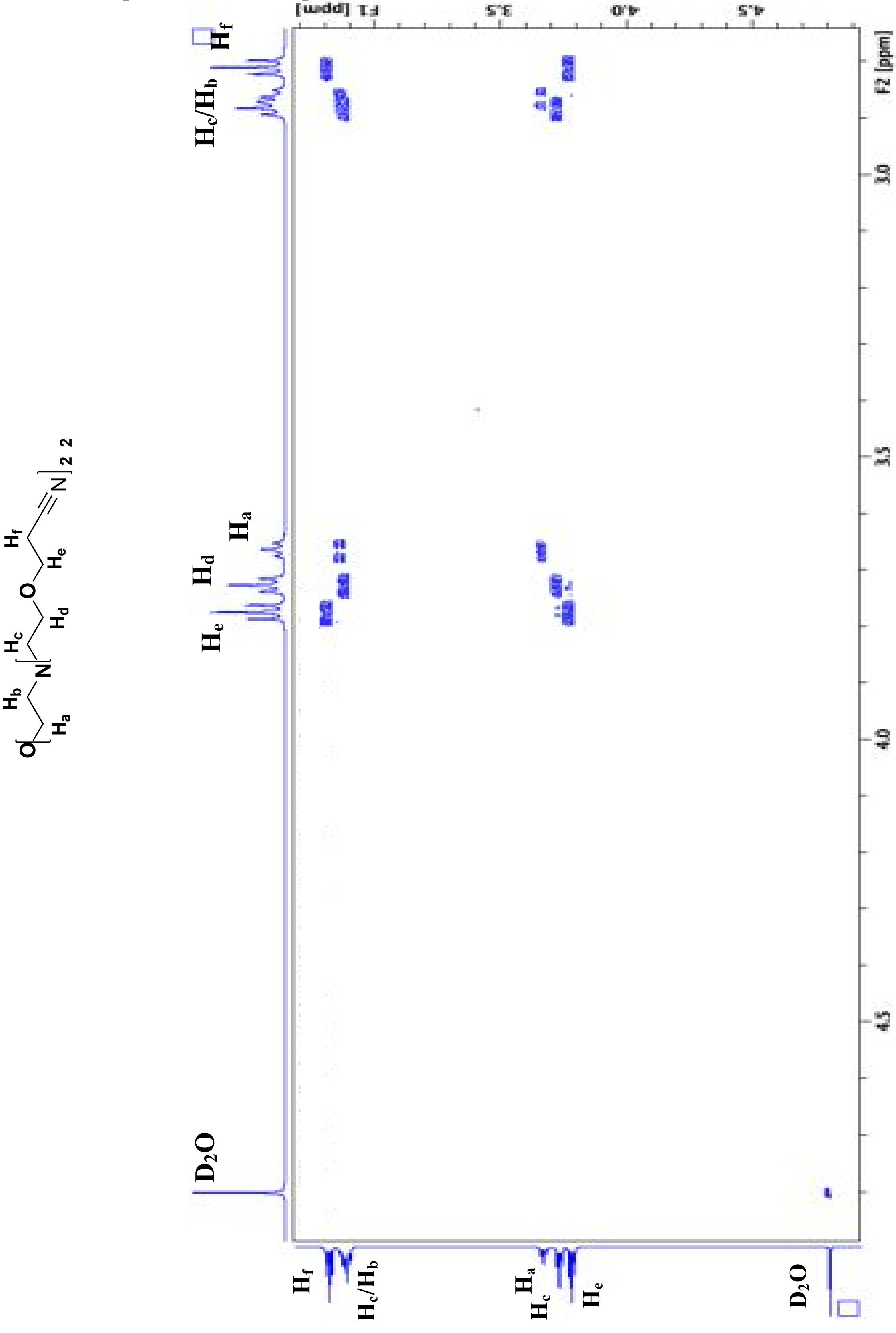

**Figure S31: COSY spectrum of Compound 8 in $D_2O$.**

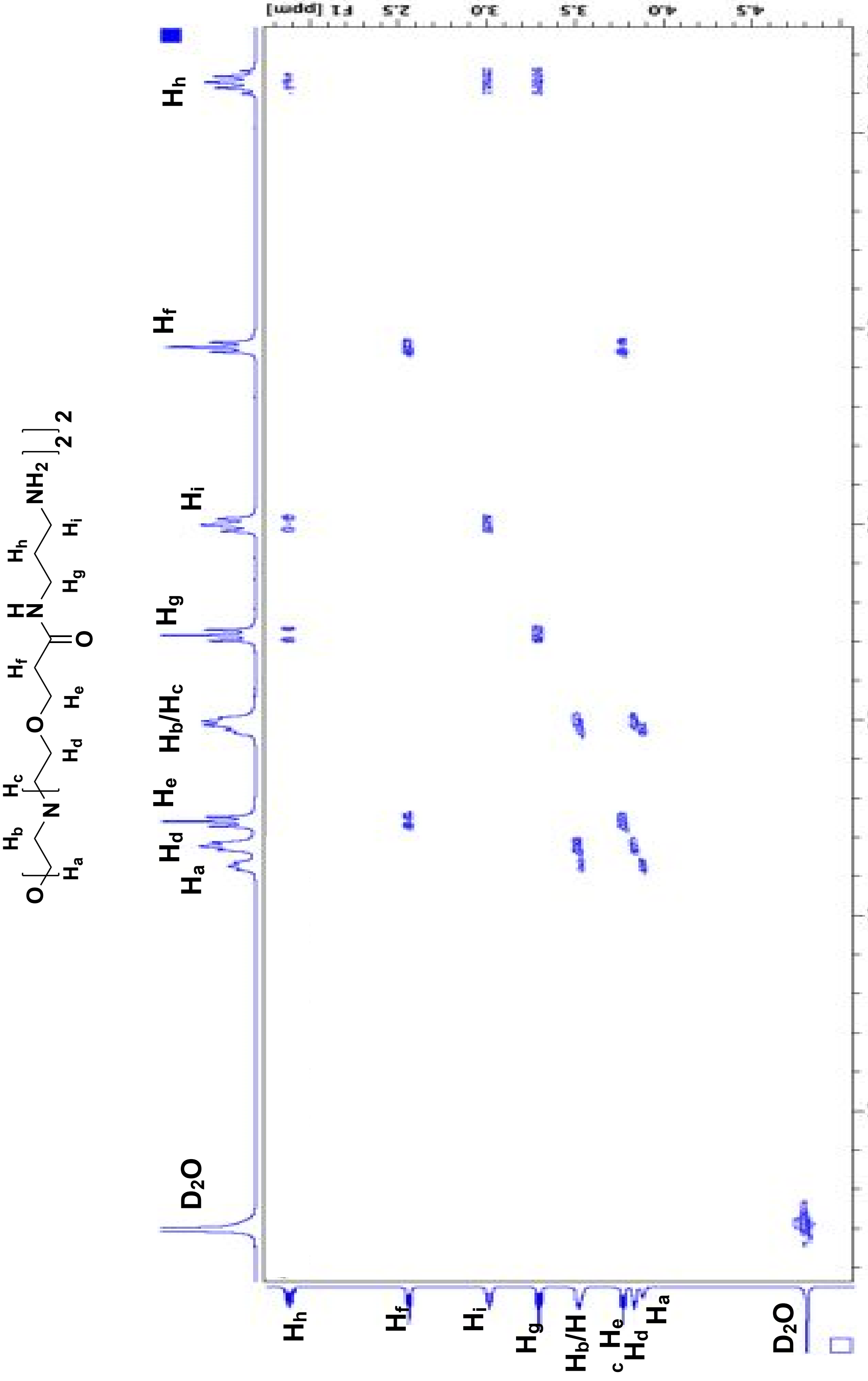

**Figure S32: COSY spectrum of Compound 9 in $D_2O$.**

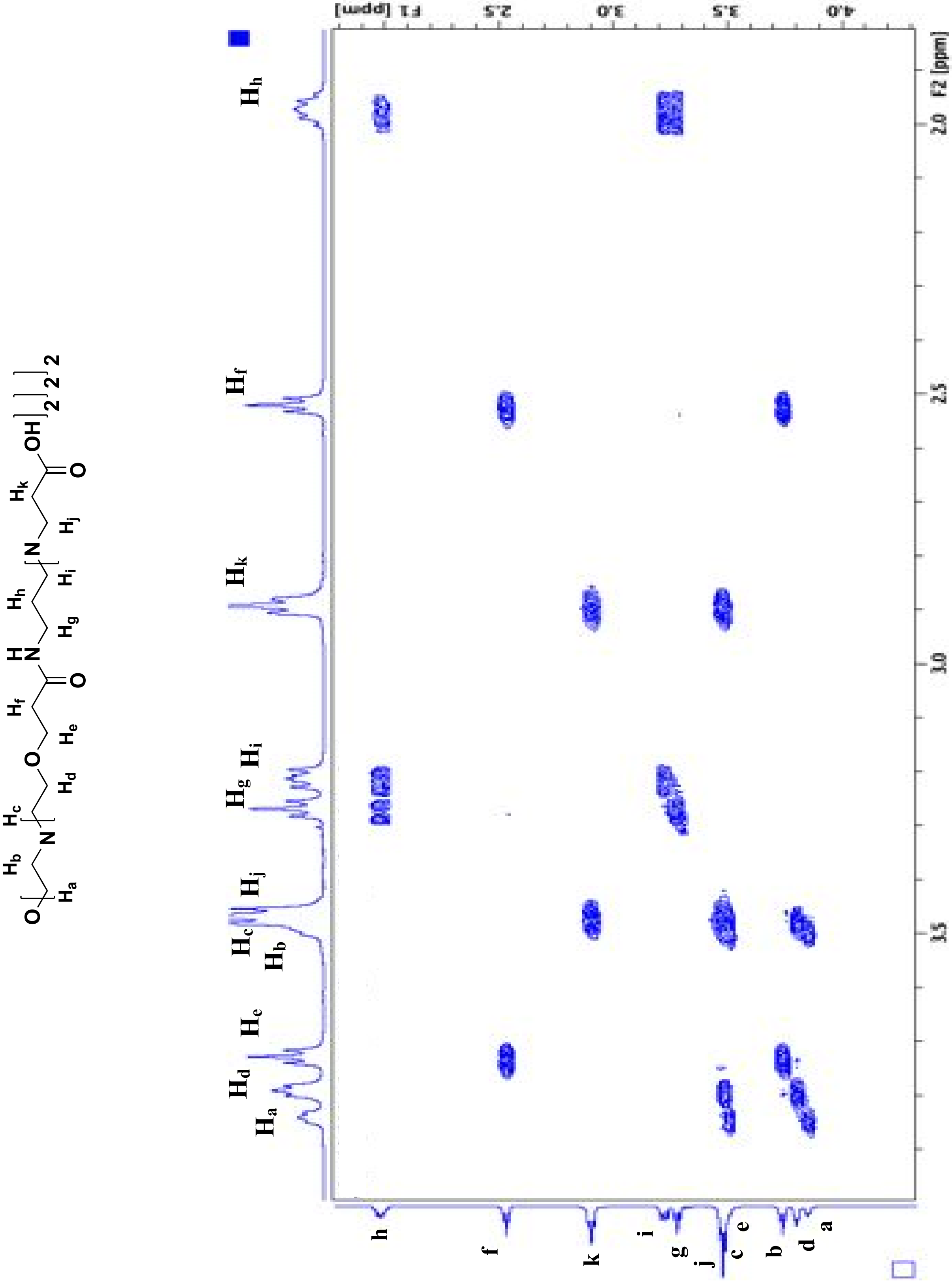

**Figure S33: COSY spectrum of Compound 10 in $D_2O$.**

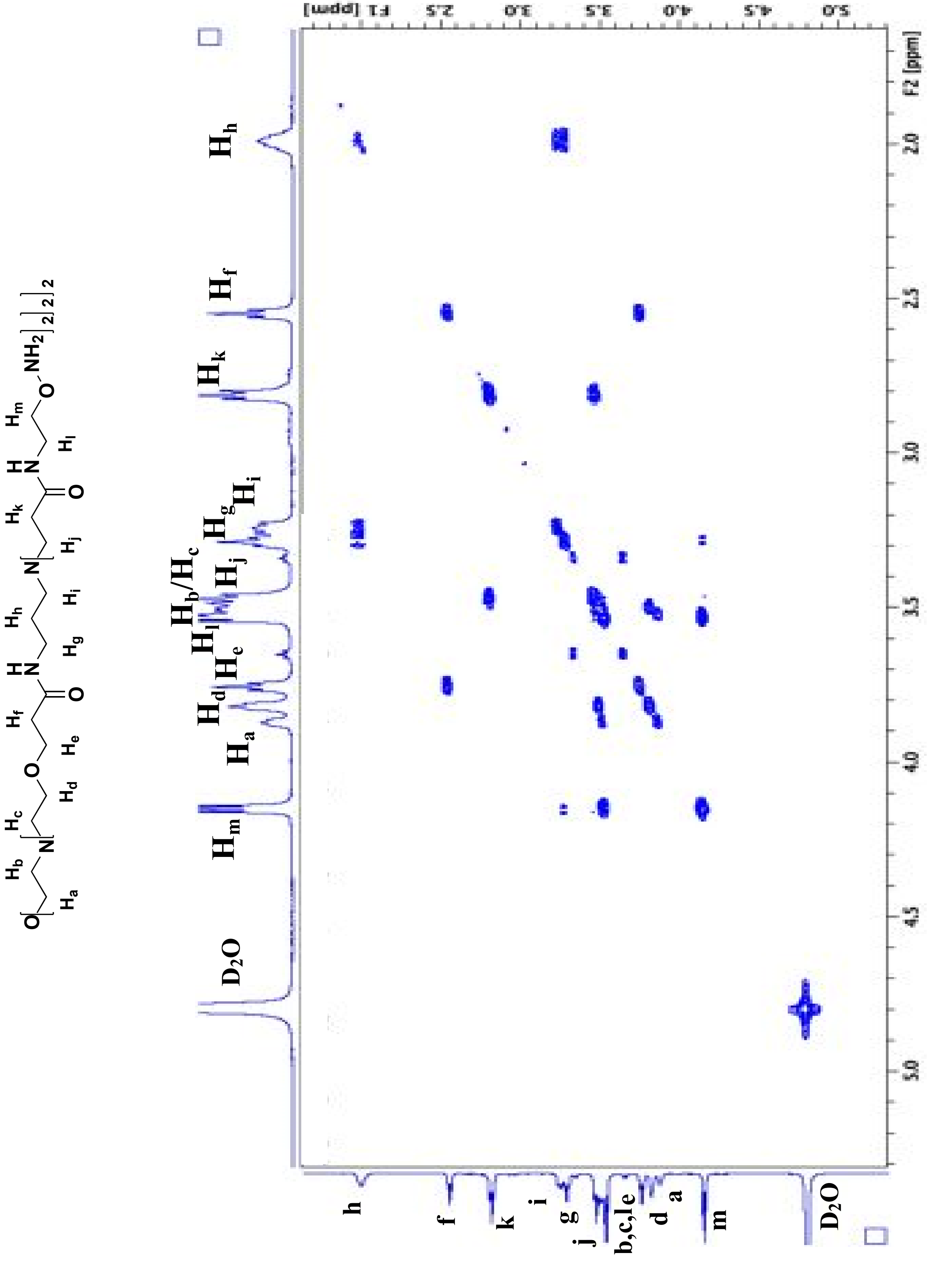

**Figure S34: COSY spectrum of Compound 11 in $D_2O$.**

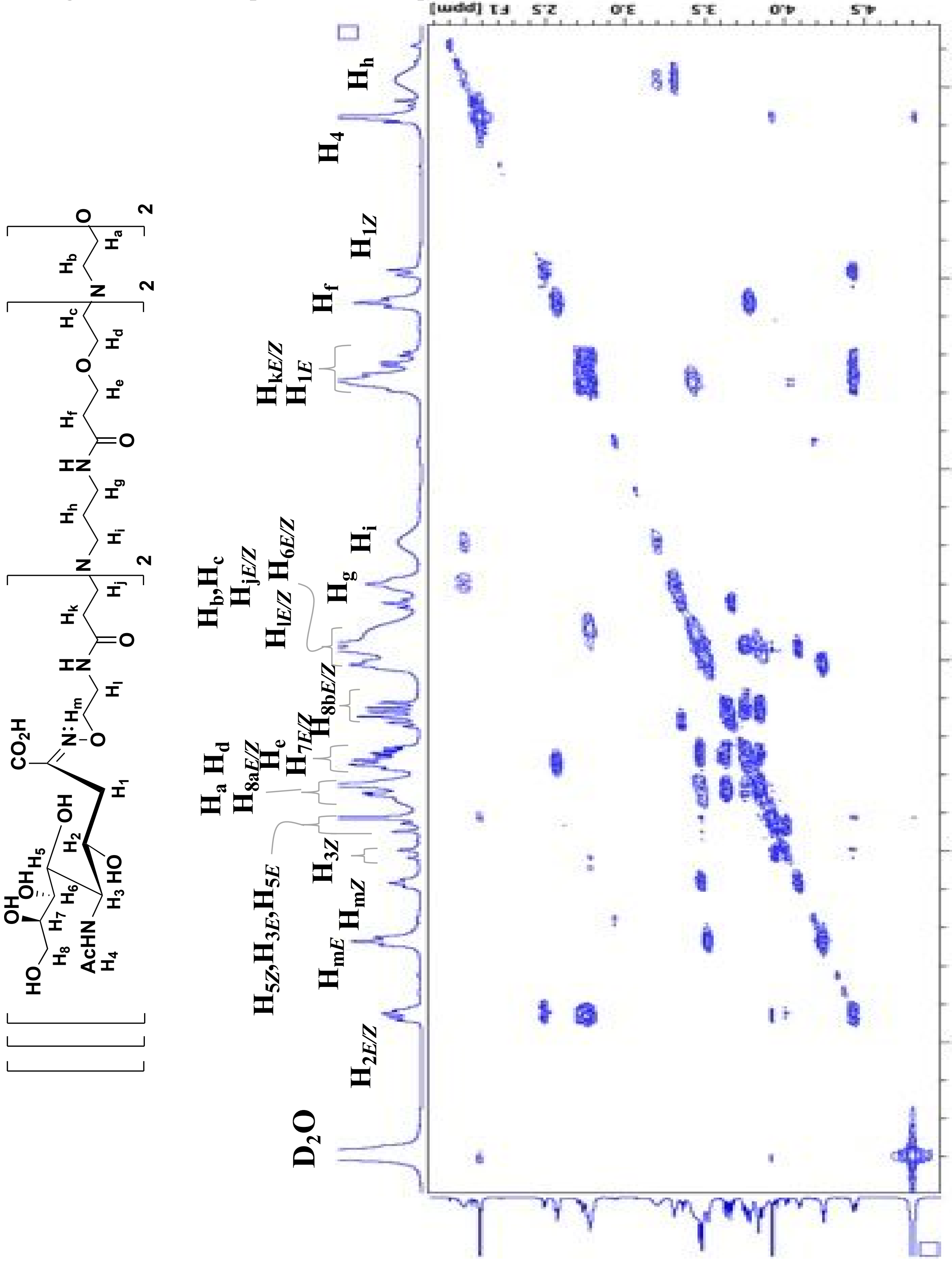

**Figure S35: COSY spectrum of Compound 12 in $D_2O$.**

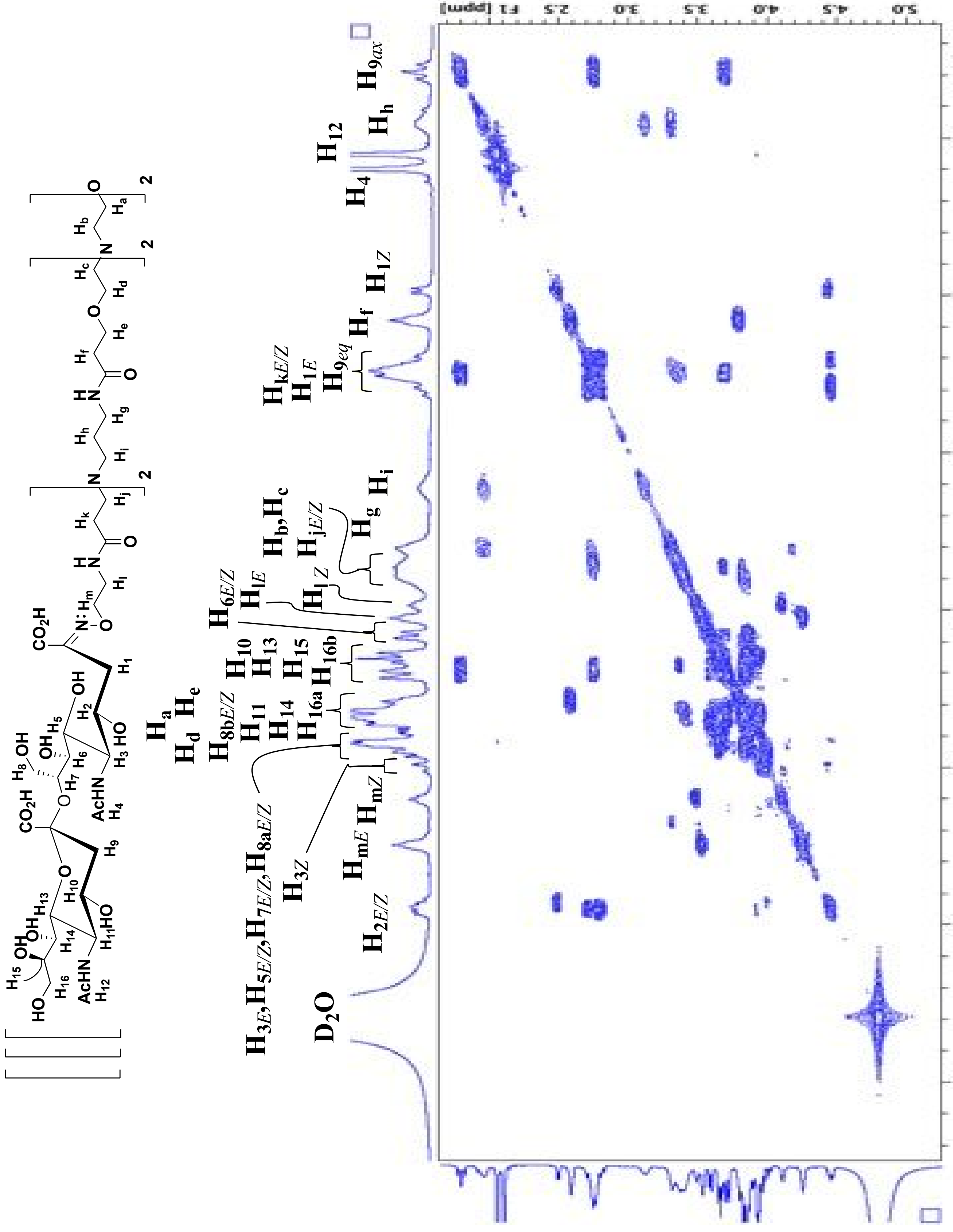

**Figure S36: COSY spectrum of Compound 13 in $D_2O$.**

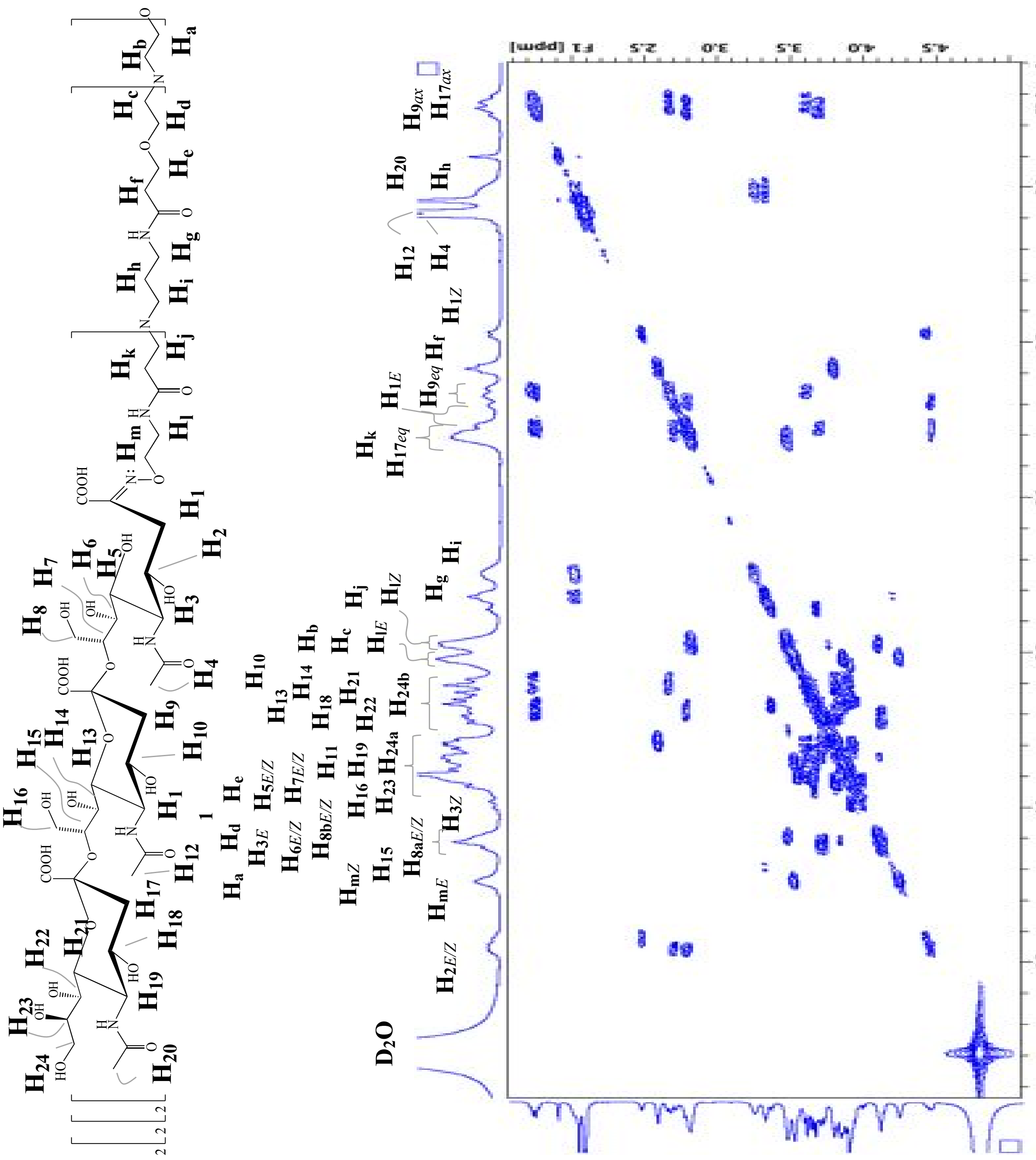

**Figure S37: COSY spectrum of Compound 14 in $D_2O$.**

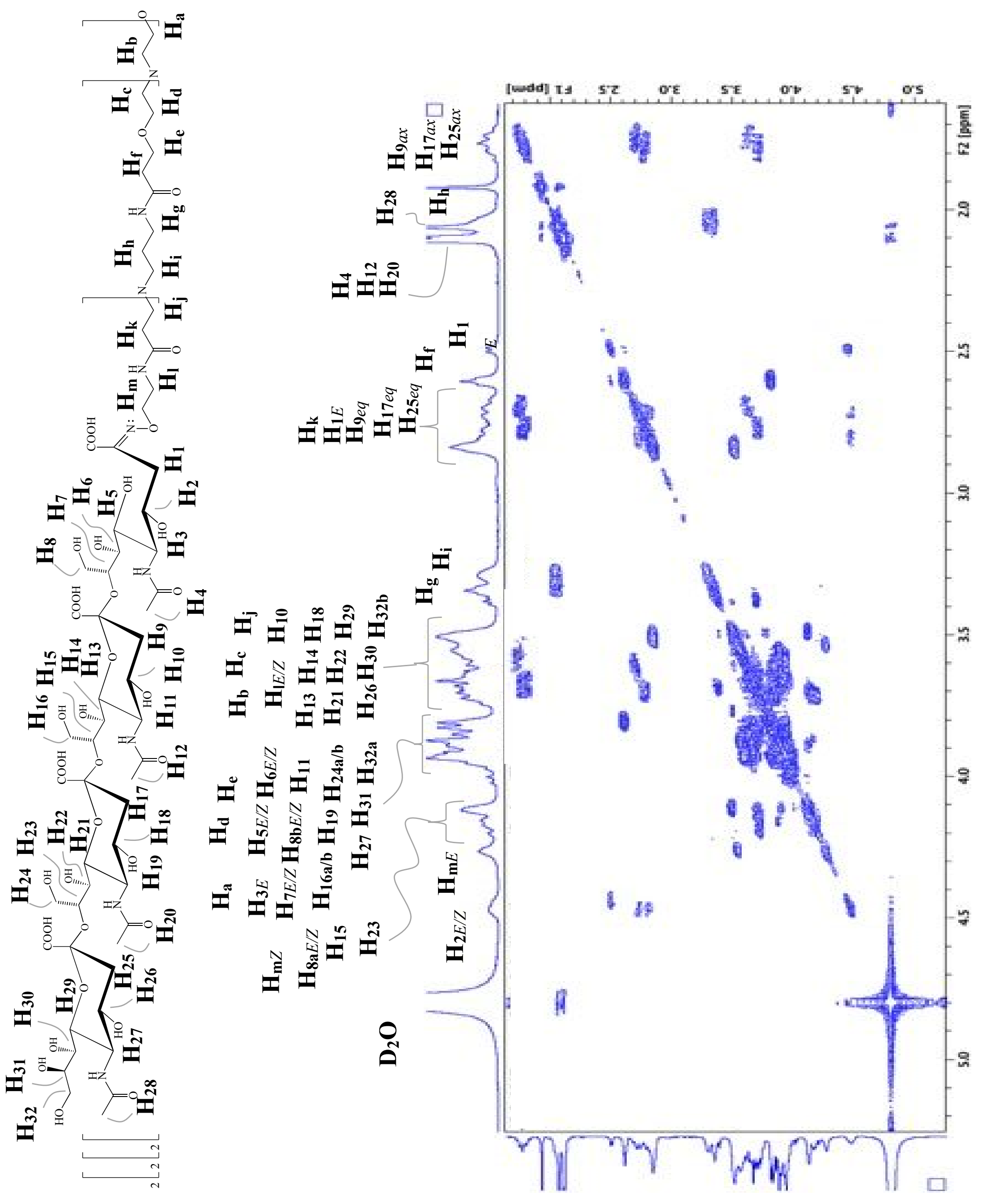

**Figure S38: HSQC spectrum of Compound 1 in $D_2O$ with an internal MeOH standard.**

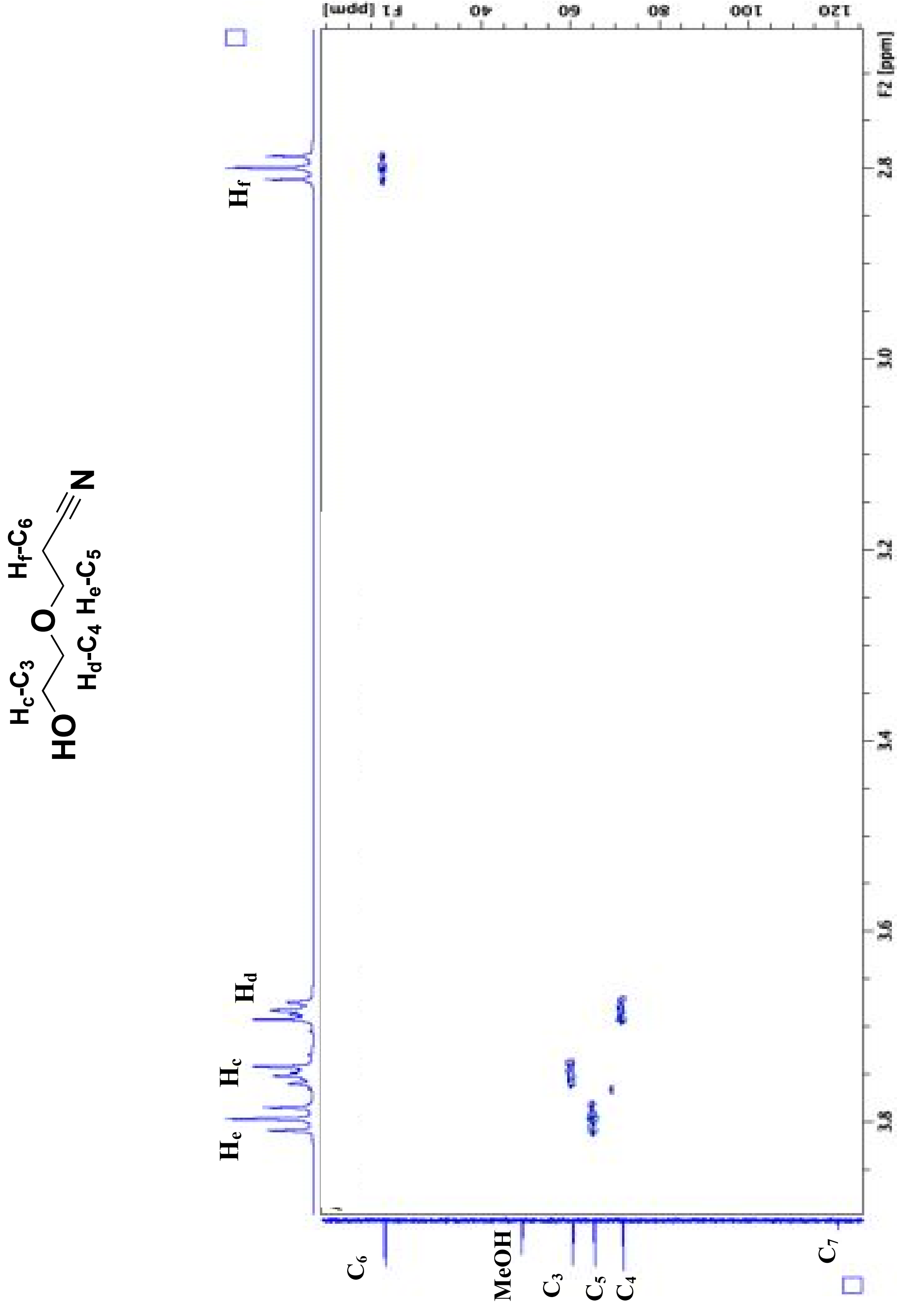

**Figure S39: HSQC spectrum of Compound 2 in $CDCl_3$.**

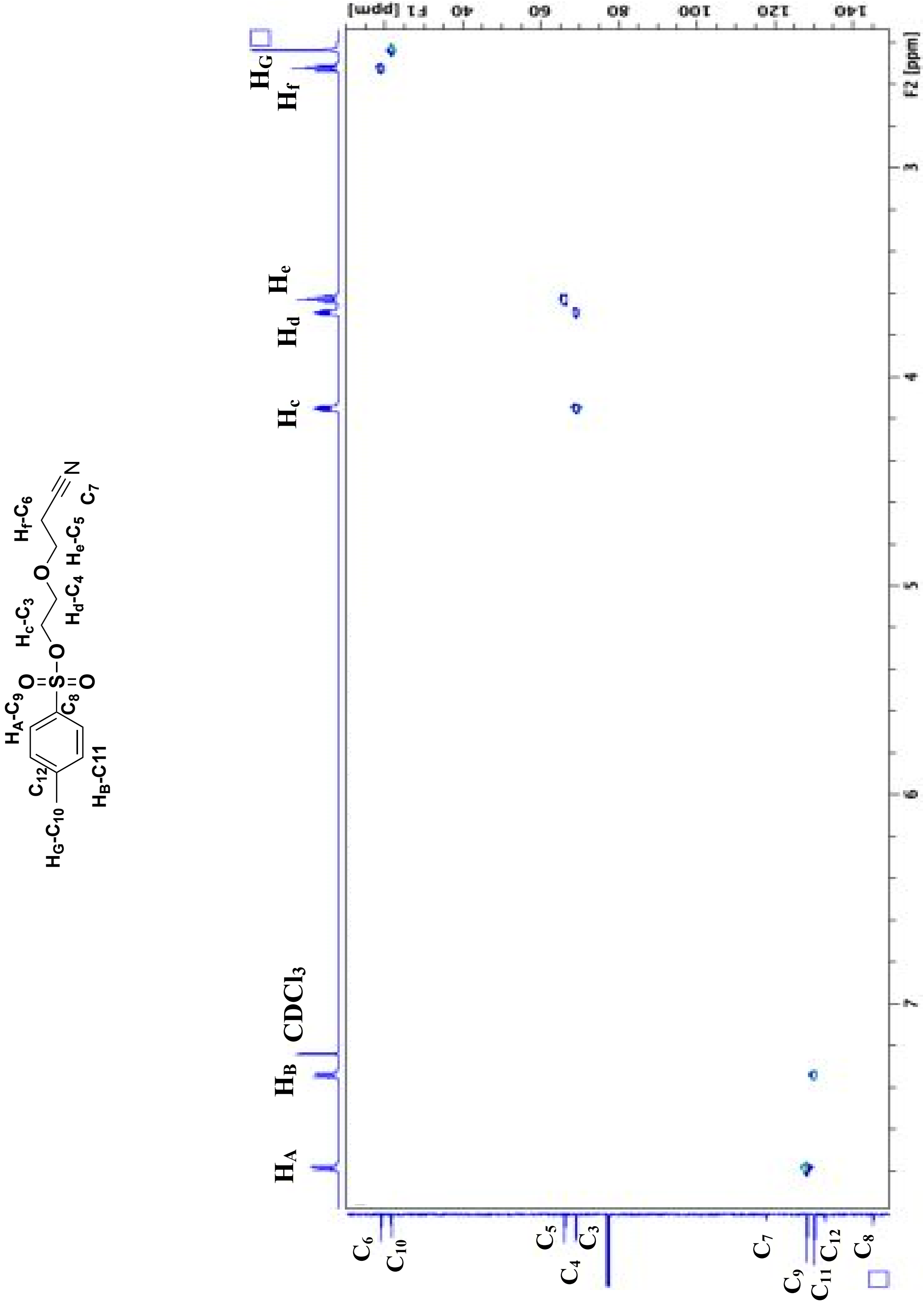

**Figure S40: HSQC spectrum of Compound 6 in $D_2O$ with an internal MeOH standard.**

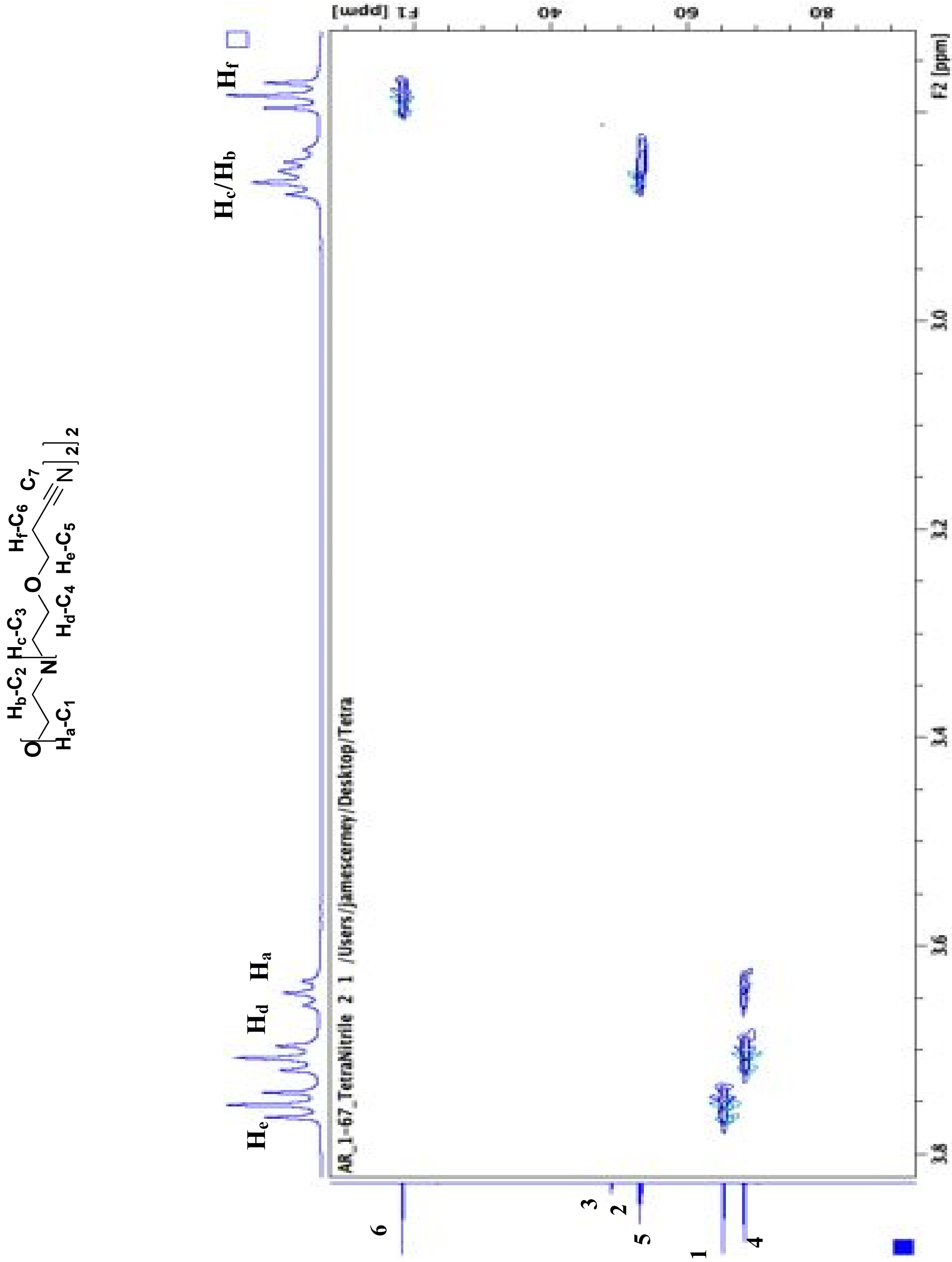

**Figure S41: HSQC spectrum of Compound 8 in $D_2O$ with an internal MeOH standard.**

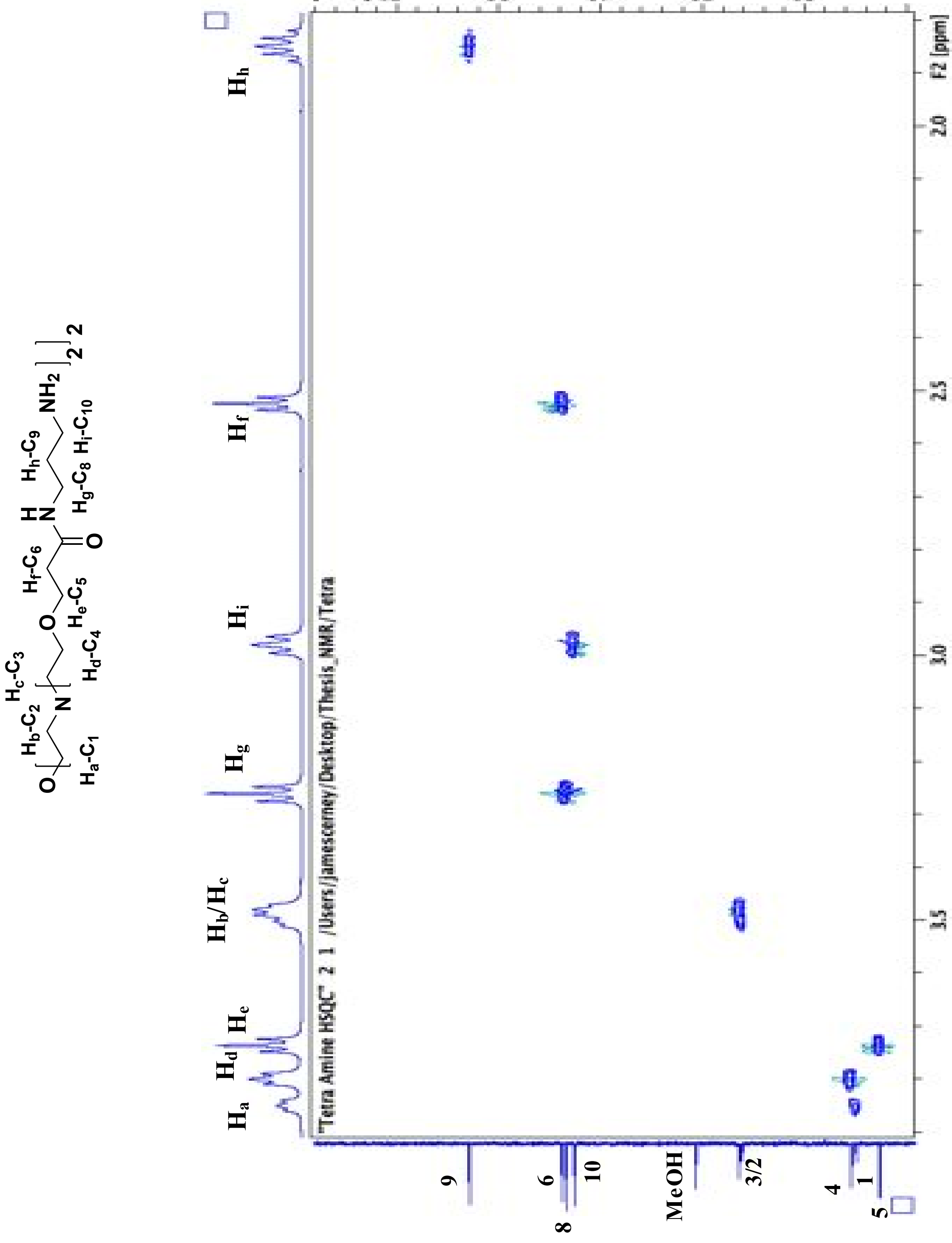

**Figure S42: HSQC spectrum of Compound 9 in $D_2O$ with an internal MeOH standard.**

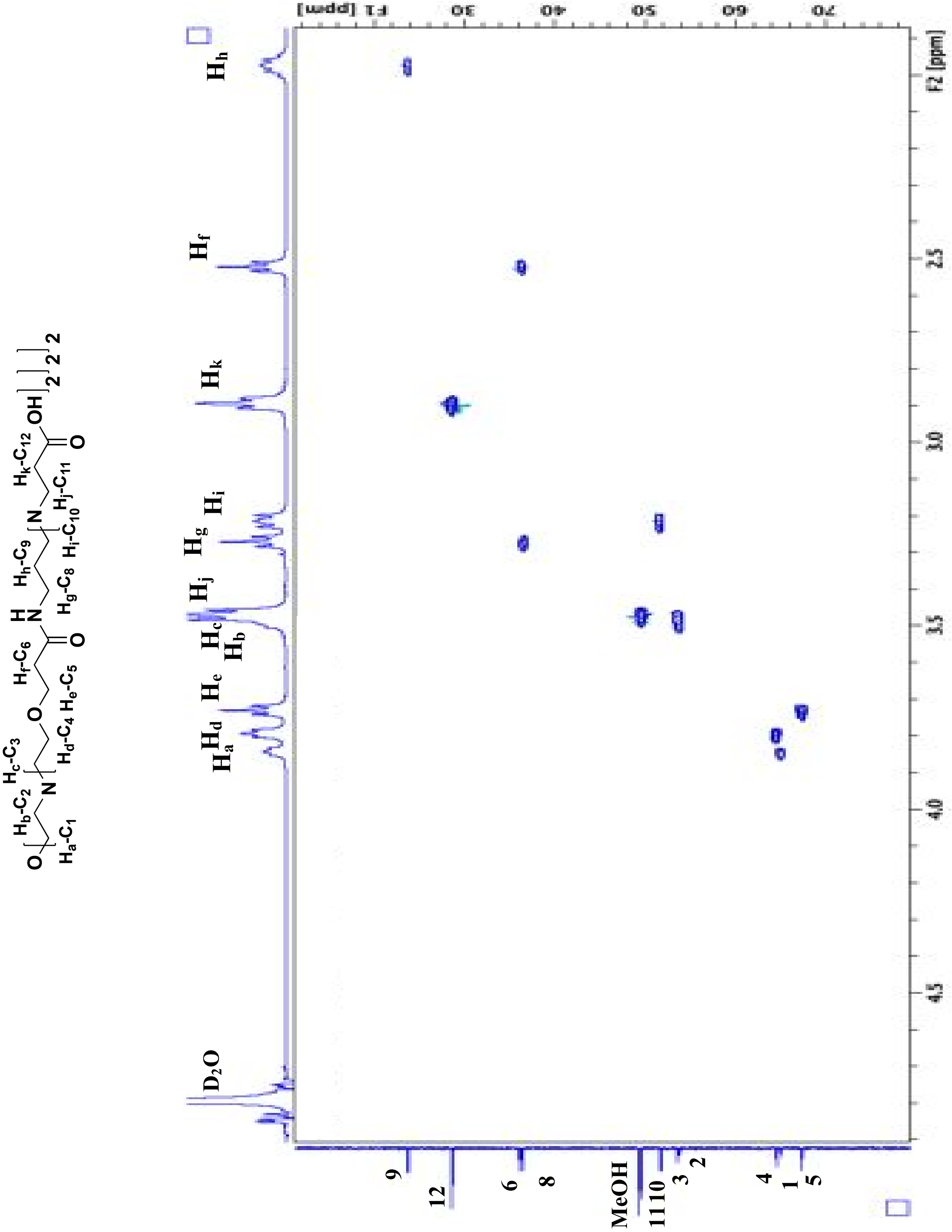

**Figure S43: HSQC spectrum of Compound 10 in $D_2O$ with an internal MeOH standard.**

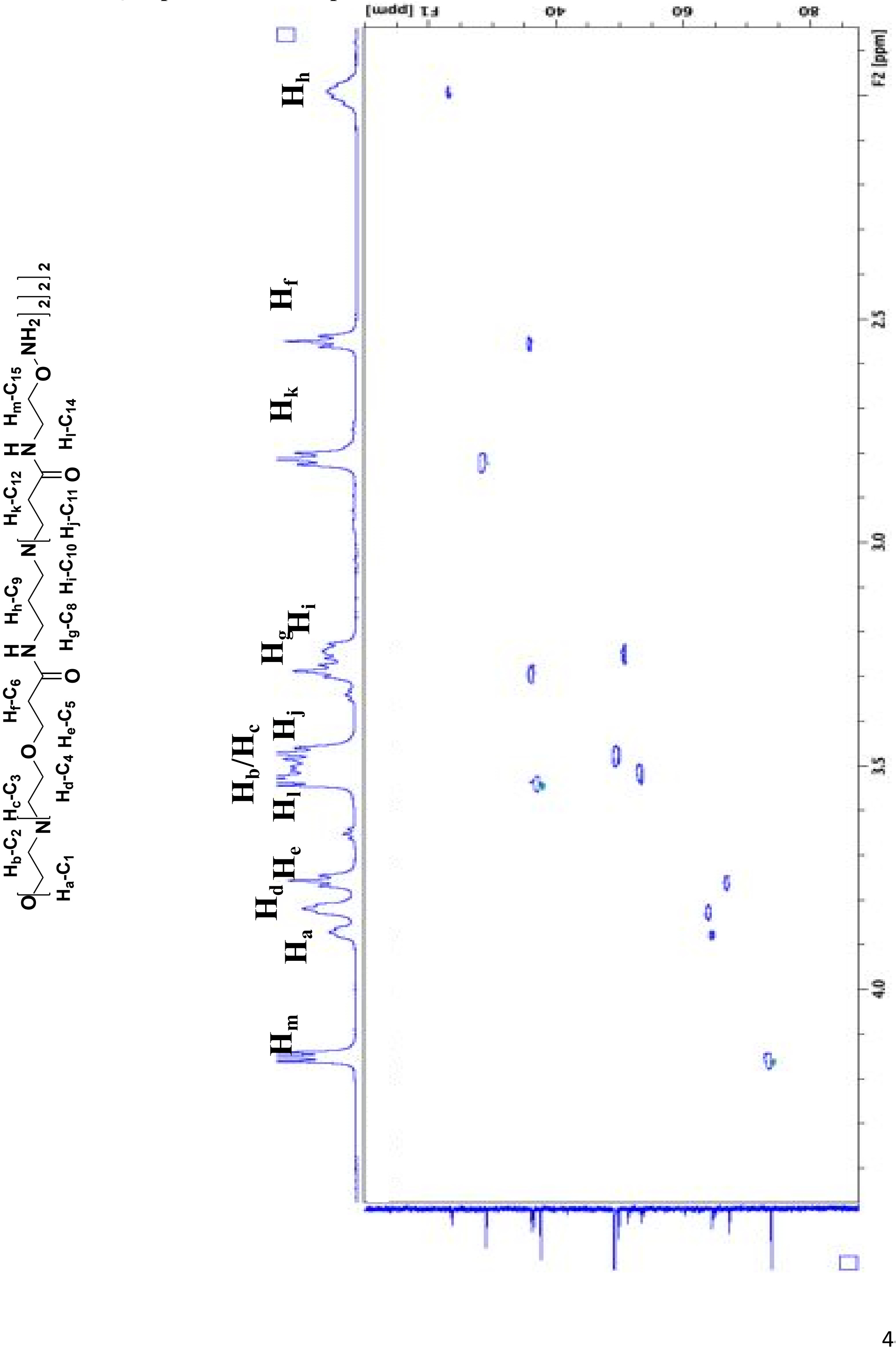

**Figure S44: HSQC spectrum of Compound 11 in $D_2O$ with an internal MeOH standard.**

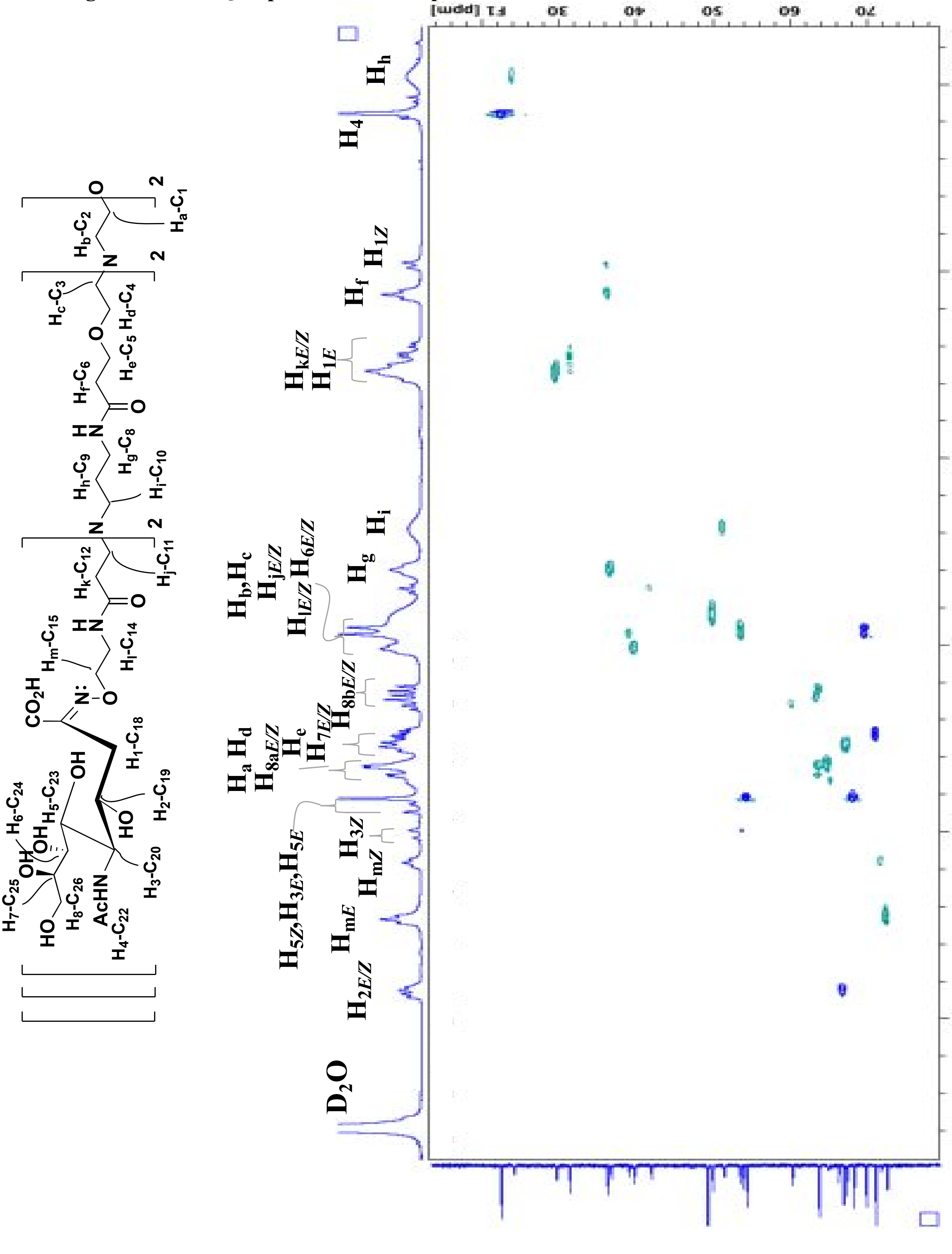

**Figure S45: HSQC spectrum of Compound 12 in $D_2O$ with an internal MeOH standard.**

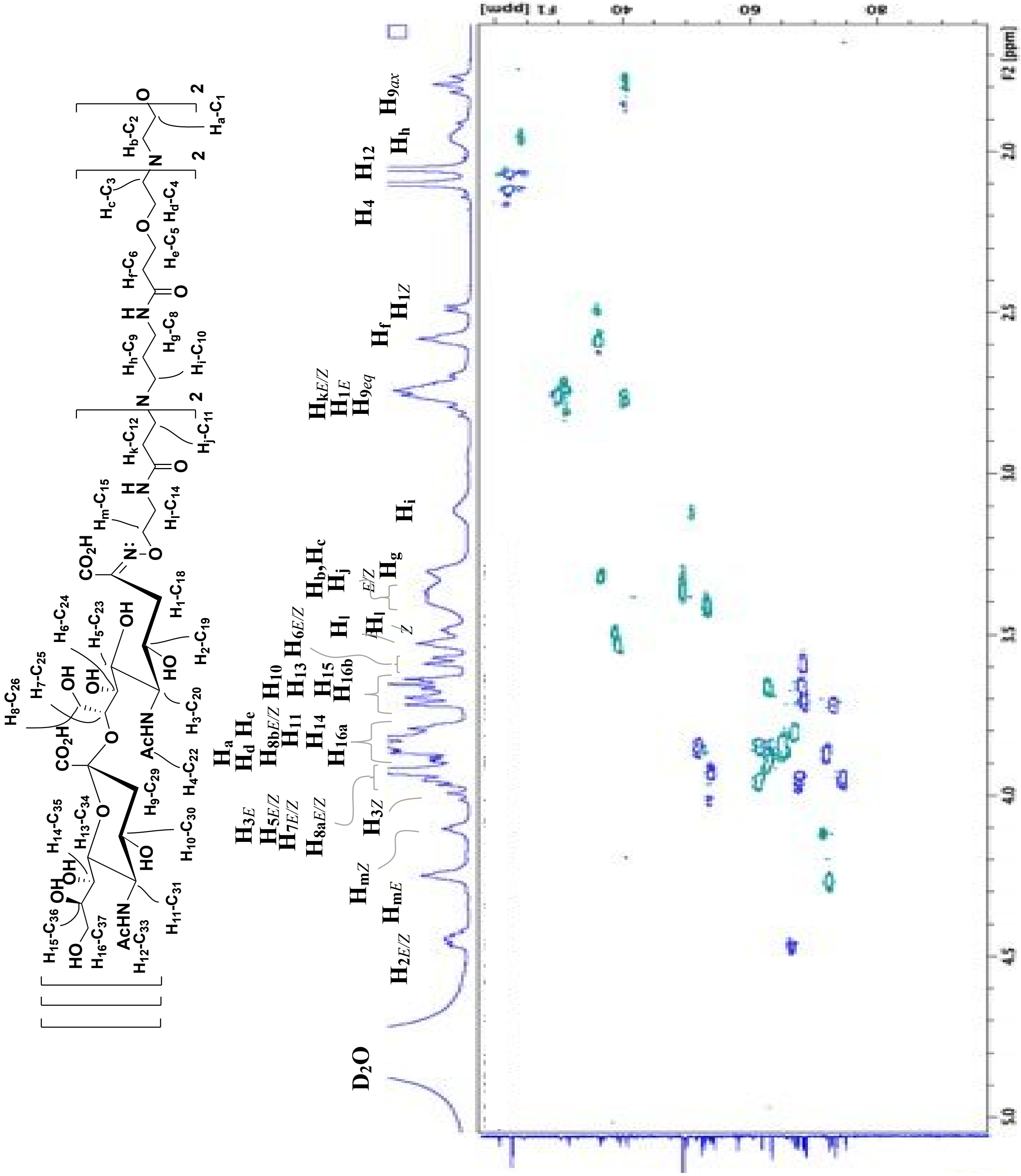

**Figure S46: HSQC spectrum of Compound 13 in $D_2O$ with an internal MeOH standard.**

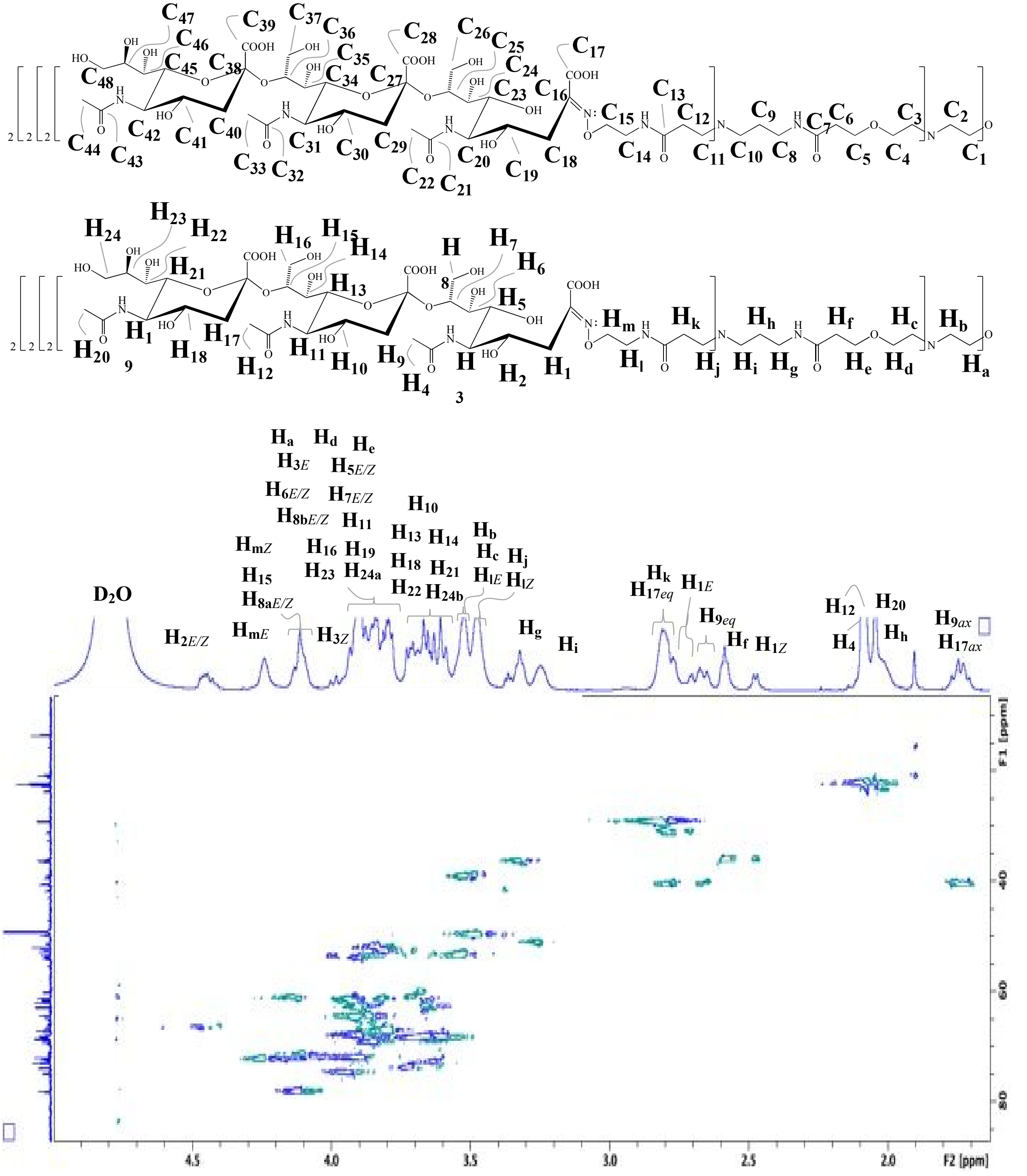


**Figure S47: HSQC spectrum of Compound 14 in $D_2O$ with an internal MeOH standard.**

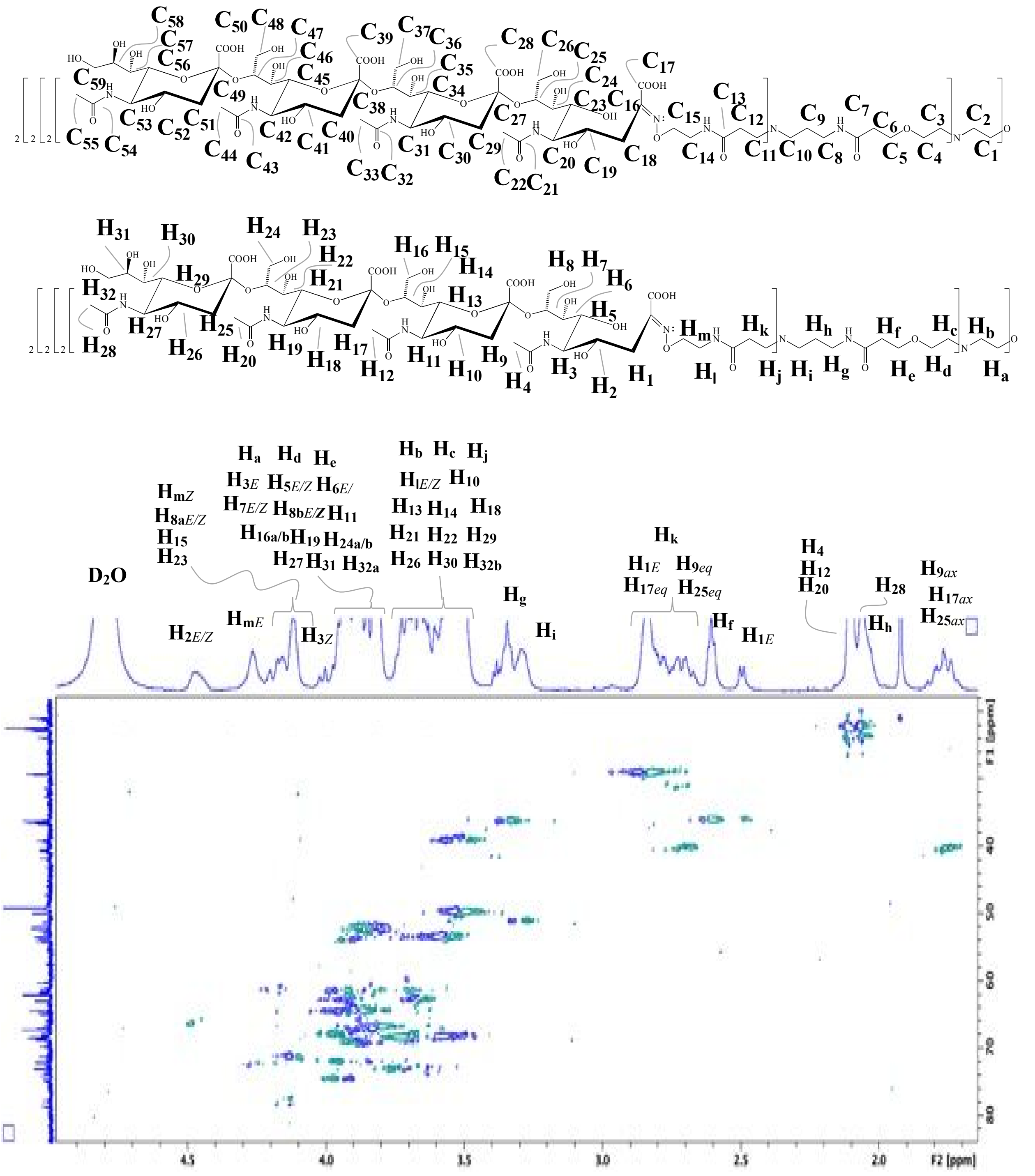


**Table 1:** The full carbon assignments made to **Compound 11** through the use of all the spectra obtained, with D representing the attached sialic acid (with numbering of the sugar ring included).

| Core | | | Sugar | D | |
|---|---|---|---|---|---|
| Position | *E* | *Z* | Position | *E* | *Z* |
| $C_1$ | 65.21 | | 1 | 170.36 | 170.65 |
| $C_2$ | 53.34 | | 2 | 156.5 | 158.15 |
| $C_3$ | 53.34 | | 3 | 31.23 | 36.05 |
| $C_4$ | 64.72 | | 4 | 66.78 | 66.13 |
| $C_5$ | 67.16 | | 5 | 54.21 | 53.70 |
| $C_6$ | 36.15 | | 6 | 68.11 | |
| $C_7$ | 174.39 | | 7 | 69.72 | 69.67 |
| $C_8$ | 36.57 | | 8 | 70.96 | |
| $C_9$ | 23.98 | | 9 | 63.57 | |
| $C_{10}$ | 51.27 | | 10 | 174.78 | 174.75 |
| $C_{11}$ | 49.8 | | 11 | 22.28 | 22.23 |
| $C_{12}$ | 29.45 | | | | |
| $C_{13}$ | 172.2 | | | | |
| $C_{14}$ | 39.54 | 38.89 | | | |
| $C_{15}$ | 72.46 | 71.61 | | | |

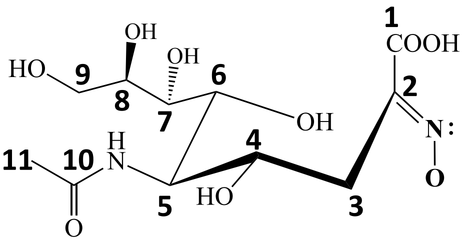


**Table 2:** The full carbon assignments made to **Compound 12** through the use of all the spectra obtained, with D representing the reducing end sialic acid and D' the non-reducing sugar.

| Core | | | Sugar | D | | D' | |
|---|---|---|---|---|---|---|---|
| Position | *E* | *Z* | Position | *E* | *Z* | *E* | *Z* |
| $C_1$ | 65.63 | | 1 | 170.3 | 170.66 | 173.86 | |
| $C_2$ | 53.33 | | 2 | 156.68 | 158.19 | 102.21 | 102.17 |
| $C_3$ | 53.33 | | 3 | 31.26 | 36.08 | 40.46 | |
| $C_4$ | 65.27 | | 4 | 66.76 | 66.13 | 68.55 | |
| $C_5$ | 67.15 | | 5 | 54.06 | 53.33 | 52.05 | |
| $C_6$ | 36.22 | | 6 | 67.89 | | 73.09 | |
| $C_7$ | 174.3 | | 7 | 68.14 | | 68.41 | |
| $C_8$ | 36.82 | | 8 | 74.71 | 74.64 | 72.14 | |
| $C_9$ | 24.16 | | 9 | 63.03 | | 61.52 | |
| $C_{10}$ | 51.13 | | 10 | 175.26 | | 174.71 | |
| $C_{11}$ | 49.69 | | 11 | 22.38 | 22.31 | 22.38 | |
| $C_{12}$ | 29.99 | | | | | | |
| $C_{13}$ | 172.62 | | | | | | |
| $C_{14}$ | 39.39 | 38.91 | | | | | |
| $C_{15}$ | 72.47 | 71.67 | | | | | |

**Table 3:** The full carbon assignments made to **Compound 13** through the use of all the spectra obtained, with D through D'' designating the reducing through non-reducing end of the oligosialic acid residues, respectively.

| Core | | | Sugar | D | | D' | D" |
|---|---|---|---|---|---|---|---|
| Position | *E* | *Z* | Position | *E* | *Z* | | |
| $C_1$ | 64.94 | | 1 | 170.16 | 170.61 | 174.36 | 171.97 |
| $C_2$ | 53.4 | | 2 | 156.67 | 158.14 | 102.45 | 100.93 |
| $C_3$ | 53.4 | | 3 | 31.18 | 36.01 | 40.59 | 40.49 |
| $C_4$ | 64.41 | | 4 | 66.64 | 66.04 | 68.41 | 68.16 |
| $C_5$ | 67.14 | | 5 | 54.01 | 53.68 | 52.76 | 52.04 |
| $C_6$ | 36.14 | | 6 | 68.01 | | 73.87 | 72.92 |
| $C_7$ | 174.93 | | 7 | 69.25 | | 68.32 | 68.71 |
| $C_8$ | 36.47 | | 8 | 74.85 | 74.78 | 78.12 | 72.07 |
| $C_9$ | 23.73 | | 9 | 61.29 | | 61.96 | 61.56 |
| $C_{10}$ | 51.26 | | 10 | 175.20 | 175.16 | 174.65 | 175.20 |
| $C_{11}$ | 49.84 | | 11 | 22.36 | 22.3 | 22.63 | 22.36 |
| $C_{12}$ | 29.17 | | | | | | |
| $C_{13}$ | 173.6 | | | | | | |
| $C_{14}$ | 39.39 | 38.88 | | | | | |
| $C_{15}$ | 72.43 | 71.62 | | | | | |

**Table 4:** The full carbon assignments made to **Compound 14** through the use of all the spectra obtained, with D through D''' designating the reducing through non-reducing end of the oligosialic acid sugar residues, respectively.

| Core | | | Sugar | D | | D' | | D'' | | D''' | |
|---|---|---|---|---|---|---|---|---|---|---|---|
| Position | *E* | *Z* | Position | *E* | *Z* | *E* | *Z* | | | | |
| $C_1$ | 65.71 | | 1 | 170.22 | 170.61 | 174.35 | | 171.87 | | 174.64 | |
| $C_2$ | 53.41 | | 2 | - | - | 102.18 | | 102.18 | | 102.18 | |
| $C_3$ | 53.41 | | 3 | 31.19 | 36.02 | 40.61 | | 40.41 | | 40.25 | |
| $C_4$ | 64.93 | | 4 | 66.04 | 65.71 | 68.44 | | 68.33 | | 67.14 | |
| $C_5$ | 66.72 | | 5 | 54.00 | 53.69 | 52.80 | | 52.62 | | 52.04 | |
| $C_6$ | 36.13 | | 6 | 68.27 | | 74.00 | | 73.04 | | 72.89 | |
| $C_7$ | 174.92 | | 7 | 71.13 | | 69.65 | | 68.69 | | 64.40 | |
| $C_8$ | 36.46 | | 8 | 74.68 | | 78.51 | | 77.68 | | 72.03 | |
| $C_9$ | 23.72 | | 9 | 61.57 | | 62.97 | | 62.88 | | 61.95 | |
| $C_{10}$ | 51.35 | | 10 | 175.20 | | 175.12 | | 175.22 | | 175.20 | |
| $C_{11}$ | 49.86 | | 11 | 22.35 | 22.29 | 22.71 | | 22.61 | | 22.35 | |
| $C_{12}$ | 29.17 | | | | | | | | | | |
| $C_{13}$ | 173.82 | | | | | | | | | | |
| $C_{14}$ | 39.38 | 38.88 | | | | | | | | | |
| $C_{15}$ | 72.44 | 71.55 | | | | | | | | | |

**Figure S48: IR spectrum of Compound 6.**

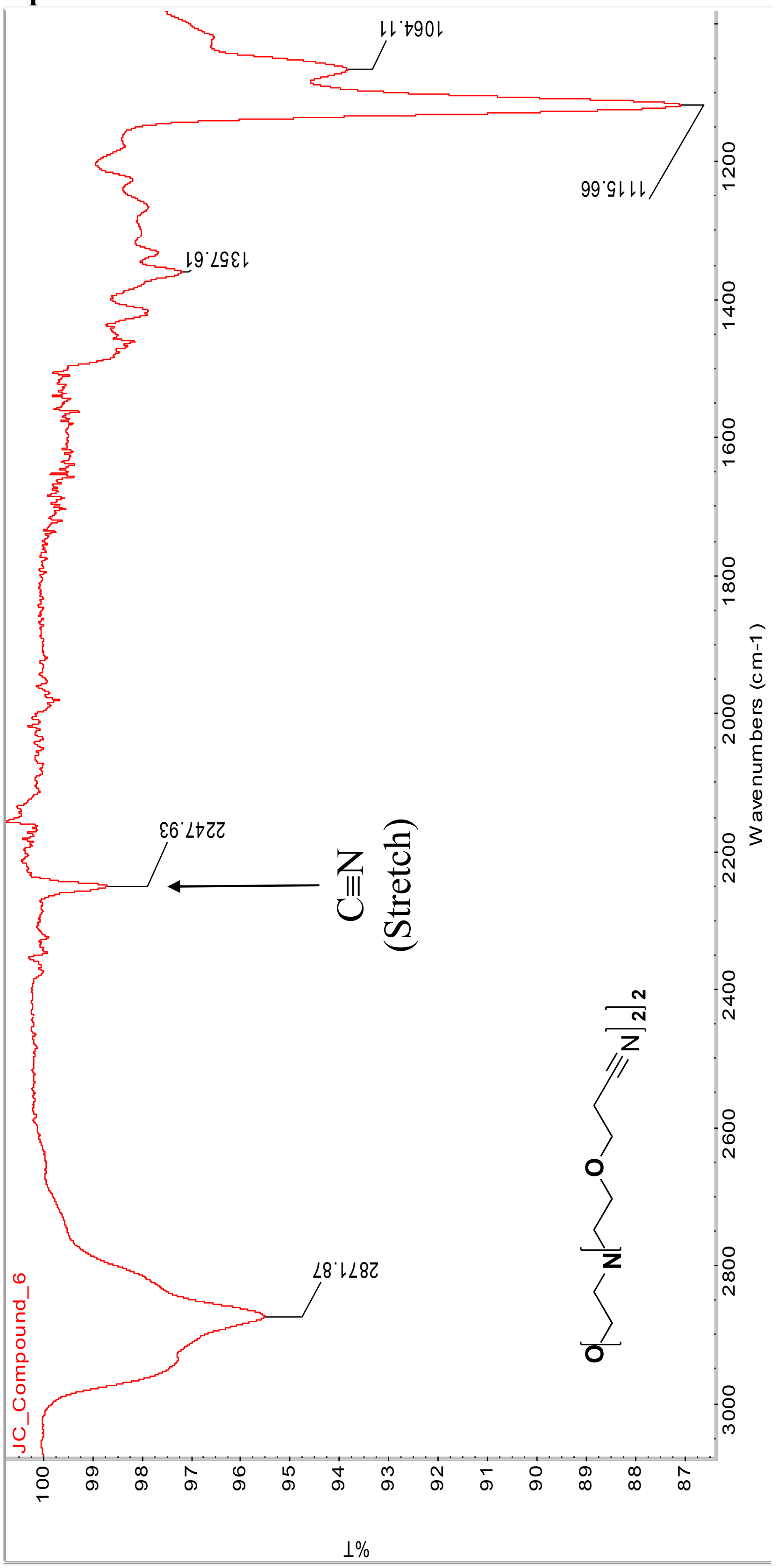

**Figure S49: IR spectrum of Compound 7.**

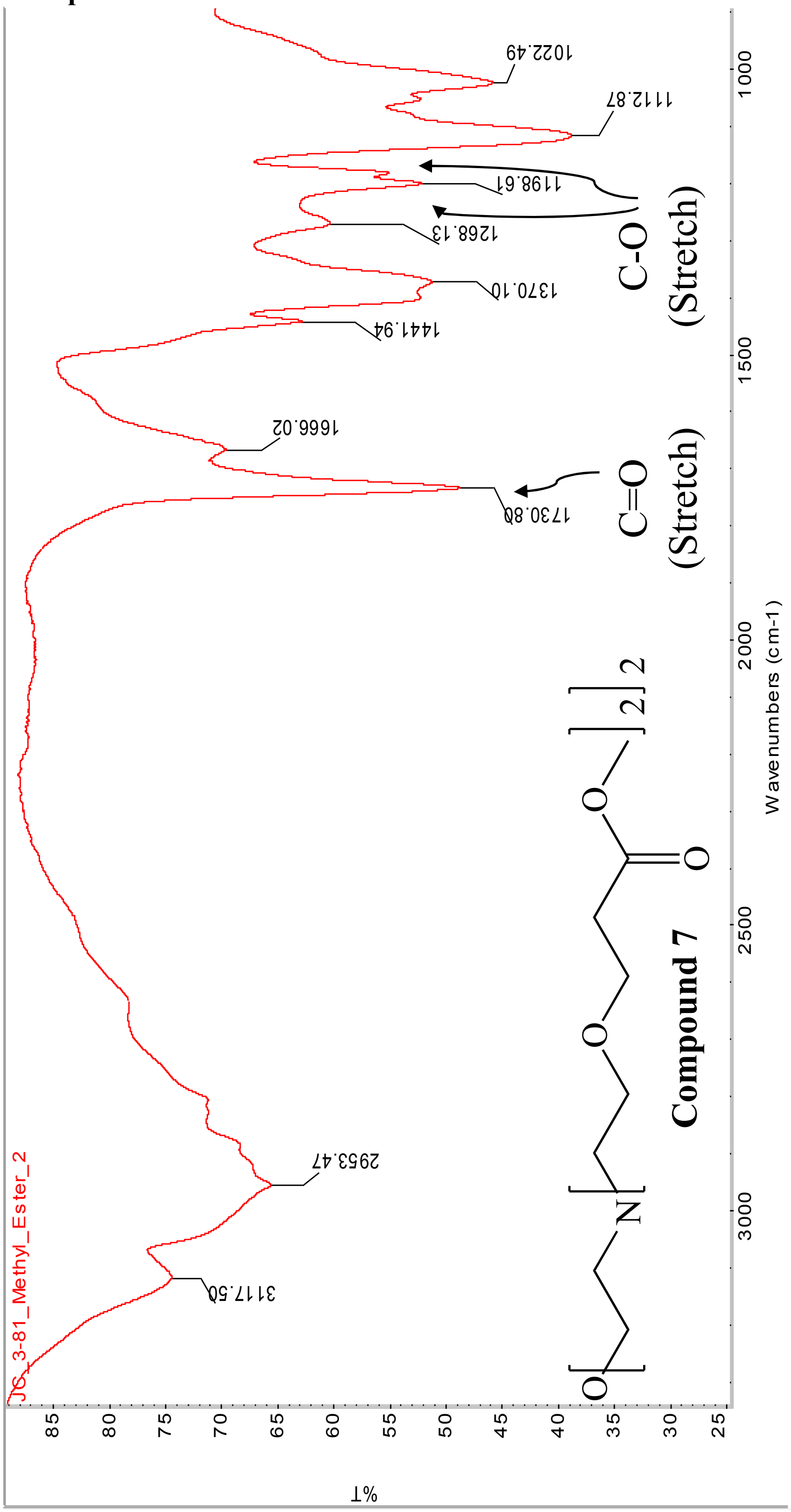

**Figure S50: HR-ESI+ spectrum of Compound 6.**

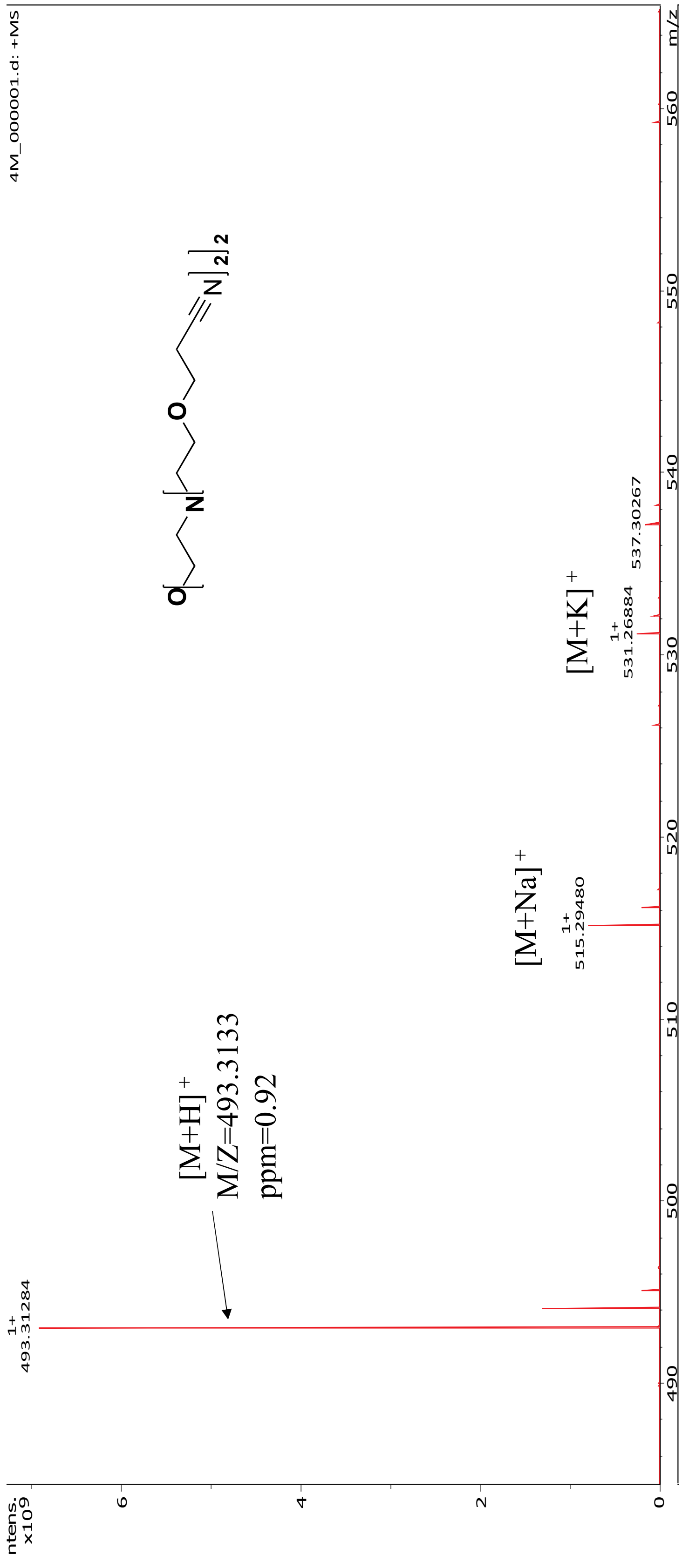

**Figure S51: HR-ESI$^{+}$ spectrum of Compound 8.**

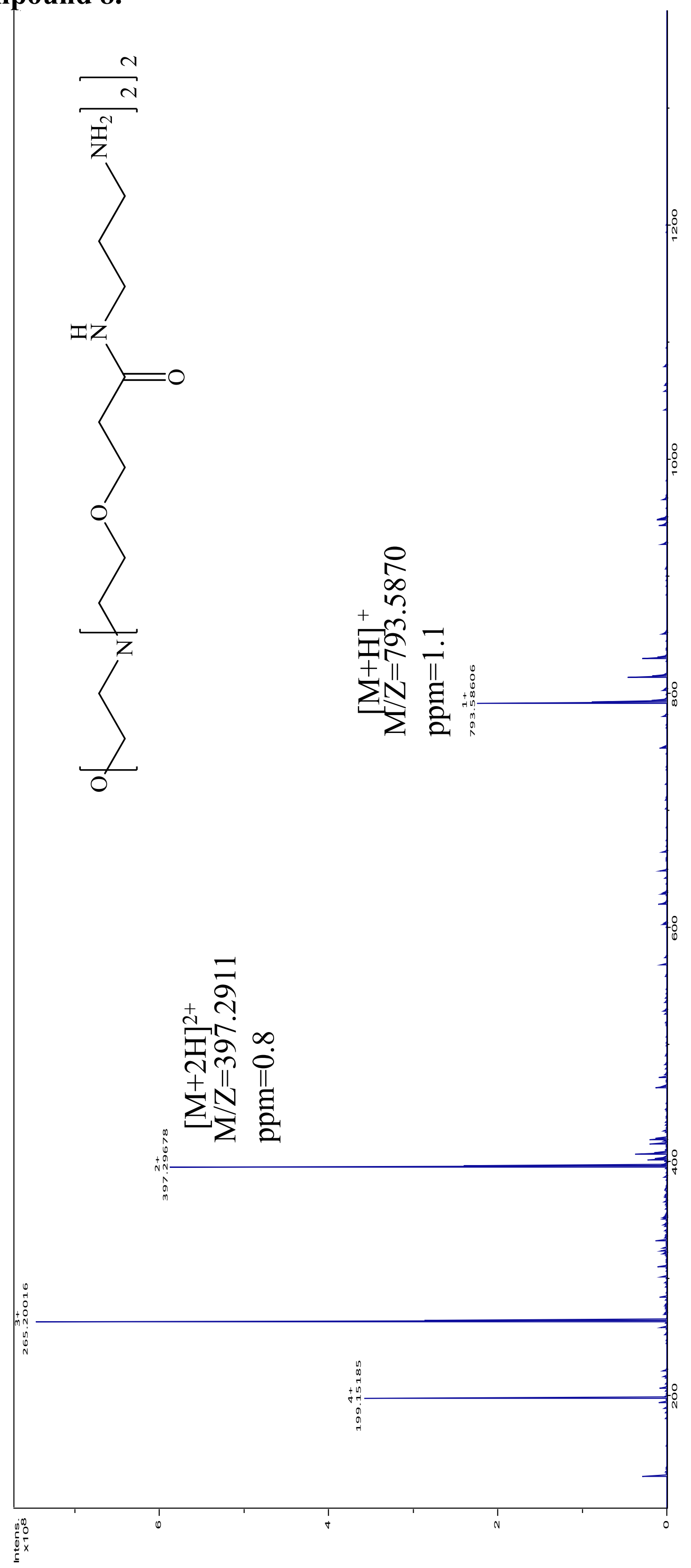

**Figure S52: HR-ESI$^+$ spectrum of Compound 9.**

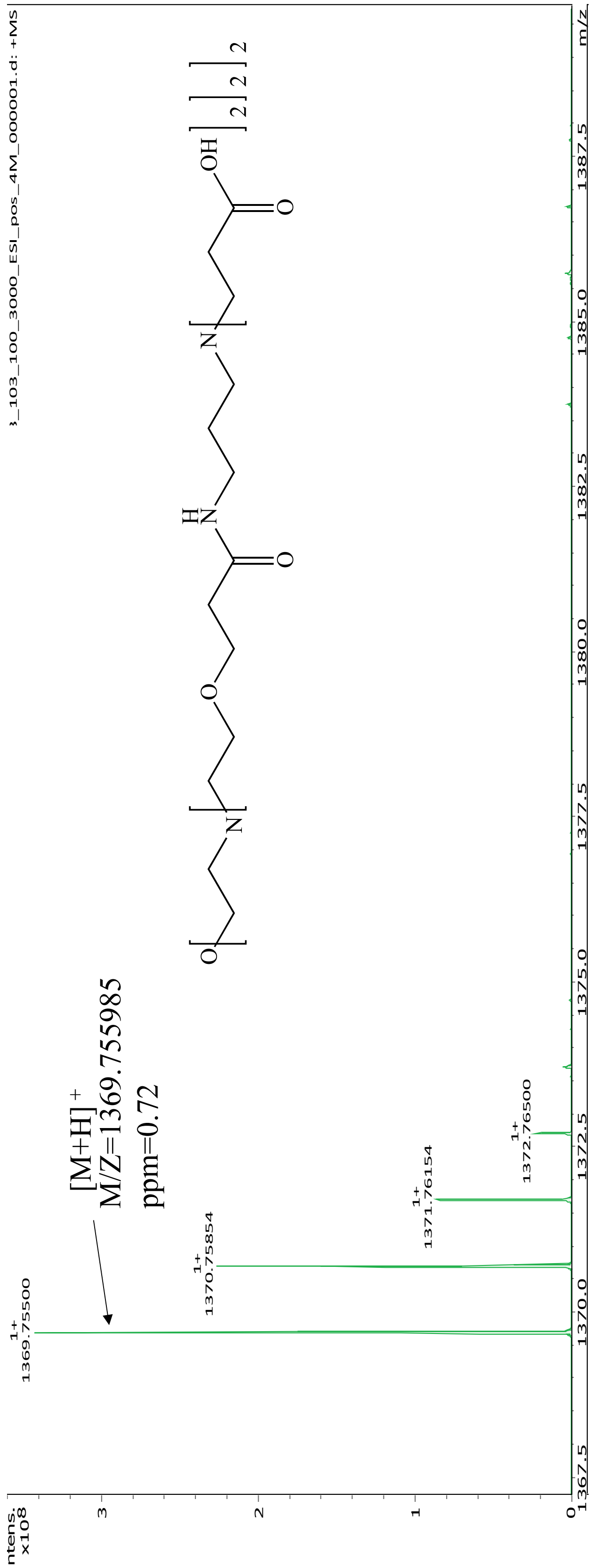

**Figure S53: HR-ESI$^+$ spectrum of Compound 10.**

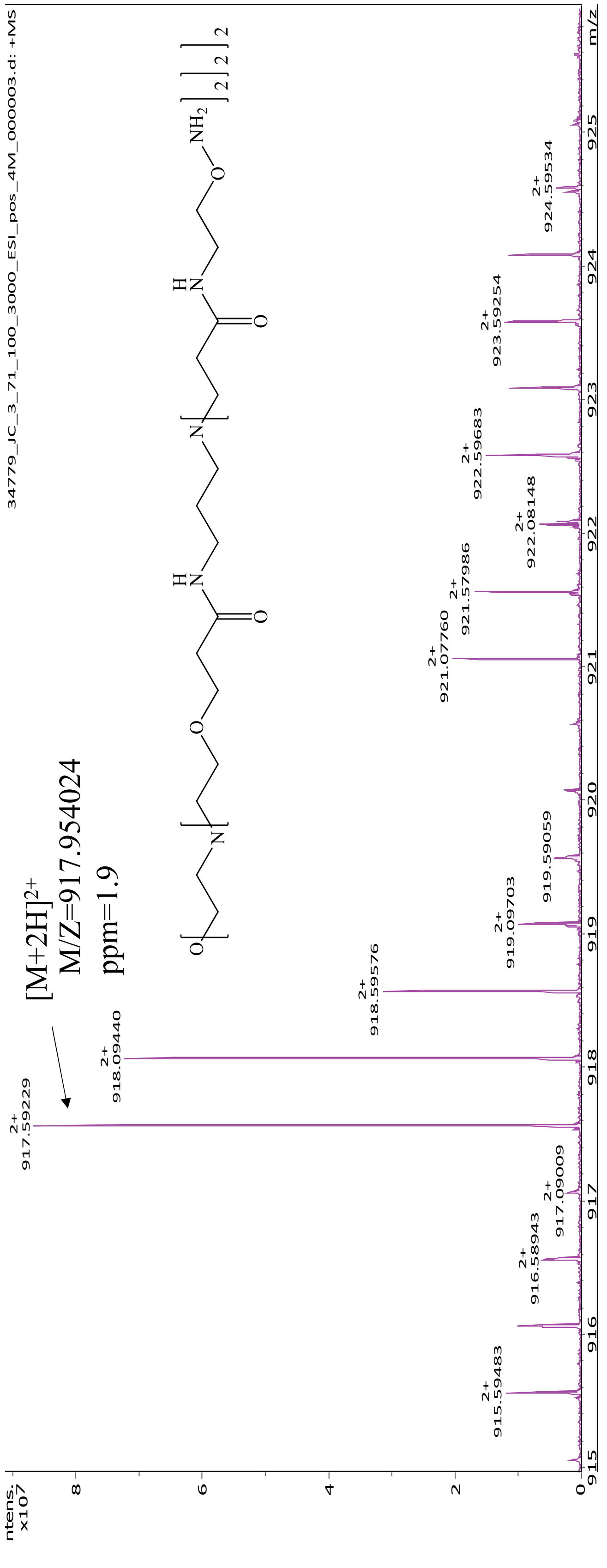

**Figure S54: HR-ESI$^{+}$ spectrum of Compound 11.**

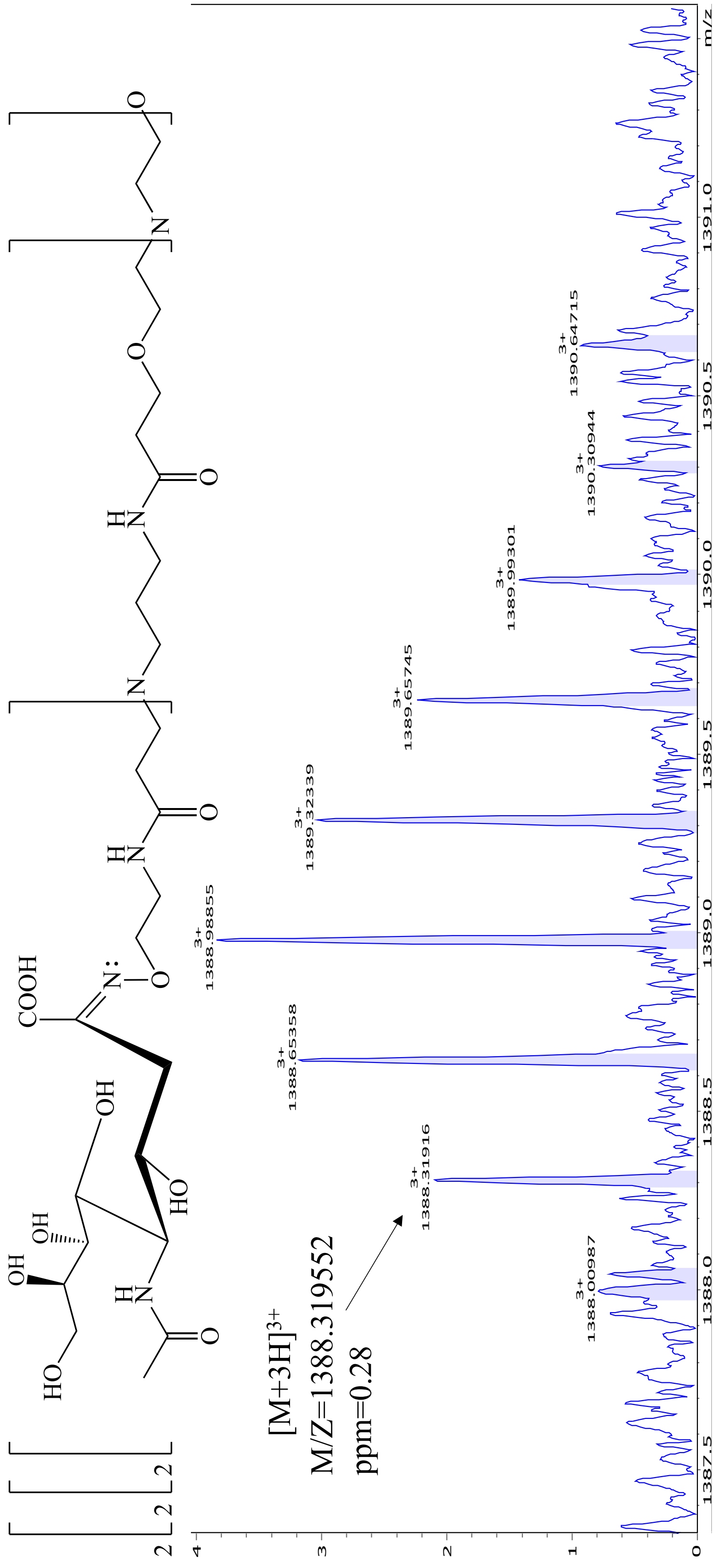

**Figure S55: FPLC chromatogram of Compound 11.**

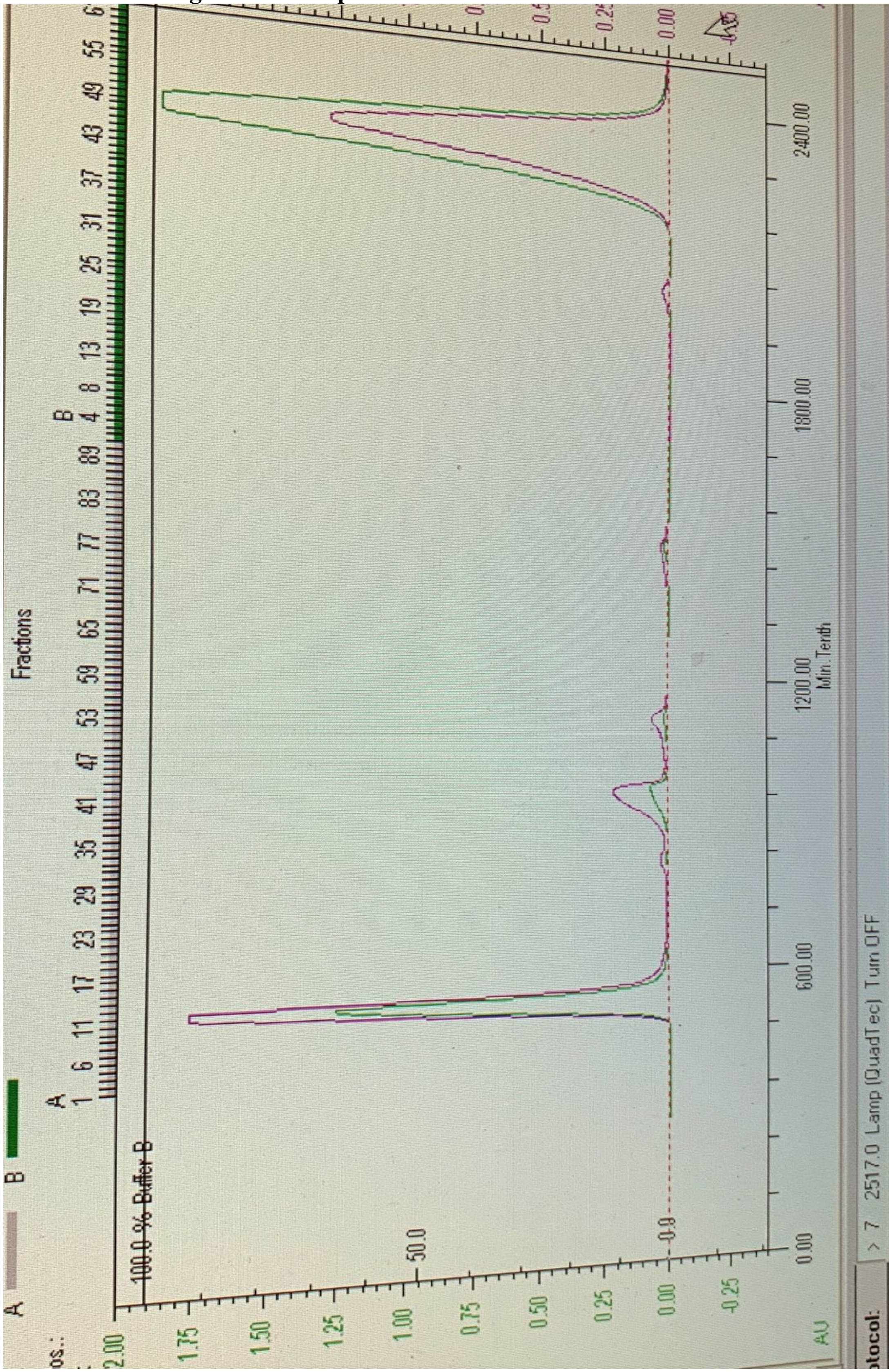

**Figure S56: FPLC chromatogram of Compound 12.**

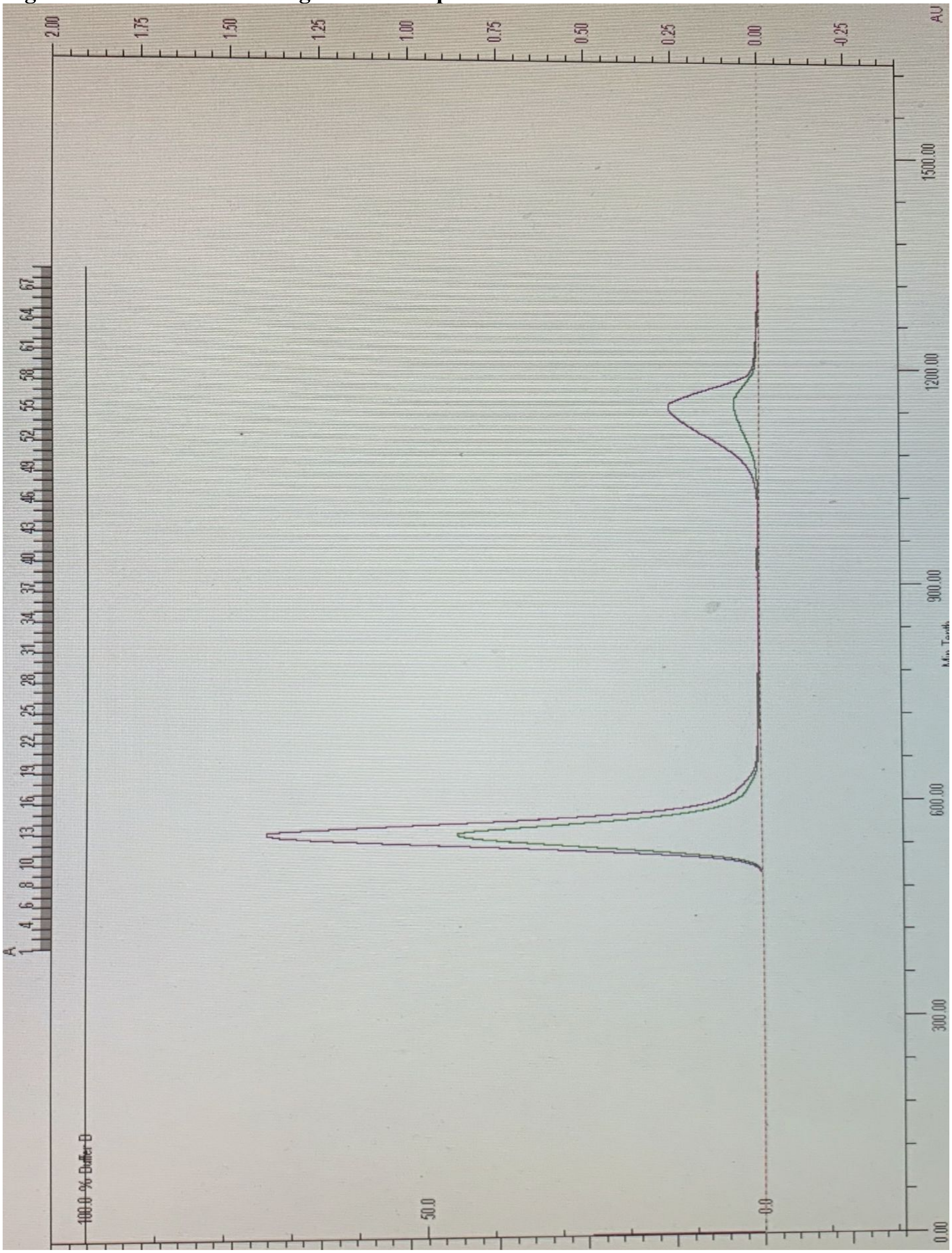

**Figure S57: FPLC chromatogram of Compound 13.**

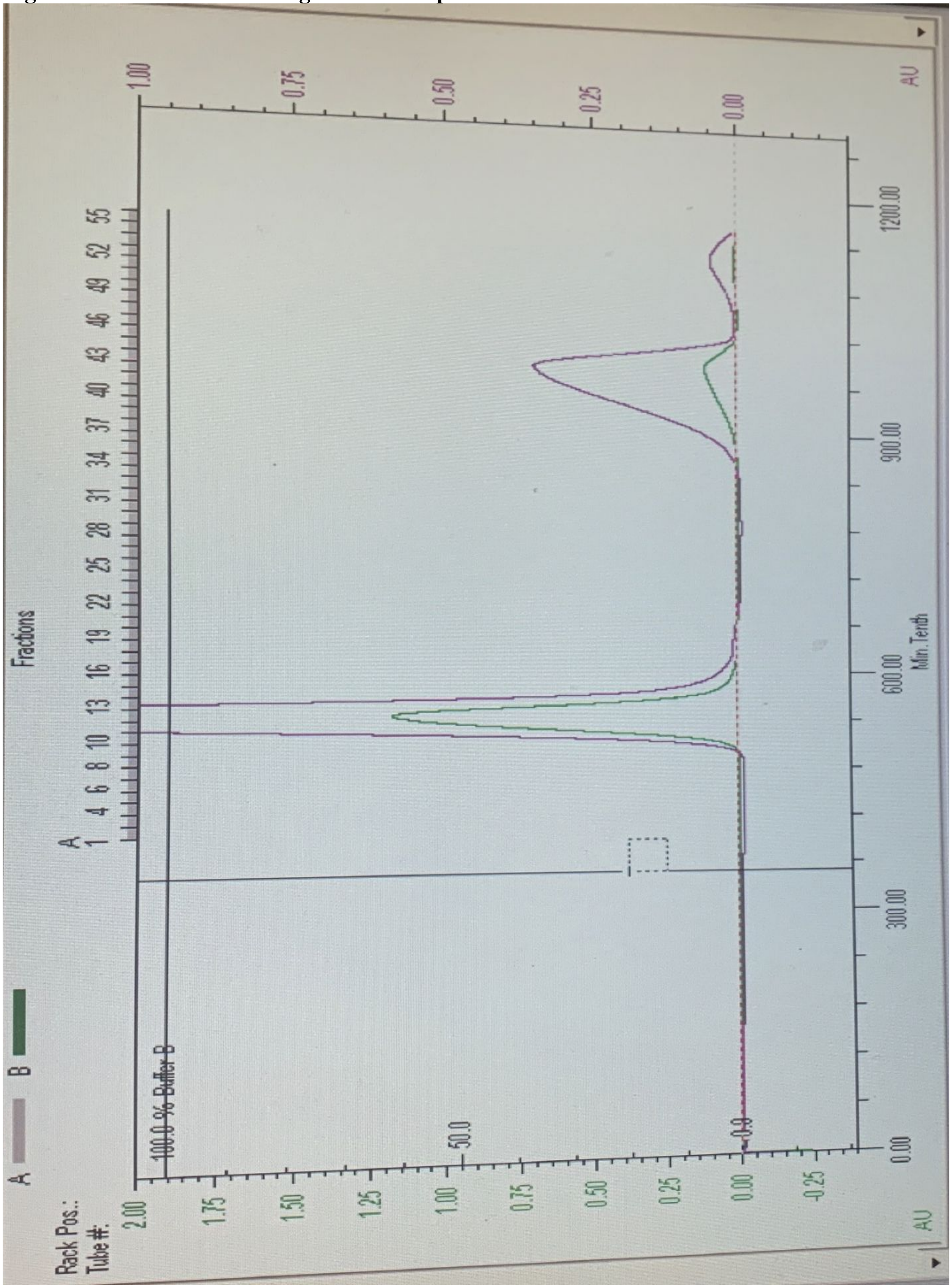

**Figure S58: FPLC chromatogram of Compound 14.**

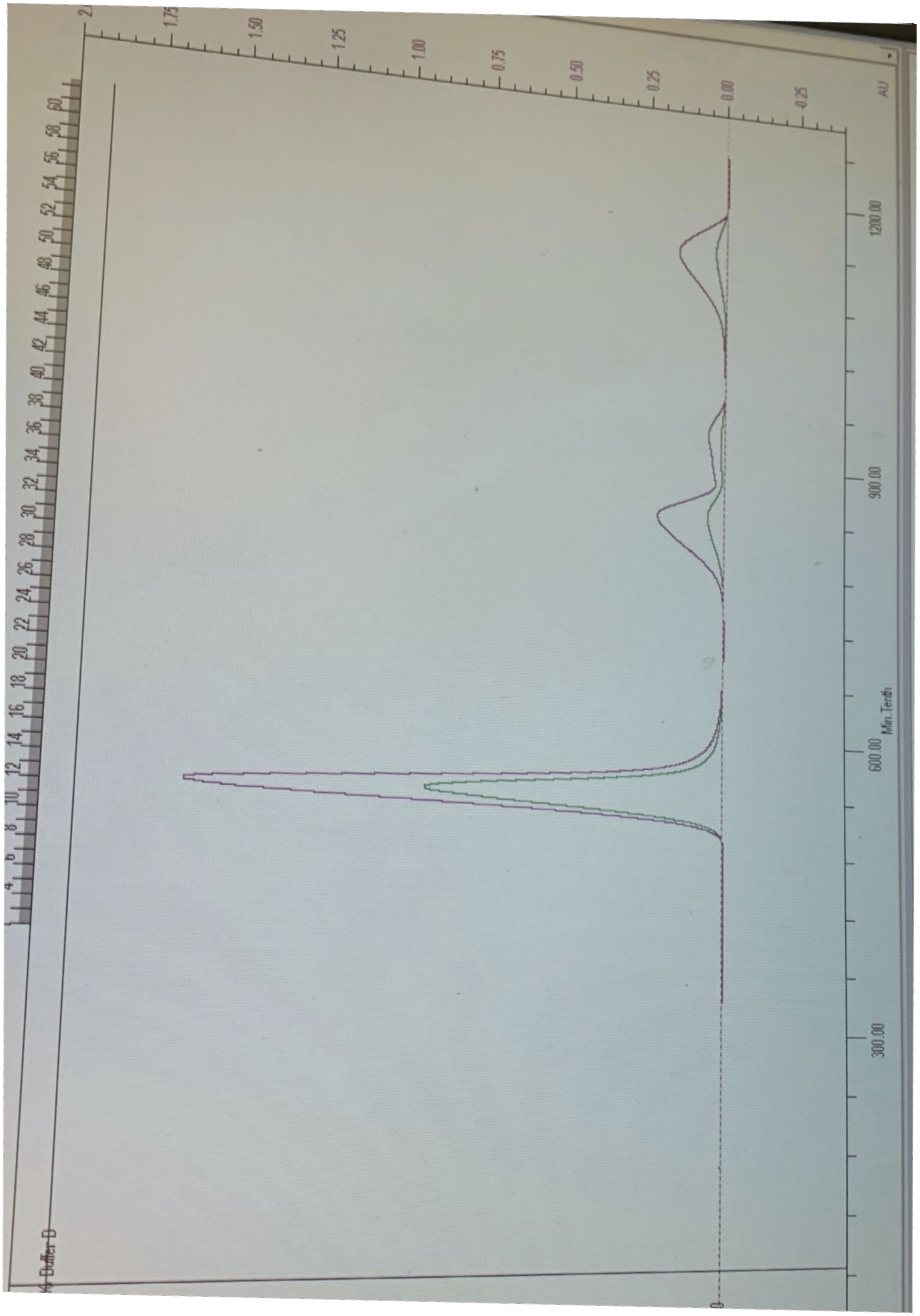

**Figure 59: SEC-MALS chromatogram of Compound12.**

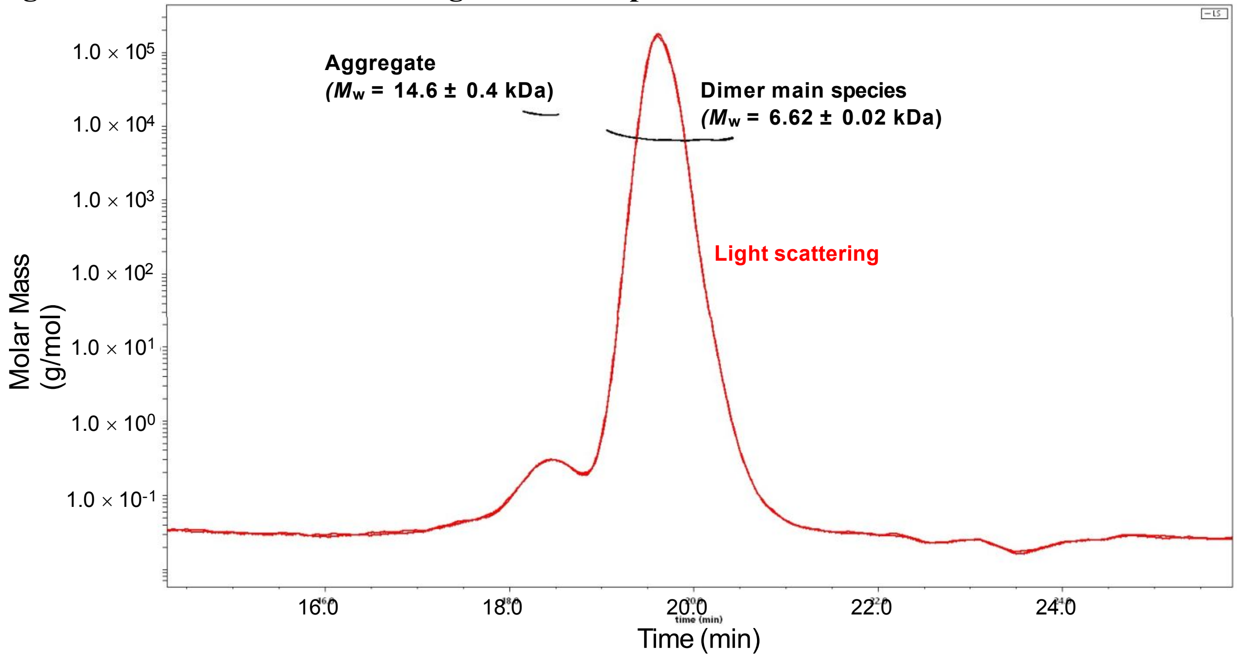

**Figure 60: SEC-MALS chromatogram of Compound13.**

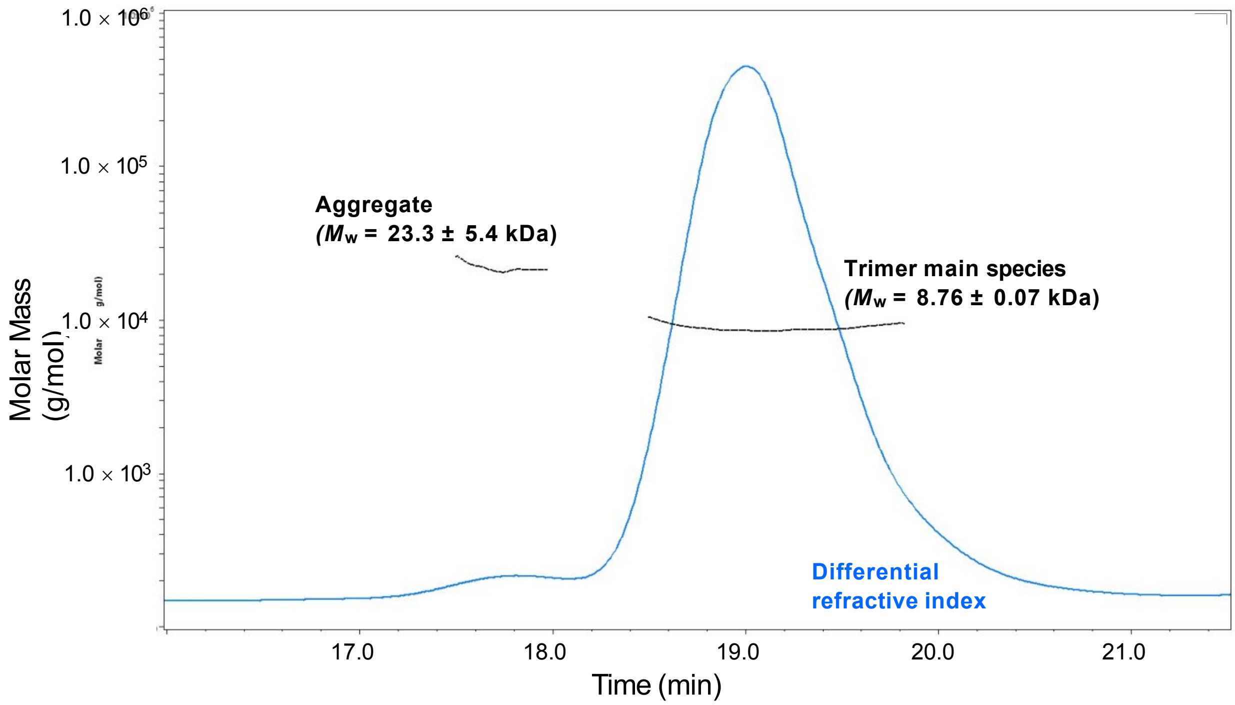

Supplement: Supplementary file 1 — bm3c00105_si_001.pdf [file bm3c00105_si_001.pdf]
